# Strain-specific differences in brain gene expression in a hydrocephalic mouse model with motile cilia dysfunction

**DOI:** 10.1038/s41598-018-31743-5

**Published:** 2018-09-06

**Authors:** Casey W. McKenzie, Claudia C. Preston, Rozzy Finn, Kathleen M. Eyster, Randolph S. Faustino, Lance Lee

**Affiliations:** 1 Pediatrics and Rare Diseases Group, Sanford Research, 2301 E. 60th Street N., Sioux Falls, SD 57104 USA; 2 Genetics and Genomics Group, Sanford Research, 2301 E. 60th Street N., Sioux Falls, SD 57104 USA; 3 Division of Basic Biomedical Sciences, Sanford School of Medicine of the University of South Dakota, Vermillion, SD 57069 USA; 4 Department of Pediatrics, Sanford School of Medicine of the University of South Dakota, 1400 W. 22nd Street, Sioux Falls, SD 57105 USA

## Abstract

Congenital hydrocephalus results from cerebrospinal fluid accumulation in the ventricles of the brain and causes severe neurological damage, but the underlying causes are not well understood. It is associated with several syndromes, including primary ciliary dyskinesia (PCD), which is caused by dysfunction of motile cilia. We previously demonstrated that mouse models of PCD lacking ciliary proteins CFAP221, CFAP54 and SPEF2 all have hydrocephalus with a strain-dependent severity. While morphological defects are more severe on the C57BL/6J (B6) background than 129S6/SvEvTac (129), cerebrospinal fluid flow is perturbed on both backgrounds, suggesting that abnormal cilia-driven flow is not the only factor underlying the hydrocephalus phenotype. Here, we performed a microarray analysis on brains from wild type and *nm1054* mice lacking CFAP221 on the B6 and 129 backgrounds. Expression differences were observed for a number of genes that cluster into distinct groups based on expression pattern and biological function, many of them implicated in cellular and biochemical processes essential for proper brain development. These include genes known to be functionally relevant to congenital hydrocephalus, as well as formation and function of both motile and sensory cilia. Identification of these genes provides important clues to mechanisms underlying congenital hydrocephalus severity.

## Introduction

Hydrocephalus is a complex disorder with both genetic and environmental causes^1^. It results from accumulation of cerebrospinal fluid (CSF) in the ventricles of the brain that typically leads to ventricular enlargement, damage to the underlying ependyma and white matter and thinning of the cerebral cortex^2,3^. CSF is produced by the choroid plexus and flows from the lateral ventricles through the third ventricle, the aqueduct of Sylvius and the fourth ventricle before entering the subarachnoid space and finally being absorbed into the venous system^4,5^. Whether the hydrocephalus is genetic or acquired, CSF can accumulate due to an obstruction in the ventricular system, such as a tumor or aqueductal stenosis, or a functional defect in CSF production, flow, or absorption^2,3,6,7^.

Little is currently known about the genetic basis of congenital hydrocephalus. To date, only three genes have been directly linked to non-syndromic hydrocephalus in human patients: cell adhesion molecule *L1CAM*^8^, Wnt pathway inhibitor *CCDC88C*^9,10^ and tight junction protein *MPDZ*^11,12^. Genetic mouse models have confirmed the role of *L1CAM*^13,14^ and *CCDC88C*^15^ in development of hydrocephalus and identified several additional genes that have yet to be linked to human hydrocephalus^16^. Hydrocephalus is also associated with a variety of genetic syndromes, including Dandy-Walker syndrome, Walker-Warburg syndrome, Noonan syndrome, Joubert syndrome and primary ciliary dyskinesia^1,7,16^.

Primary ciliary dyskinesia (PCD) results from defects in the function of motile cilia and flagella^17–19^. Affected individuals typically suffer from chronic rhinosinusitis, chronic otitis media and male infertility, with situs inversus, hydrocephalus and female infertility present in some cases^17–19^. Motile cilia on the ependymal cells that line the ventricular surface of the brain play an important role in facilitating the flow of CSF through the ventricular system and perturbations in flow can result in hydrocephalus^16,17^. Although hydrocephalus is only sporadically associated with PCD in patients, it is far more common in mouse models^16^. We have demonstrated that congenic mouse models of PCD lacking ciliary proteins CFAP221, CFAP54 and SPEF2 all have a severe hydrocephalus on the C57BL6/J (B6) background but not on 129S6/SvEvTac (129) or a mixed (B6x129)F1 background^20–22^, indicating strain specificity in susceptibility to severe PCD-associated hydrocephalus. While there are defects in cilia-driven fluid flow on both backgrounds, ventricular dilatation and secondary damage to surrounding brain tissue are consistently more severe in mutants on the B6 background^23^. These findings are consistent with reports of severe hydrocephalus in other PCD models on the B6 background and an absence of severe hydrocephalus in models on the 129 background^16^. Several non-PCD models, including mice with mutations in the genes encoding L1CAM^24^, the fyn tyrosine kinase^25,26^, alpha-N-acetylglucosaminidase^27^ and nuclear receptor NR2E1^28^, also have a more severe hydrocephalus when backcrossed to the B6 strain compared to other genetic backgrounds. In addition, strain-specific differences in brain anatomy and physiology have been observed that could account for variability in disease pathogenesis^29^. These studies suggest that genetic modifiers of hydrocephalus segregate in certain strains and influence susceptibility to severe hydrocephalus.

To begin to understand the genes and pathways underlying hydrocephalus susceptibility, we performed a DNA microarray analysis to identify strain-specific differences in gene expression in whole brains from wild type mice and *nm1054* mice lacking ciliary protein CFAP221 (previously known as PCDP1), as the phenotypic differences have been well characterized and are particularly dramatic in this line^20,23^. Similar approaches have been effective at identifying strain-specific candidate genes and mechanisms influencing a variety of murine phenotypes, including susceptibility to infection^30^, craniofacial defects^31^, hypertension^32^, eye pigmentation defects^33^, alcohol sensitivity^34^, cigarette smoke sensitivity^35^ and social behavior^36^. We analyzed gene expression levels in brains from wild type and *nm1054* mice on the B6 and 129 backgrounds and identified strain-specific expression levels for a number of genes that cluster into distinct groups based on expression profile and biological function. These genes are implicated in a variety of cellular and biochemical processes essential for proper brain development, including several with known relevance to hydrocephalus and ciliogenesis, providing the first insight into pathways that may underlie susceptibility to severe congenital hydrocephalus.

## Results

### Microarray analysis uncovers genes with strain-specific expression

Loss of *Cfap221* in *nm1054* mutant mice results in PCD characterized by hydrocephalus, male infertility and airway abnormalities due to ciliary dysfunction^20,23,37,38^. Although no strain-specific differences have been observed for the reproductive or respiratory phenotypes, the hydrocephalus is more severe on the B6 background than 129 despite defects in ependymal cilia-driven fluid flow on both backgrounds^23^. To identify gene expression differences that might influence susceptibility to severe hydrocephalus in *nm1054* mice on the B6 and 129 backgrounds, we utilized a DNA microarray approach. As ependymal ciliogenesis occurs during the first week of life^39,40^, the microarray analysis was performed at P1 to ensure that gene expression data are not influenced by the substantial tissue damage that can occur as a result of CSF accumulation on the B6 background when *nm1054* mice age^20,23^. Gross hydrocephalus was not observed in any mice at P1.

**Figure 1:**
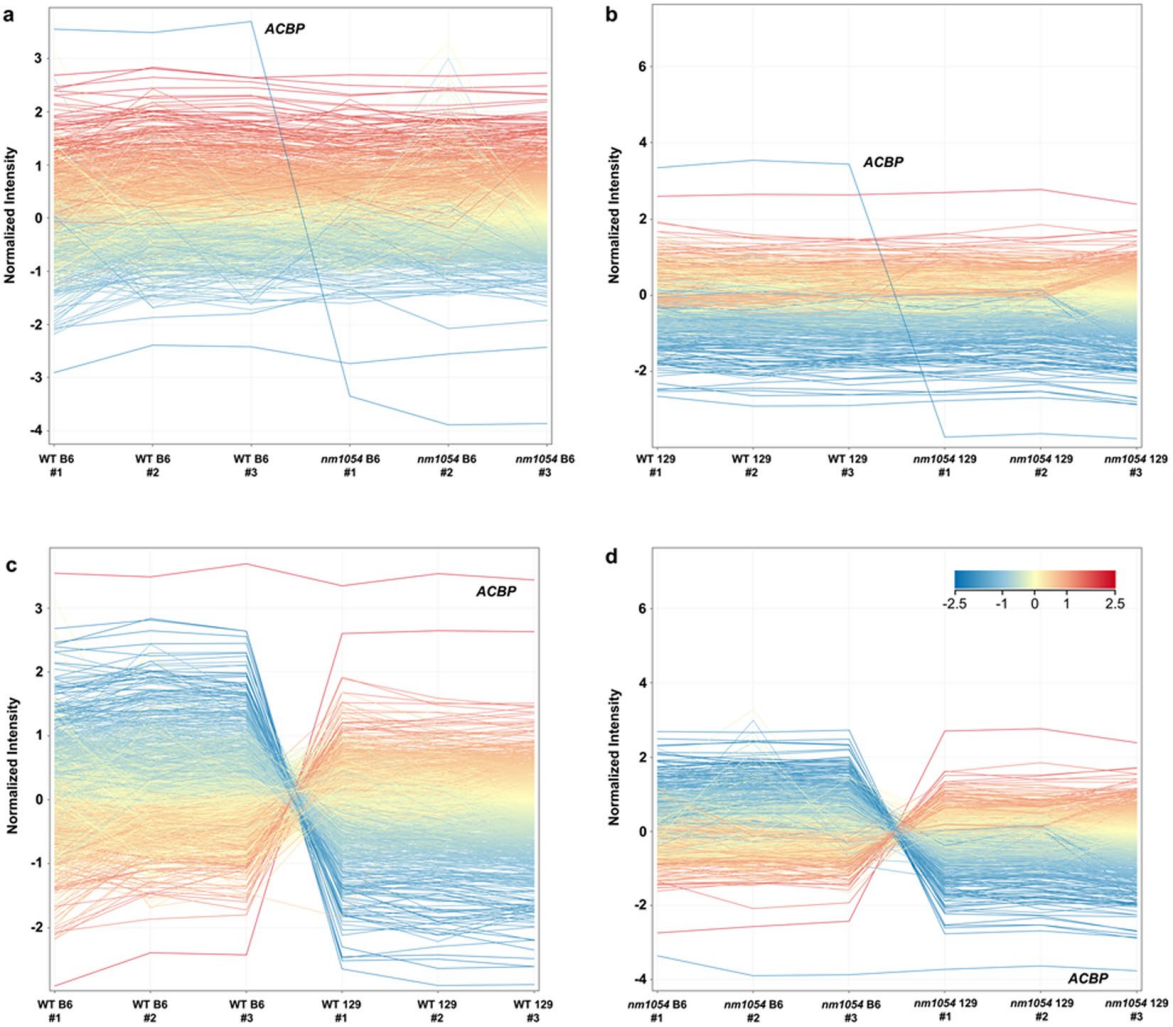
Line expression plots demonstrate differentially expressed transcripts in P1 mouse brains. A total of 42,855 entities were evaluated after quality control analysis, with most expressed at relatively similar levels in WT and *nm1054* brains on the B6 background (**a**) and 129 background (**b**). Only one gene deleted by the *nm1054* mutation (*Acbp*) is differentially expressed in the comparison of WT to *nm1054* brains (**a**,**b**). A large number of transcripts are differentially expressed between B6 and 129 brains in the WT comparison (**c**) and the *nm1054* comparison (**d**). Expression of *Acbp* is similar in the two strains (**c**,**d**).

RNA was isolated from whole brain, as the specific mechanisms underlying severe hydrocephalus remain unknown. DNA microarray analysis was performed on WT B6, WT 129, *nm1054* B6 and *nm1054* 129 samples to identify differences in gene expression between WT and *nm1054* mice, as well as between the B6 and 129 strains. There were 42,855 entities evaluated after QC analysis. Most genes are expressed at similar levels in WT and *nm1054* brains. Line expression plots comparing WT and *nm1054* samples on the B6 (Fig. 1a) and 129 (Fig. 1b) backgrounds show most transcripts expressed at nearly the same level in WT and *nm1054* samples. While some transcripts show variation between individual samples, only the transcript encoding the acyl CoA-binding protein (ACBP), also known as diazepam binding inhibitor (DBI), is consistently higher in the WT samples than the *nm1054* samples in both the B6 and 129 comparisons (Fig. 1a,b). The *Acbp* gene lies on mouse chromosome 1 within the *nm1054* deletion interval^20,37^, indicating that the microarray approach is effective at identifying expected differences in gene expression. Five additional genes encoding CFAP221, SCTR, STEAP3, TMEM37 and a novel protein represented by GenBank accession number NM_028439 are removed by the *nm1054* deletion. *Cfap221*, *Sctr*, *Steap3* and *Tmem37* expression was inconsistently present, marginal, or absent in the individual samples within sample groups, thereby preventing detection in the WT vs *nm1054* comparisons and *NM_028439* failed to pass QC analysis. To validate that the microarray was accurately detecting expected differences in gene expression, we confirmed that three of the genes deleted by the *nm1054* mutation (*Cfap221*, *Acbp* and *Sctr*) are not expressed in the *nm1054* brain relative to WT by quantitative RT PCR with RNA from equivalent P1 whole brain lysates (Supplementary Fig. S1).

In contrast to the comparison of WT and *nm1054* gene expression, the comparison of WT brains on the B6 and 129 backgrounds revealed that a large number of transcripts are expressed at substantially different levels between the two strains (Fig. 1c). A similar pattern is observed in the comparison of *nm1054* brains on the B6 and 129 backgrounds (Fig. 1d). In contrast to the differential expression of *Acbp* between WT and *nm1054* brains, the gene does not show a substantial difference between the B6 and 129 strains.

**Figure 2:**
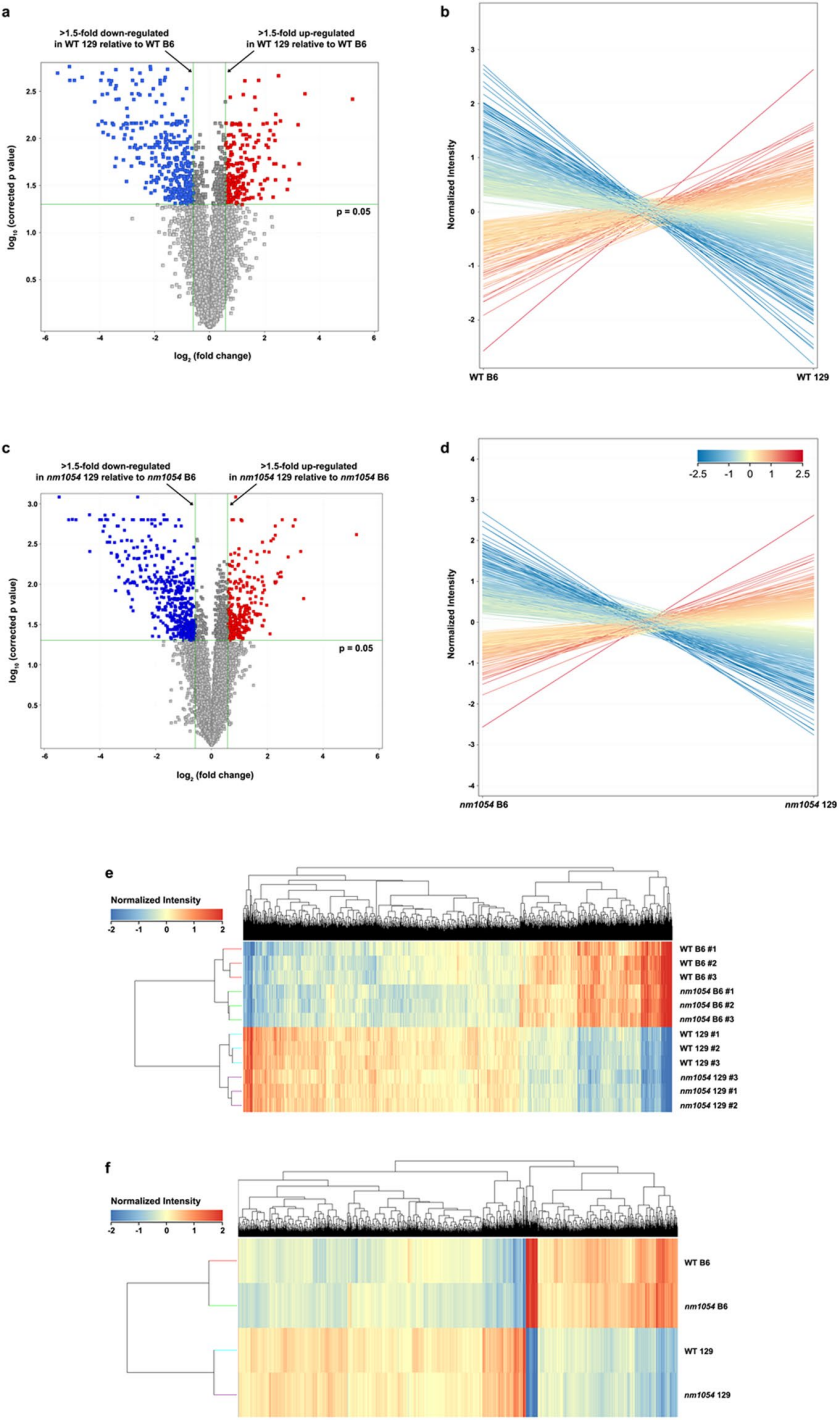
Statistical analysis distinguishes genes with significant strain-specific expression differences. ANOVA identified 2,805 transcripts with a corrected p value less than 0.05. Data for the WT comparison were filtered through a volcano plot to apply a 1.5-fold threshold (**a**) and the line expression plot shows the pool of differentially expressed genes between the WT B6 and WT 129 brains above the 1.5-fold threshold (**b**). Similarly, the *nm1054* comparison data were filtered through a volcano plot to apply the same 1.5-fold threshold (**c**), with the line expression plot showing the differentially expressed genes between the *nm1054* B6 and *nm1054* 129 brains above the applied threshold (**d**). Hierarchical clustering heat maps demonstrate differential expression profiles, with a comparison of individual sample profiles after filtering showing similar expression trends within sample groups (**e**). Combined profiles for the WT B6, *nm1054* B6, WT 129 and *nm1054* 129 groups reveal similar expression trends between WT and *nm1054* brains but more substantial variability between B6 and 129 brains (**f**).

Statistical analysis of the expression data using ANOVA identified 2,805 transcripts with a corrected p value less than 0.05. To eliminate transcripts that are least likely to have a biologically significant difference in strain-specific expression, the data were filtered by volcano plot analysis to apply a 1.5-fold threshold and all differences in expression that were below 1.5-fold were removed from the pools for both the WT (Fig. 2a) and *nm1054* (Fig. 2c) comparison. The line expression plots in Fig. 2 show the comparison of the combined B6 and 129 datasets after filtering out the transcripts below the 1.5-fold threshold. The only remaining transcripts for the WT (Fig. 2b) and *nm1054* (Fig. 2d) comparisons are those with consistent, statistically significant expression differences above the applied threshold.

A principal component analysis (PCA) plot shows the individual samples falling consistently into four distinct sample groups: WT B6, *nm1054* B6, WT 129 and *nm1054* 129 (Supplementary Fig. S2). A heat map showing transcript expression profiles for each individual sample after filtering on the volcano plot indicates that, while there are individual transcript differences, similar expression trends are observed within each sample group (Fig. 2e). The heat map in Fig. 2f shows the combined transcript expression profiles for the WT B6, *nm1054* B6, WT 129 and *nm1054* 129 groups. The combined profiles show similar expression trends between WT and *nm1054* regardless of genetic background. However, substantial variability in transcript expression is evident in the comparison of B6 and 129 samples, regardless of WT or *nm1054* comparison.

**Figure 3:**
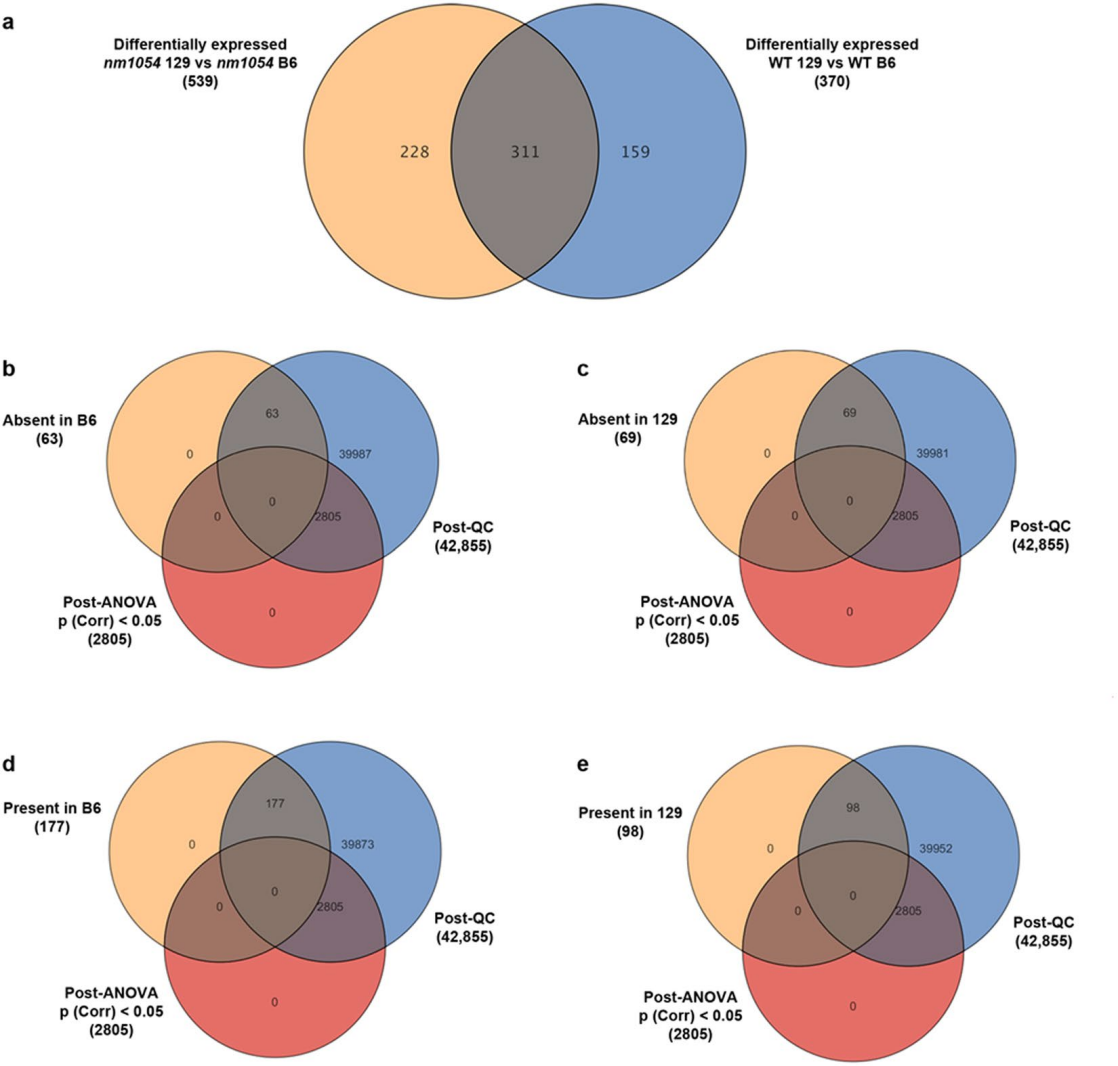
Venn diagrams demonstrate the number of differentially expressed genes. Following statistical analysis, 340 transcripts are differentially expressed between WT B6 and WT 129 brains and 539 transcripts are differentially expressed between *nm1054* B6 and *nm1054* 129, with 311 transcripts identified in both comparisons (**a**). Sixty-three transcripts were absent in all B6 samples but present or marginal in the 129 samples (**b**) and 69 transcripts were absent in all 129 samples but present or marginal in the B6 samples (**c**). Similarly, 177 transcripts were present in all B6 samples but absent or marginal in the 129 samples (**d**) and 98 transcripts were present in all 129 samples but absent or marginal in the B6 samples (**e**).

Following statistical analysis, a total of 340 transcripts showed greater than 1.5-fold expression differences between WT B6 and WT 129 brains (Supplementary Table S1) and 539 transcripts showed expression differences between *nm1054* B6 and *nm1054* 129 brains (Supplementary Table S2). Of those differentially expressed transcripts, 311 were identified in both the WT and the *nm1054* comparison (Fig. 3a), indicating that these expression differences are inherent to the strains and not influenced by the *nm1054* mutation. Only *Acbp* was identified as differentially expressed between WT and *nm1054* brains in the B6 and 129 comparisons (Supplementary Tables S3,S4). In addition to the differentially expressed genes, there were a number of genes that were absent in all samples from one strain and present or marginal in all samples from the other, as well as genes present in all samples from one strain and absent or marginal in all samples from the other. A total of 63 transcripts were absent in all B6 samples but present or marginal in the 129 samples (Fig. 3b, Supplementary Table S5) and 69 transcripts were absent in all 129 samples but present or marginal in the B6 samples (Fig. 3c, Supplementary Table S6). These transcripts would not appear in the analysis of differentially expressed genes and indicate that a subset of genes are actively expressed in one strain but not in the other. Similarly, 177 transcripts were present in all B6 samples but absent or marginal in the 129 samples (Fig. 3d, Supplementary Table S7) and 98 transcripts were present in all 129 samples but absent or marginal in the B6 samples (Fig. 3e, Supplementary Table S8). Although the large number of transcripts that are either differentially expressed between the two strains, present in only one strain, or absent in only one strain is likely due to the complexity of the tissue sample, the pool of transcripts is likely to include candidate genes for hydrocephalus susceptibility.

### Differentially expressed transcripts fall into distinct clusters and functional groups

**Figure 4:**
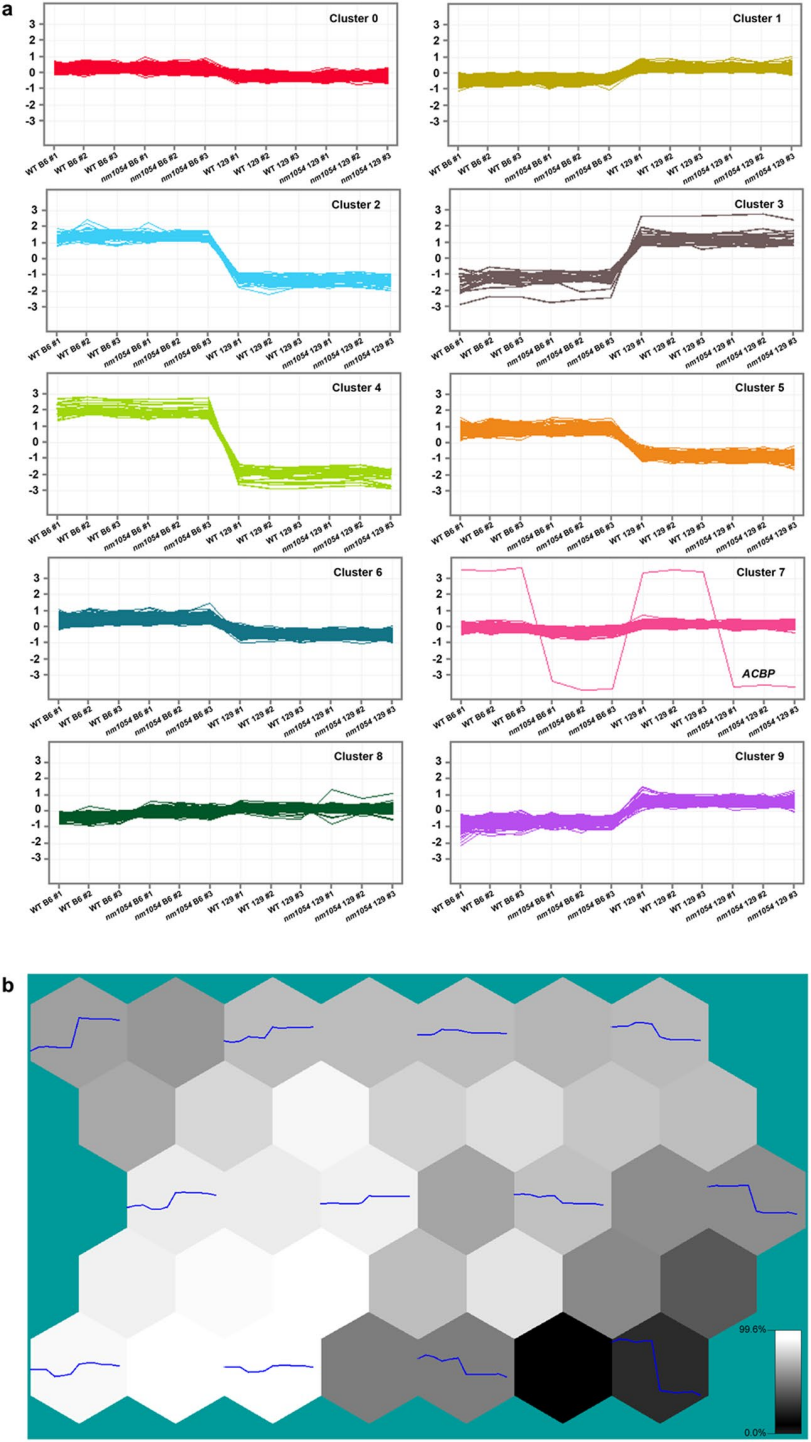
Gene clustering classifies distinct groups based on expression profiles. K-means clustering identifies ten groups with distinct expression trends (**a**). Three clusters (2, 4 and 5) show distinct trends of higher expression in B6 brains compared to 129, while two clusters (3 and 9) show distinct trends of higher expression in 129. U-Matrix view of gene expression meta-profiles for each group of genes identified by self-organizing map (SOM) clustering (**b**). Each hexagon, or node, containing a meta-profile (plotted in blue) is adjacent to a node that indicates the degree of similarity or dissimilarity to the following node and is depicted by a range of color from white (99.6% similarity) to black (0.0% similarity). The white connecting nodes between the expression profiles indicate clusters with a Euclidean distance metric close to 1.0, indicating high degree of similarity. The black or dark gray connecting nodes between the expression profiles indicate clusters with a Euclidean distance measure closer to 0 and are more dissimilar.

The transcripts were clustered using the GeneSpring software into ten groups defined by distinct expression trends (Fig. 4a). Three clusters (2, 4 and 5) show distinct trends of higher expression in B6 brains compared to 129, while two clusters (3 and 9) show distinct trends of higher expression in 129. The remaining groups show only minor expression differences between the two strains. Plotting meta-profiles for each of the clusters into a U-matrix revealed clusters that were similar or dissimilar in expression trend dynamics (Fig. 4b). In this analysis, highly similar profiles are separated by a white hexagon or node indicative of a Euclidean distance close to or equal to 1.0. Highly dissimilar profiles are separated by nodes colored in gray or black, which indicate Euclidean distance metrics that are between 0 and 1 or equal to 0, respectively.

**Figure 5:**
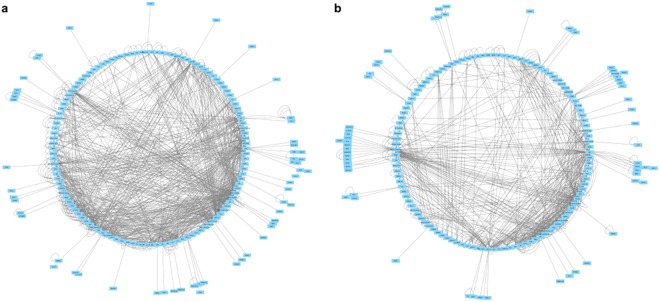
Functional enrichment analysis identifies a complex spectrum of molecular networks. Network diagrams with gene nodes in a circular layout demonstrates a greater number of hubs and connectivity between differentially expressed genes in the comparison of WT B6 to WT 129 (**a**) than the comparison of *nm1054* B6 to *nm1054* 129 (**b**), as indicated by the higher edge density in the WT comparison.

**Figure 6:**
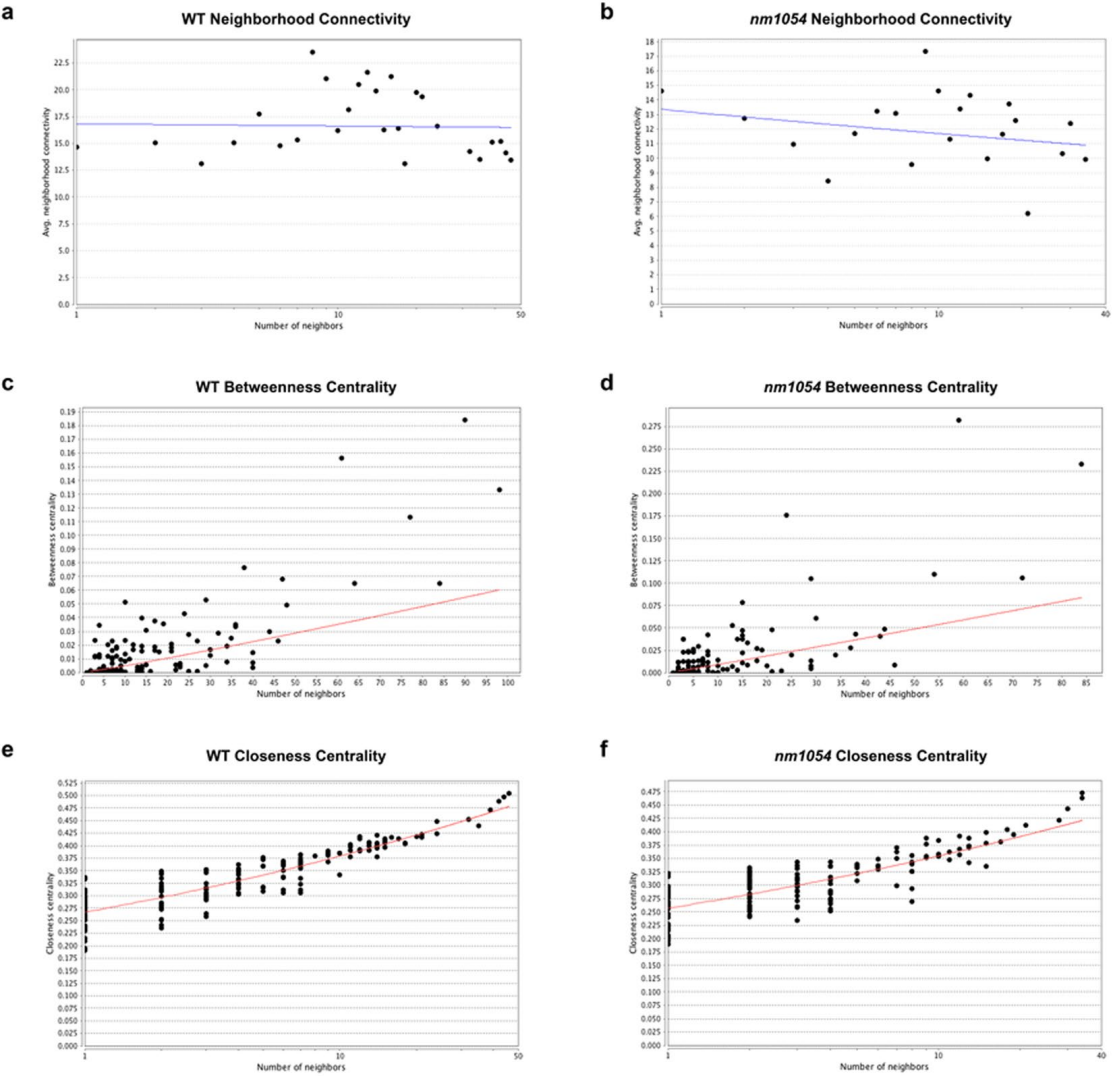
Connectivity within the molecular networks. Neighborhood connectivity remains relatively constant for the WT comparison (**a**) and indicates slight disassortativity in the *nm1054* comparison (**b**), suggesting that nodes with high degree form connections with nodes of low degree. Betweenness centrality positively correlates with number of neighbors for the WT comparison (**c**) and the *nm1054* comparison (**d**), with both comparisons showing several high-value nodes that exert a substantial force on other nodes in the network. Similarly, closeness centrality is also positively associated with number of neighbors for the WT comparison (**e**) and *nm1054* comparison (**f**), indicating that information spreads throughout the network at a greater rate through key nodes. Collectively, these metrics indicate a resilient, hierarchical network architecture with several high-value candidates that may control overall biological function.

The genes identified as differentially expressed between the B6 and 129 strains encode proteins with a wide variety of functions. Gene network analysis using the Ingenuity Pathway Analysis software showed the most prevalent molecular and cellular functions to be cell morphology, carbohydrate metabolism, small molecule biochemistry and cell-to-cell signaling and interaction in the WT comparison (Table 1). The top functions in the *nm1054* comparison were lipid metabolism, nucleic acid metabolism, small molecule biochemistry, cell morphology and carbohydrate metabolism. The analysis also demonstrated a functional enrichment in a diverse spectrum of molecular networks for both the WT and the *nm1054* comparison (Table 2, Fig. 5). Network diagrams with gene nodes in a circular layout reveal a higher edge density in the WT comparison (Fig. 5a) than the *nm1054* comparison (Fig. 5b), which indicates a greater number of node hubs. These networks suggest that there is greater connectivity between differentially expressed genes in the WT comparison. Neighborhood connectivity, which measures connection between hubs and is an indicator of network resiliency, remains fairly constant relative to the number of neighbors for both comparisons (Fig. 6a,b). The slight negative slope for the *nm1054* neighborhood connectivity indicates that the network has a slightly disassortative nature where highly connected nodes tend to connect to nodes with a lower degree, thus reflecting typical biological network architecture^41^. Analysis of betweenness centrality, which represents the control a node exerts on other nodes, reveals a positive association as the number of neighbors increases (Fig. 6c,d). Both comparisons show several nodes with high value, indicating a substantial effect on other nodes in the network. Analyses of closeness centrality indicate nodes that control the rate at which information spreads throughout the network and also show a positive association with number of neighbors in both the WT and *nm1054* comparisons (Fig. 6e,f), further corroborating the hierarchical network architecture of the dataset.

**Table 1:** Top Molecular and Cellular Functions Identified for Differentially Expressed Genes.

Functions Identified from WT Comparison^a^	p values^b^ (WT)	Number of Molecules (WT)	Functions Identified from *nm1054* Comparison^a^	p values^b^ (*nm1054*)	Number of Molecules (*nm1054*)
Cell Morphology	1.66E-02–6.16E-05	70	Lipid Metabolism	1.80E-02–1.12E-04	13
Carbohydrate Metabolism	1.62E-02–9.38E-05	25	Nucleic Acid Metabolism	1.80E-02–1.12E-04	10
Drug Metabolism	1.41E-02–9.38E-05	5	Small Molecule Biochemistry	1.80E-02–1.12E-04	38
Small Molecule Biochemistry	1.62E-02–9.38E-05	26	Cell Morphology	1.80E-02–1.30E-04	85
Cell-to-Cell Signaling and Interaction	1.67E-02–1.99E-04	32	Carbohydrate Metabolism	1.80E-02–1.93E-04	27

^a^The WT comparison is WT B6 vs WT 129 and the *nm1054* comparison is *nm1054* B6 vs *nm1054* 129.

^b^p values ranges for the identified molecules were calculated by the Ingenuity Pathway Analysis software.

**Table 2:** Top Protein Networks Identified for Differentially Expressed Genes.

Networks Identified from WT Comparison^a^	Likelihood Score^b^ (WT)	Networks Identified from *nm1054* Comparison^a^	Likelihood Score^b^ (*nm1054*)
Cell Cycle, Cellular Assembly and Organization, DNA Replication, Recombination and Repair	39	Molecular Transport, Cellular Function and Maintenance, Small Molecule Biochemistry	48
Protein Synthesis, Lipid Metabolism, Molecular Transport	37	Gastrointestinal Disease, Hematological Disease, Hepatic System Disease	41
Organismal Injury and Abnormalities, Free Radical Scavenging, Neurological Disease	37	Cell Morphology, Cellular Function and Maintenance, Nervous System Development and Function	36
Developmental Disorder, Neurological Disease, Psychological Disorders	37	Cell Cycle, Cellular Compromise, Skeletal and Muscular Disorders	34
Cellular Assembly and Organization, Cellular Function and Maintenance, Hair and Skin Development and Function	37	Cellular Assembly and Organization, Cellular Function and Maintenance, Hereditary Disorder	34

^a^The WT comparison is WT B6 vs WT 129 and the *nm1054* comparison is *nm1054* B6 vs *nm1054* 129.

^b^Network likelihood scores were calculated by the Ingenuity Pathway Analysis software.

## Discussion

In this study, we have demonstrated that a substantial number of transcripts are differentially expressed in brains from B6 and 129 mice at P1. Homozygous deletion mutants lacking CFAP221 (*nm1054*) have hydrocephalus associated with ciliary dysfunction that is more severe on the B6 background than 129 despite significant differences in ependymal cilia-driven flow on both backgrounds, indicating that genetic modifiers may influence the hydrocephalus phenotype^20,23^. DNA microarray analysis was performed using the whole brain, as the specific mechanisms underlying hydrocephalus severity remain unknown. Bioinformatics and functional enrichment analyses demonstrate that these genes cluster into distinct groups based on their expression profiles and biological function, many of which are implicated in critical cellular and biochemical processes essential for proper brain development. Because it is possible that the most biologically relevant genes may only be expressed in a small subset of cells, some of those genes could be masked by other genes that are expressed more ubiquitously or at generally higher levels. However, several of the genes identified in this study have a known relevance to hydrocephalus or ciliary function and may either represent those modifiers or be regulated by them. Identification of these genes therefore begins to provide clues to pathways underlying susceptibility to severe hydrocephalus.

Several genes were identified with direct relevance to hydrocephalus, either having at least a 1.5-fold expression difference between the B6 and 129 strains with a p value less than 0.05 or being expressed in only one of the two strains. Two of the known human hydrocephalus genes, *CCDC88C* and *MPDZ*, were identified in the microarray. Mutations in the Wnt pathway inhibitor gene *CCDC88C* (2.57-fold higher expression in B6, but only differentially expressed in the *nm1054* comparison) have been shown to result in non-syndromic congenital hydrocephalus in human patients^9,10^ and a mouse model^15^. Mutations in the tight junction gene *MPDZ* (absent in the 129 brain but present or marginal in B6 brains) also result in non-syndromic congenital hydrocephalus in patients^11,12^. In addition, loss of RND3 (3.21-fold higher in B6, but only differentially expressed in the *nm1054* comparison), which regulates cytoskeletal organization and cell adhesion, results in congenital hydrocephalus in mice due to altered Notch signaling^42^. *CDH2* (present in B6 brains but absent or marginal in 129) encodes N-cadherin, which forms cell-cell adherens junctions in the brain^43^. Blocking N-cadherin junctions *ex vivo* results in apoptosis of ciliated ependymal cells and damage to the ventricular wall^44^.

Both motile and primary cilia play a critical role in brain development and physiology^16,45,46^ and defects in both have been shown to result in hydrocephalus. Centrioles serve as the basis for formation of centrosomes and the basal bodies from which cilia and flagella extend^47–49^ and several genes involved in centriolar and centrosomal function were identified in the microarray analysis. Knockdown of centrosomal gene *CEP162* (higher expression in B6 brains, with a 2.20-fold difference in the WT comparison and 2.25-fold in the *nm1054* comparison) prevents ciliary transition zone assembly and primary ciliogenesis in cultured cells and results in hydrocephalus in zebrafish^50^. Knockdown of centrosomal gene *SNX10* (2.66-fold higher expression in 129 brains, but only differentially expressed in the WT comparison) also impairs ciliogenesis in both zebrafish and cultured cells^51^. *PCM1* (2.29-fold higher in B6 brains, but only differentially expressed in the *nm1054* comparison) encodes a centriolar satellite protein that interacts with several key regulators of centrosomal function and ciliogenesis to promote primary cilia formation and neuronal differentiation and migration^52–57^. Heterozygosity for a targeted allele of *PCM1* in mice results in reduced brain volume and behavioral abnormalities^58^. *FGFR1OP*, also known as *FOP* (higher in 129 brains, with a 2.19-fold difference in the WT comparison and 2.08-fold in the *nm1054* comparison), also encodes a centriolar satellite protein and knockdown in RPE-1 cells prevents formation of primary cilia^59^. *PLK1* (higher in B6 brains, with a 3.58-fold difference in the WT comparison and 3.27-fold in the *nm1054* comparison) encodes a kinase recruited by PCM1 that plays a role in centrosome maturation and primary cilia disassembly^57,60–64^. MDM1 (present in B6 brains but absent in or marginal in 129) functions as a negative regulator of centriole duplication and is upregulated during ciliogenesis^65,66^. A nonsense mutation in *MDM1* in a mouse model results in retinal degeneration^67^, a common hallmark of primary ciliopathies. Additionally, NUBP2 (higher in 129 brains, with a 2.17-fold difference in the WT comparison and 2.36-fold in the *nm1054* comparison), a nucleotide-binding protein that localizes to the centriole and the basal bodies of primary and motile cilia, functions as a negative regulator of ciliogenesis^68^.

Several additional genes involved in cilia assembly were identified. ELMO1 (present in B6 brains but absent or marginal in 129) regulates basal body migration and docking at the cell surface and knockdown results in ciliary phenotypes in *Xenopus* and zebrafish^69^. EHD3 (higher in B6 brains, with a 2.71-fold difference in the WT comparison and 2.34-fold in the *nm1054* comparison) plays a role in promoting ciliary vesicle formation and primary ciliogenesis in cultured cells and zebrafish^70^. Intraflagellar transport protein IFT74 (absent in 129 but present or marginal in B6 in the *nm1054* comparison only) forms a tubulin-binding module with IFT81 required for mammalian ciliogenesis^71^ and loss in *Chlamydomonas reinhardtii* perturbs flagellar assembly^72^. *MKKS*, also known as *BBS6* (higher in B6 brains, with a 3.96-fold difference in the WT comparison and 3.86-fold in the *nm1054* comparison), encodes a component of a protein complex that mediates BBSome complex assembly during ciliogenesis^73^. Mice lacking *MKKS* possess cilia but have a phenotype resembling the primary ciliopathy Bardet Biedl syndrome^74^. Biochemical and genetic interactions have been reported with centrosomal protein NPHP6, also known as CEP290, which plays a critical role in promoting primary ciliogenesis and has been implicated in multiple ciliopathies^75–77^. NPHP6 was also found to regulate ATF4 (higher in B6 brains, with a 6.36-fold difference in the WT comparison and 6.33-fold in the *nm1054* comparison), a transcription factor involved in multiple cellular stress pathways^77,78^. NPHP3 (present in 129 but absent or marginal in B6) localizes to primary cilia and human mutations in the *Nphp3* gene result in the primary ciliopathy nephronopthisis in humans and mice^79^. *ARL3* (higher in B6 brains, with a 7.10-fold difference in the WT comparison and 8.22-fold in the *nm1054* comparison) encodes a GTPase involved in trafficking of proteins to the primary cilium during ciliogenesis^80–82^. *WDR92* (2.09-fold higher in B6 brains in the WT comparison only) encodes a cytoplasmic chaperone involved in ciliary assembly and knockdown in planaria perturbs ciliary motility^83^. Mutations in *MYO7A* (higher in B6 brains, with a 6.37-fold difference in the WT comparison and 5.85-fold in the *nm1054* comparison), which encodes myosin VIIA, result in the primary ciliopathy Usher syndrome^84^ and loss of *MYO7A* in the *shaker1* mouse model results in abnormal organization of hair cell stereocilia^85,86^. Mutations in *SPAG1* (2.04-fold higher in 129 brains in the WT comparison, but higher in B6 brains in the *nm1054* comparison with only a 1.62-fold difference), which is required for assembly of axonemal dynein arms for motile cilia, result in PCD in human patients^87^. Finally, the extracellular matrix protein SPARC (higher in B6 brains, with a 5.74 fold difference in the WT comparison and 5.97-fold in the *nm1054* comparison) interacts with ciliary microtubules in *Xenopus* embryos^88,89^ and it has been suggested to play a role in CSF physiology^90^.

While similar transcript expression trends were observed within each sample group, there are some transcript expression differences between individual samples within each group (Fig. 2e). There are several factors that could contribute to these differences: 1) individual genetic variation, 2) subtle differences in the *in utero* environment and 3) technical variation due to the sensitivity level of the microarray approach. Despite these differences, the overall similarity of the expression profiles within each group is in stark contrast to the substantially distinct patterns between the B6 and 129 strains (Fig. 2f).

Further studies are required for validation of genes identified in this study and their direct involvement in influencing susceptibility to congenital hydrocephalus. Analysis of more specific brain regions and cell types, as well as additional time points, will aid in the functional refinement of these gene lists. Mapping and identification of modifier polymorphisms will provide additional evidence that, when combined with the gene expression data, will uncover the molecular mechanisms influencing this phenotype. It remains unclear whether the genetic modifiers specifically influence hydrocephalus associated with ependymal cilia dysfunction or whether they more broadly influence congenital hydrocephalus. While strain-specific trends in hydrocephalus severity have been observed for a number of PCD models^16^, several non-PCD models have exhibited a more severe hydrocephalus on the B6 background than other strains^24,26–28^. Future studies investigating candidate gene expression and sequence in other PCD and non-PCD models on relevant genetic backgrounds will uncover the full spectrum of genetic influence on congenital hydrocephalus. It is possible that specific genetic modifiers segregating in inbred mouse strains also influence susceptibility to severe hydrocephalus in the human population, providing hope that these mouse models will serve as powerful tools to uncover disease mechanisms and provide clues to aid in diagnosis and pharmacological treatment of congenital hydrocephalus.

## Methods

### Mice

All experiments involving animals were performed in accordance with the Animal Welfare Act and National Institutes of Health (NIH) policies and were approved by the Sanford Research Institutional Animal Care and Use Committee. All methods were carried out in accordance with applicable international, national and institutional guidelines for the care and use of animals. The *nm1054* line was maintained on the B6 and 129 backgrounds as previously described^20,23,38^. The spontaneous and heritable *nm1054* mutation is an approximately 400 kb deletion that removes six genes on mouse chromosome 1^20,37^. The PCD phenotype, including hydrocephalus, results exclusively from loss of *Cfap221* as previously demonstrated by transgenic rescue^20,37^. Mice were used for all analyses at postnatal day one (P1). Because mutants also have a severe and lethal anemia on the B6 background due to loss of the *Steap3* gene, we analyzed transgenic B6 mutants expressing the RPCI-22 bacterial artificial chromosome 11D19 containing *Steap3*^37^.

### Microarray analysis

Brains were removed from WT and *nm1054* homozygous mice on the B6 and 129 backgrounds at P1 (n = 3 in each group), snap frozen in liquid nitrogen and stored at −80 °C until RNA extraction. Each experimental group consisted of a pool of male and female mice, as no sex-specific differences have been observed in the *nm1054* hydrocephalus phenotype^20,23^. Total RNA was extracted from the whole brain with TRIzol and purified using the Maxwell 16 LEV simplyRNA Tissue Kit (Promega, Madison, WI) according to the manufacturer’s instructions. Labeled cRNA was prepared from 500 ng of total RNA using the Illumina RNA Amplification Kit (Ambion, Austin, TX). A total of 1,500 ng of labeled cRNA was hybridized overnight at 58 °C onto the MouseWG-6 Expression BeadChip (Illumina, San Diego, CA) according to the manufacturer’s instructions. These chips contain 45,281 transcripts representing 20,880 unique Entrez genes. Following hybridization, the chips were washed and developed with fluorolink streptavidin-Cy3 (GE Healthcare, Little Chalfont, UK) and scanned with an Illumina BeadArray Reader.

### Gene expression data analysis

Intensity values for each probe cell in the hybridized arrays were calculated by GenomeStudio software (Illumina Inc., San Diego, CA) and flags were assigned to each probe set declaring a Present, Marginal, or Absent call (Detection Call Algorithm). Probe cell intensities were used to calculate an average intensity for each set of probe pairs representing a gene, which directly correlated with the amount of cRNA. Further gene expression data analysis and normalization were performed using the GeneSpring GX bioinformatics software suite (Agilent Technologies, Palo Alto, CA). Quality control (QC) filtering was performed on the normalized intensity values, initially excluding the probe sets with an absent call in one hundred percent of the arrays. Alternate analyses were also performed using absent vs. present/marginal call flags or present vs. absent/marginal call flags to analyze genes with absent expression in one group compared to another. After applying QC filtering to diminish background noise created by low-intensity gene probes, genes were clustered into four conditions (WT 129, *nm1054* 129, WT B6 and *nm1054* B6). Data were organized into a hierarchically clustered heat map to elucidate gene expression profiles for each condition and visualized as volcano plots to identify genes significantly up- or down-regulated in each group. Functional enrichment analysis was performed on filtered gene lists of differentially expressed genes using the Ingenuity Pathway Analysis (IPA) software (Qiagen, Venlo, Netherlands).

### Quantitative RT PCR

Quantitative reverse transcription polymerase chain reaction (qRT PCR) was performed using the TaqMan approach. Total RNA was extracted from 8 B6 WT, 8 B6 *nm1054*, 8 129 WT and 6 129 *nm1054* brains, all of which were distinct from those used for microarray analysis, as described above. RNA integrity was evaluated using a 2100 Bioanalyzer (Agilent Technologies, Santa Clara, CA) and cDNA was synthesized from 1 μg RNA using the GoScript Reverse Transcription System (Promega). Quantitative PCR (qPCR) was performed in a Stratagene Mx3000P qPCR system (Agilent Technologies) as previously described^22^. Commercially available assays (Primetime Assays, IDT, Coralville, IA) were used for *Acbp, Cfap221* and *Sctr* and each was normalized to β-actin. Relative gene expression data were analyzed by the delta-delta Ct method^91^.

### Statistical analysis

Statistical analysis of the microarray gene expression was performed using unpaired *t*-test and a multiple testing correction formula, the Benjamini Hochberg false discovery rate, which together reported a corrected (Corr) *p* value for each gene. The hierarchical clustering for groups and entities was performed using Euclidean distance metric and Ward’s linkage algorithm. Statistical significance was set at fold change >1.5 and *p* (Corr) < 0.05. The qRT PCR data were analyzed by student’s t test using the GraphPad Prism software (GraphPad Software, La Jolla, CA).

## Electronic supplementary material

Supplementary Information

## Data Availability

The microarray datasets generated and analyzed in this study are available in the NCBI Gene Expression Omnibus (GEO) database under accession number GSE113233.
