# Supplementary material for: Strain-specific differences in brain gene expression in a hydrocephalic mouse model with motile cilia dysfunction
Source: Sci Rep. 2018 Sep 6;8:13370. doi: 10.1038/s41598-018-31743-5 (PMC6127338; doi:10.1038/s41598-018-31743-5)
Supplement: Supplementary file 1 — Supplementary Information [file 41598_2018_31743_MOESM1_ESM.pdf]

Title: Strain-specific differences in brain gene expression in a hydrocephalic mouse model with motile cilia dysfunction

Authors: Casey W. McKenzie[1], Claudia C. Preston[2], Rozzy Finn[1], Kathleen M. Eyster[3], Randolph S. Faustino[2,4], and Lance Lee[1,4,*]

Affiliations:

[1]Pediatrics and Rare Diseases Group, Sanford Research, 2301 E. 60th Street N., Sioux Falls, SD 57104

[2]Genetics and Genomics Group, Sanford Research, 2301 E. 60th Street N., Sioux Falls, SD 57104

[3]Division of Basic Biomedical Sciences, Sanford School of Medicine of the University of South Dakota, Vermillion, SD 57069

[4]Department of Pediatrics, Sanford School of Medicine of the University of South Dakota, 1400 W. 22nd Street, Sioux Falls, SD 57105

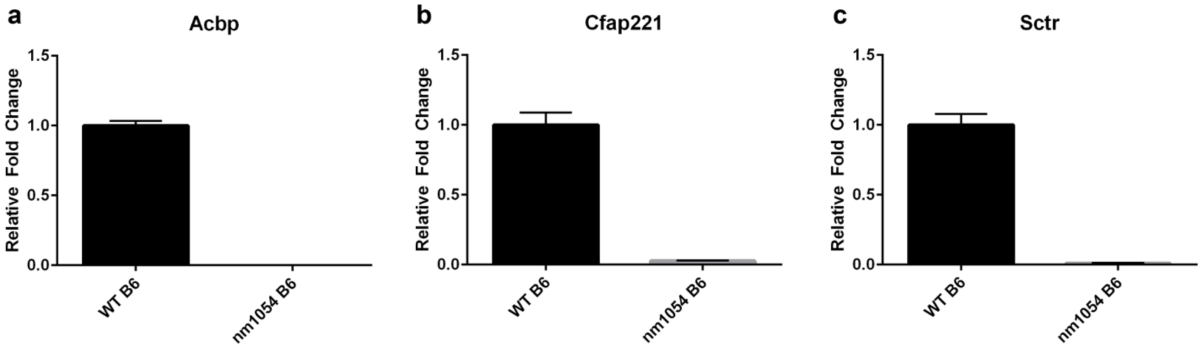

Supplementary Figure S1. Genes deleted by the *nm1054* mutation are not expressed in *nm1054* brains. Comparisons of WT and *nm1054* brains on the B6 background demonstrate that deleted genes *Acbp* (a), *Cfap221 (*b), and *Sctr (*c) are not expressed in the mutants by qRT PCR.

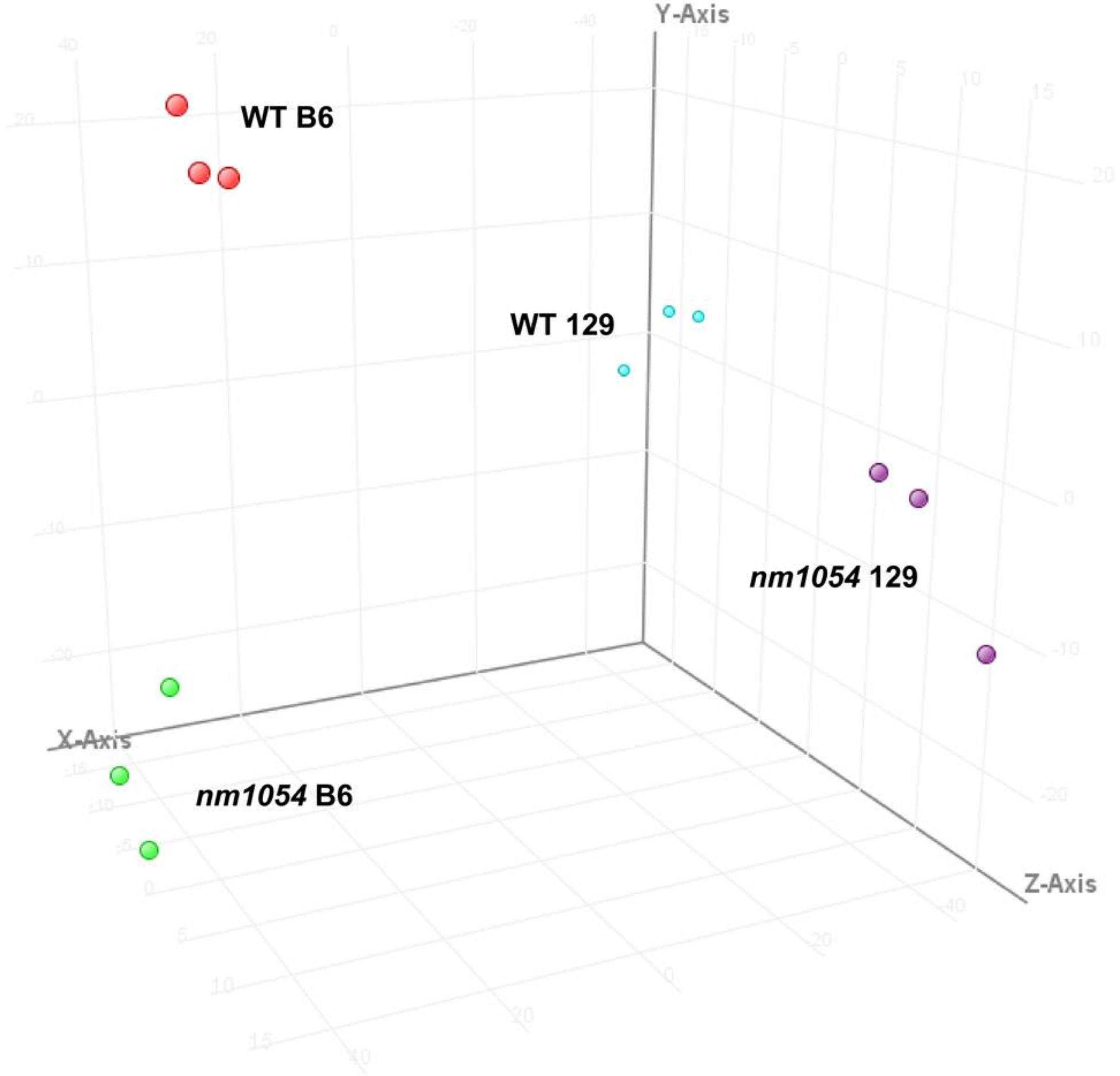

Supplementary Figure S2. Principal component analysis (PCA) plot shows distinction between the sample groups. The individual samples, when plotted in a three dimensional volumetric space (Axes: X – PC1, 75.47%; Y – PC2, 9.12%; Z – PC3, 4.04%), fall consistently into the four distinct sample groups of WT B6, *nm1054* B6, WT 129, and *nm1054* 129.

Supplementary Table S1. Differentially expressed genes between WT 129 and WT B6.

Probe ID	Gene Name	Gene Description	Fold Change*	Log FC	p (Corr)	Entrez Gene ID	Accession Number	RefSeq ID
ILMN_2825109	Zfp330	zinc finger protein 330 (Zfp330), mRNA.	-46.09	-5.53	0.00202	30932	NM_145600.1	NM_145600.1
ILMN_1242107	Cox7a2l	cytochrome c oxidase subunit VIIa polypeptide 2-like	-34.16	-5.09	0.00171		XM_123188.1	XM_123188.1
ILMN_2895177	Epm2aip1	EPM2A (laforin) interacting protein 1 (Epm2aip1), mRNA.+E50D559D6:E48D6:D6:E531	-30.17	-4.91	0.00242	77781	NM_175266.2	NM_175266.2
ILMN_1218868	Cops8	COP9 (constitutive photomorphogenic) homolog, subunit 8 (Arabidopsisthaliana) (Cops8), mRNA.	-24.84	-4.63	0.00223	108679	NM_133805.3	NM_133805.3
ILMN_3009910	Rbm13	RNA binding motif protein 13 (Rbm13), mRNA.	-18.16	-4.18	0.004058187	67920	NM_026453.1	NM_026453.1
ILMN_2768053	Supt16h	suppressor of Ty 16 homolog (S. cerevisiae) (Supt16h), mRNA.	-16.70	-4.06	0.007164895	114741	NM_033618.3	NM_033618.3
ILMN_2746483	Wdr82	WD repeat domain containing 82 (Wdr82), mRNA.	-15.53	-3.96	0.00242	77305	NM_029896.1	NM_029896.1
ILMN_1242829	Prdx2	peroxiredoxin 2 (Prdx2), mRNA.	-15.10	-3.92	0.006564271	21672	NM_011563.2	NM_011563.2
ILMN_2514377	CXADR	coxsackie virus and adenovirus receptor	-14.94	-3.90	0.002020123		NM_009988	
ILMN_3161626	Prkag2	protein kinase, AMP-activated, gamma 2 non-catalytic subunit (Prkag2)	-14.89	-3.90	0.00340	108099	NM_145401.1	NM_145401.1
ILMN_1221102	Arl5a	ADP-ribosylation factor-like 5A (Arl5a), mRNA.	-13.82	-3.79	0.006876021	75423	NM_182994.2	NM_182994.2
ILMN_2730005	Rpl29	ribosomal protein L29 (Rpl29), mRNA.	-13.17	-3.72	0.00333	19944	NM_009082.2	NM_009082.2
ILMN_2776922	GLRX	glutaredoxin	-12.65	-3.66	0.002417001		NM_053108	
ILMN_2507182	Tomm22	translocase of outer mitochondrial membrane 22 homolog, nuclear gene encoding mitochondrial protein	-12.18	-3.61	0.00266	223696	NM_172609.3	NM_172609.3
ILMN_2455850	Gpr137b-ps	G protein-coupled receptor 137B, pseudogene (Gpr137b-ps), non-coding	-11.86	-3.57	0.00243	664862	NR_003568.1	NR_003568.1
ILMN_2678019	Psmd8	proteasome (prosome, macropain) 26S subunit, non-ATPase, 8 (Psmd8)	-11.65	-3.54	0.00688	57296	NM_026545.2	NM_026545.2
ILMN_2658392	Atp2c1	ATPase, Ca++-sequestering (Atp2c1), mRNA.	-11.29	-3.50	0.007164895	235574	NM_175025.2	NM_175025.2
ILMN_1256161	MYT1L	myelin transcription factor 1-like	-10.87	-3.44	0.01978		AK012660	
ILMN_2774882	NECAP2	NECAP endocytosis associated 2	-10.79	-3.43	0.002976273		NM_025383	
ILMN_1219583	Capn2	calpain 2 (Capn2), mRNA.	-10.56	-3.40	0.003850895	12334	NM_009794.1	NM_009794.1
ILMN_2691261	Ndufb10	NADH dehydrogenase (ubiquinone) 1 beta subcomplex, 10	-10.55	-3.40	0.01615		XM_128594.4	XM_128594.4
ILMN_2484987	Man2b1	mannosidase 2, alpha B1 (Man2b1), mRNA.	-10.55	-3.40	0.00275	17159	NM_010764.2	NM_010764.2
ILMN_2733542	LOC100044862	similar to Fbxl3 protein (LOC100044862), mRNA.	-9.86	-3.30	0.00383	100044862	XM_001473206.1	XM_001473206.1
ILMN_2966034	Zfp365	zinc finger protein 365 (Zfp365), mRNA.	-9.32	-3.22	0.00775	216049	NM_178679.2	NM_178679.2
ILMN_2700233	Ccng2	cyclin G2 (Ccng2), mRNA.	-8.83	-3.14	0.01685	12452	NM_007635.3	NM_007635.3
ILMN_1257724	Adamts9	a disintegrin-like and metallopeptidase (reprolysin type) with thrombospondin type 1 motif, 9	-8.78	-3.13	0.007164895			
ILMN_2615468	Fgfr1op2	FGFR1 oncogene partner 2 (Fgfr1op2), mRNA.	-8.59	-3.10	0.010128534	67529	NM_026218.2	NM_026218.2
ILMN_2425376	Cttnbp2	cortactin binding protein 2, transcript variant 5 (Cttnbp2), mRNA.	-8.46	-3.08	0.00912	30785	XM_987526.1	XM_987526.1
ILMN_3160292	Akr1c19	aldo-keto reductase family 1, member C19 (Akr1c19), mRNA.	-8.27	-3.05	0.006876021	432720	NM_001013785.2	NM_001013785.2
ILMN_1255462	Hbb-b1/Hbb-bs	hemoglobin, beta adult major chain/hemoglobin, beta adult s chain	-8.17	-3.03	0.02859		AK011053	
ILMN_1245850	4933427D14Rik	RIKEN cDNA 4933427D14 gene (4933427D14Rik), mRNA.	-8.03	-3.01	0.007754985	74477	NM_028963.2	NM_028963.2
ILMN_1250335	Dfna5	Gsdme, gasdermin E	-7.99	-3.00	0.006992973		NM_008016.1	NM_008016.1
ILMN_2769656	Picalm	phosphatidylinositol binding clathrin assembly protein	-7.87	-2.98	0.005791558		NM_146194	
ILMN_2690232	BC068157	cDNA sequence BC068157 (BC068157), mRNA.	-7.47	-2.90	0.01236	73072	NM_207203.1	NM_207203.1
ILMN_1253773	Nudt6	nudix (nucleoside diphosphate linked moiety X)-type motif 6 (Nudt6),	-7.19	-2.85	0.00990	229228	NM_153561.2	NM_153561.2
ILMN_2696182	Actl6b	actin-like 6B (Actl6b), mRNA.	-7.12	-2.83	0.00333	83766	NM_031404.4	NM_031404.4
ILMN_1238801	Arl3	ADP-ribosylation factor-like 3 (Arl3), mRNA.	-7.10	-2.83	0.00184	56350	NM_019718.2	NM_019718.2
ILMN_2737296	Lars2	leucyl-tRNA synthetase, mitochondrial (Lars2), nuclear gene encoding mitochondrial protein	-6.96	-2.80	0.002417001	102436	NM_153168.2	NM_153168.2
ILMN_1249654	Clasrp	CLK4-associating serine/arginine rich protein	-6.91	-2.79	0.00823		NM_016680	
ILMN_2666279	Arrdc3	arrestin domain containing 3	-6.89	-2.79	0.006876021		NM_178917.2	NM_178917.2
ILMN_1255287	Mela	melanoma antigen	-6.76	-2.76	0.00385		NM_008581	
ILMN_2449449	Zfp68	zinc finger protein 68 (Zfp68), transcript variant 1, mRNA.	-6.52	-2.71	0.011701967	24135	NM_013844.2	NM_013844.2
ILMN_1251839	Myo7a	myosin VIIa (Myo7a), mRNA.	-6.37	-2.67	0.00264	17921	NM_008663.2	NM_008663.2
ILMN_1223734	Atf4	activating transcription factor 4 (Atf4), mRNA.	-6.36	-2.67	0.006876021	11911	NM_009716.2	NM_009716.2
ILMN_2893063	Rnf219	ring finger protein 219 (Rnf219), mRNA.	-6.22	-2.64	0.01285	72486	NM_026047.4	NM_026047.4
ILMN_1218118	Tia1	cytotoxic granule-associated RNA binding protein 1	-6.19	-2.63	0.01594		AK009502	
ILMN_1220270	Mef2c	myocyte enhancer factor 2C	-6.02	-2.59	0.01615		AK047994	
ILMN_2420119	MTDH	metadherin	-6.01	-2.59	0.003334355		NM_026002	
ILMN_2704027	Slc25a18	solute carrier family 25 (mitochondrial carrier), member 18 (Slc25a18)	-5.99	-2.58	0.01600	71803	NM_001081048.1	NM_001081048.1
ILMN_2775050	Arv1	ARV1 homolog (yeast) (Arv1), mRNA.	-5.79	-2.53	0.00202	68865	NM_026855.3	NM_026855.3
ILMN_2892292	Alg9	asparagine-linked glycosylation 9 homolog (yeast, alpha 1,2 mannosyltransferase) (Alg9)	-5.79	-2.53	0.00345	102580	NM_133981.1	NM_133981.1
ILMN_1243212	Sparc	secreted acidic cysteine rich glycoprotein (Sparc), mRNA.	-5.74	-2.52	0.01800	20692	NM_009242.3	NM_009242.3
ILMN_2585137	HMGCS1	3-hydroxy-3-methylglutaryl-Coenzyme A synthase 1	-5.70	-2.51	0.01236		AK078743	
ILMN_2942353	Ccl25	chemokine (C-C motif) ligand 25 (Ccl25), mRNA.	-5.67	-2.50	0.01798	20300	NM_009138.1	NM_009138.1
ILMN_1247404	B230312I18Rik	RIKEN cDNA B230312I18 gene, transcript variant 3 (B230312I18Rik), mR	-5.62	-2.49	0.007754985	233058	XM_001002154.1	XM_001002154.1
ILMN_1219686	Esd	esterase D/formylglutathione hydrolase (Esd), mRNA.	-5.50	-2.46	0.00776	13885	NM_016903.4	NM_016903.4
ILMN_2703061	2810408P10Rik	RIKEN cDNA 2810408P10 gene (2810408P10Rik), mRNA.	-5.49	-2.46	0.00243	242747	NM_198619.2	NM_198619.2
ILMN_3150536	1200015F23Rik	RIKEN cDNA 1200015F23 gene (1200015F23Rik), mRNA. XM_924991	-5.49	-2.46	0.00242	67809	NM_001033136.2	NM_001033136.2
ILMN_2687140	B3galt6	UDP-Gal:betaGal beta 1,3-galactosyltransferase, polypeptide 6 (B3galt6)	-5.48	-2.45	0.002417001	117592	NM_080445.4	NM_080445.4
ILMN_1214850	Pak1	p21 (CDKN1A)-activated kinase 1 (Pak1), mRNA.	-5.31	-2.41	0.018368704	18479	NM_011035.2	NM_011035.2
ILMN_2543507	1190007I07Rik	RIKEN cDNA 1190007I07 gene (1190007I07Rik), mRNA.	-5.06	-2.34	0.03328955	544717	XM_985039.1	XM_985039.1
ILMN_1255006	Rpe	ribulose-5-phosphate-3-epimerase (Rpe), mRNA.	-5.05	-2.34	0.01175	66646	NM_025683.2	NM_025683.2
ILMN_1249638	Rbbp4	retinoblastoma binding protein 4 (Rbbp4), mRNA.	-4.94	-2.30	0.00668	19646	NM_009030.3	NM_009030.3
ILMN_2502542	Uap1	UDP-N-acetylglucosamine pyrophosphorylase 1 (Uap1), mRNA.	-4.92	-2.30	0.029329881	107652	NM_133806.4	NM_133806.4
ILMN_3049465	D830030K20Rik	RIKEN cDNA D830030K20 gene (D830030K20Rik), mRNA.	-4.58	-2.20	0.01242	320333	NM_177135.2	NM_177135.2

Supplementary Table S1. Continued…

ILMN_2604226	Sema5a	sema domain, seven thrombospondin repeats (type 1 and type 1-like), (semaphorin) 5A (Sema5a)	-4.53	-2.18	0.00345	20356	NM_009154.2	NM_009154.2
ILMN_2701304	Ube2cbp	ubiquitin-conjugating enzyme E2C binding protein (Ube2cbp), mRNA.	-4.42	-2.14	0.001839255	70348	NM_027394.2	NM_027394.2
ILMN_1241137	Taf6	TAF6 RNA polymerase II, TATA box binding protein (TBP)-associated factor (Taf6)	-4.40	-2.14	0.02911	21343	NM_009315.3	NM_009315.3
ILMN_1233531	2610528E23Rik	RIKEN cDNA 2610528E23 gene (2610528E23Rik), mRNA.	-4.38	-2.13	0.03774	66497	NM_025599.2	NM_025599.2
ILMN_1255422	Ccrn4l	CCR4 carbon catabolite repression 4-like (S. cerevisiae) (Ccrn4l), m	-4.34	-2.12	0.00716	12457	NM_009834.1	NM_009834.1
ILMN_1214511	Ern2	endoplasmic reticulum (ER) to nucleus signalling 2 (Ern2), mRNA.	-4.33	-2.11	0.025176654	26918	NM_012016.2	NM_012016.2
ILMN_1222402	Pla2g4b	phospholipase A2, group IVB (cytosolic	-4.28	-2.10	0.01626		XM_358347.1	XM_358347.1
ILMN_1219670	Gpatch4	G patch domain containing 4 (Gpatch4), mRNA.	-4.27	-2.09	0.019147642	66614	NM_025663.2	NM_025663.2
ILMN_2998738	EG381438	predicted gene, EG381438 (EG381438), mRNA.	-4.25	-2.09	0.01408	381438	NM_198657.1	NM_198657.1
ILMN_2518483	Pla2g12a	phospholipase A2, group XIIA	-4.13	-2.05	0.00298		NM_023196	
ILMN_1221526	LOC100041516	similar to 4933409K07Rik protein (LOC100041516), misc RNA.	-4.12	-2.04	0.027745096	100041516	XR_031127.1	XR_031127.1
ILMN_1216231	Scoc	short coiled-coil protein (Scoc), transcript variant 1, mRNA.	-4.10	-2.04	0.00668	56367	NM_001039137.2	NM_001039137.2
ILMN_2640848	Ccl21c	chemokine (C-C motif) ligand 21C (leucine)	-4.04	-2.01	0.01242		NM_023052	
ILMN_2728516	Psmc4	proteasome (prosome, macropain) 26S subunit, ATPase, 4	-4.00	-2.00	0.03580043		XM_355872.1	XM_355872.1
ILMN_2814350	Mkks	McKusick-Kaufman syndrome protein (Mkks), mRNA.	-3.96	-1.98	0.011610471	59030	NM_021527.1	NM_021527.1
ILMN_1228242	Zfp383	zinc finger protein 383 (Zfp383), mRNA.	-3.92	-1.97	0.009697126	73729	XM_001003466.1	XM_001003466.1
ILMN_1214375	Gpn2	GPN-loop GTPase 2 (Gpn2), mRNA.	-3.89	-1.96	0.01600	100210	NM_133884.1	NM_133884.1
ILMN_1232668	MXD1	MAX dimerization protein 1	-3.88	-1.96	0.017523285		NM_010751	
ILMN_2441501	Clstn1	calsyntenin 1	-3.83	-1.94	0.00406		NM_023051	
ILMN_2510187	Slc39a2	solute carrier family 39 (zinc transporter), member 2, transcript variant 2 (Slc39a2)	-3.83	-1.94	0.01170	214922	XM_989872.1	XM_989872.1
ILMN_3133817	Arhgap12	Rho GTPase activating protein 12 (Arhgap12), transcript variant 2, m	-3.80	-1.92	0.02933	75415	NM_029277.2	NM_029277.2
ILMN_2764047	Hmgcll1	3-hydroxymethyl-3-methylglutaryl-Coenzyme A lyase-like 1 (Hmgcll1),	-3.76	-1.91	0.01077	208982	NM_173731.2	NM_173731.2
ILMN_2800380	Trim9	tripartite motif protein 9 (Trim9), mRNA.	-3.70	-1.89	0.02600	94090	NM_053167.1	NM_053167.1
ILMN_1234412	LOC674427	similar to ribosomal protein L7a (LOC674427), misc RNA.	-3.68	-1.88	0.01187	674427	XR_030796.1	XR_030796.1
ILMN_2599861	Defb11	defensin beta 11 (Defb11), mRNA.	-3.61	-1.85	0.02672	246081	NM_139221.1	NM_139221.1
ILMN_1255053	Plk1	polo-like kinase 1 (Drosophila) (Plk1), mRNA.	-3.58	-1.84	0.01923	18817	NM_011121.3	NM_011121.3
ILMN_1214139	Fmn2	formin 2	-3.54	-1.82	0.01102		AK013585	
ILMN_2890496	Rbbp9	retinoblastoma binding protein 9 (Rbbp9), mRNA.	-3.50	-1.81	0.01236	26450	NM_015754.2	NM_015754.2
ILMN_1240178	Ccndbp1	cyclin D-type binding-protein 1 (Ccndbp1), mRNA.	-3.47	-1.80	0.002417001	17151	NM_010761.2	NM_010761.2
ILMN_2722129	Hps1	Hermansky-Pudlak syndrome 1 homolog (human) (Hps1), mRNA.	-3.42	-1.77	0.009116249	192236	NM_019424.2	NM_019424.2
ILMN_1226111	LOC100043821	hypothetical protein LOC100043821 (LOC100043821), mRNA.	-3.35	-1.74	0.005791558	100043821	XM_001481017.1	XM_001481017.1
ILMN_1248465	Zcchc3	zinc finger, CCHC domain containing 3 (Zcchc3), mRNA.	-3.34	-1.74	0.02396	67917	NM_175126.3	NM_175126.3
ILMN_2657728	Stxbp2	syntaxin binding protein 2	-3.30	-1.72	0.003448847		NM_011503.2	NM_011503.2
ILMN_2756023	Pcsk9	proprotein convertase subtilisin/kexin type 9 (Pcsk9), mRNA.	-3.24	-1.70	0.014517168	100102	NM_153565.1	NM_153565.1
ILMN_1233812	CCNY	cyclin Y	-3.24	-1.69	0.02036		AK014201	
ILMN_1214422	2810408P10Rik	RIKEN cDNA 2810408P10 gene (2810408P10Rik), mRNA.	-3.12	-1.64	0.01958	242747	NM_198619.2	NM_198619.2
ILMN_2603081	Nt5c3	5'-nucleotidase, cytosolic III (Nt5c3), mRNA.	-3.08	-1.62	0.00223	107569	NM_026004.2	NM_026004.2
ILMN_2717621	Rps15a	ribosomal protein S15a (Rps15a), mRNA.	-3.06	-1.61	0.012976651	267019	NM_170669.2	NM_170669.2
ILMN_2778076	CDPF1	cysteine rich, DPF motif domain containing 1	-3.06	-1.61	0.04228		NM_197998	
ILMN_2717176	Rgl1	ral guanine nucleotide dissociation stimulator,-like 1 (Rgl1), mRNA.	-3.05	-1.61	0.00862	19731	NM_016846.3	NM_016846.3
ILMN_2826826	Rtbdn	retbindin (Rtbdn), mRNA.	-3.05	-1.61	0.03580043	234542	NM_144929.2	NM_144929.2
ILMN_1260038	Eif4e	eukaryotic translation initiation factor 4E (Eif4e), mRNA.	-3.03	-1.60	0.044227295	13684	NM_007917.3	NM_007917.3
ILMN_1259641	LOC674912	similar to melanoma antigen, transcript variant 1 (LOC674912)	-3.02	-1.60	0.02677	674912	XM_979860.1	XM_979860.1
ILMN_2690256	Insig2	insulin induced gene 2 (Insig2), mRNA.	-3.01	-1.59	0.006876021	72999	NM_133748.1	NM_133748.1
ILMN_1245711	C030034I22Rik	RIKEN cDNA C030034I22 gene (C030034I22Rik), mRNA.	-3.01	-1.59	0.01236	77533	XM_001478807.1	XM_001478807.1
ILMN_2747480	Srpr	signal recognition particle receptor ('docking protein') (Srpr)	-2.99	-1.58	0.01015	67398	NM_026130.1	NM_026130.1
ILMN_1255262	GPATCH4	G patch domain containing 4	-2.99	-1.58	0.02607		AK018306	
ILMN_2588199	Mrps27	mitochondrial ribosomal protein S27 (Mrps27), nuclear gene encoding mitochondrial protein	-2.99	-1.58	0.009697126	218506	NM_173757.3	NM_173757.3
ILMN_1236666	Acsl6	acyl-CoA synthetase long-chain family member 6 (Acsl6), transcript variant 4	-2.96	-1.57	0.009116249	216739	NM_001033599.1	NM_001033599.1
ILMN_1245420	ZMYM6	zinc finger, MYM-type 6	-2.96	-1.57	0.01138		AK035122	
ILMN_3006575	6330503K22Rik	RIKEN cDNA 6330503K22 gene (6330503K22Rik), mRNA.	-2.95	-1.56	0.01236	101565	NM_182995.1	NM_182995.1
ILMN_1250939	Agpat5	1-acylglycerol-3-phosphate O-acyltransferase 5 (lysophosphatidic acid acyltransferase, epsilon) (Agpat5)	-2.92	-1.55	0.019801201	52123	NM_026792.3	NM_026792.3
ILMN_1223045	2810417H13Rik	RIKEN cDNA 2810417H13 gene (2810417H13Rik), mRNA.	-2.90	-1.53	0.03371	68026	NM_026515.2	NM_026515.2
ILMN_2693858	D14Ertd449e	DNA segment, Chr 14, ERATO Doi 449, expressed (D14Ertd449e), mRNA.	-2.89	-1.53	0.00184	66039	NM_025311.1	NM_025311.1
ILMN_1251041	Btrc	beta-transducin repeat containing protein (Btrc), transcript variant 1	-2.88	-1.53	0.016151201	12234	NM_001037758.1	NM_001037758.1
ILMN_2723346	1700029J07Rik	RIKEN cDNA 1700029J07 gene (1700029J07Rik), mRNA.	-2.87	-1.52	0.019801201	69479	NM_001033148.2	NM_001033148.2
ILMN_2733330	Rps3a	ribosomal protein S3a (Rps3a), mRNA.	-2.87	-1.52	0.042064697	20091	NM_016959.2	NM_016959.2
ILMN_2598576	Parp2	poly (ADP-ribose) polymerase family, member 2 (Parp2), mRNA.	-2.87	-1.52	0.006876021	11546	NM_009632.2	NM_009632.2
ILMN_1252992	AFG3L2	AFG3-like AAA ATPase 2	-2.81	-1.49	0.04165		AK080118	
ILMN_2860964	Med23	mediator complex subunit 23 (Med23), mRNA.	-2.81	-1.49	0.022358822	70208	NM_027347.2	NM_027347.2
ILMN_2865527	Krt12	keratin 12 (Krt12), mRNA.	-2.80	-1.49	0.01851	268482	NM_010661.2	NM_010661.2
ILMN_1223546	6430571L13Rik	RIKEN cDNA 6430571L13 gene (6430571L13Rik), mRNA.	-2.79	-1.48	0.016151201	235599	NM_175486.3	NM_175486.3
ILMN_1219305	Gstm6	glutathione S-transferase, mu 6 (Gstm6), mRNA.	-2.78	-1.48	0.04113	14867	NM_008184.3	NM_008184.3
ILMN_2660803	Bckdhb	branched chain ketoacid dehydrogenase E1, beta polypeptide, nuclear gene encoding mitochondrial protein	-2.78	-1.47	0.03421	12040	NM_199195.1	NM_199195.1
ILMN_3011719	6430706D22Rik	RIKEN cDNA 6430706D22 gene (6430706D22Rik), mRNA.	-2.77	-1.47	0.01915	381280	NM_198652.1	NM_198652.1
ILMN_2482572	Flnb	filamin, beta (Flnb), mRNA. XM_904364 XM_990154	-2.76	-1.46	0.04485	286940	NM_134080.1	NM_134080.1
ILMN_2598402	Parp8	poly (ADP-ribose) polymerase family, member 8 (Parp8), mRNA.	-2.74	-1.46	0.01062	52552	NM_001081009.1	NM_001081009.1
ILMN_2600315	Ercc8	excision repaiross-complementing rodent repair deficiency, complementation group 8 (Ercc8)	-2.73	-1.45	0.01285	71991	NM_028042.3	NM_028042.3
ILMN_2602151	Rpap1	RNA polymerase II associated protein 1 (Rpap1), mRNA.	-2.71	-1.44	0.04359	68925	NM_177294.4	NM_177294.4
ILMN_1232989	Ehd3	EH-domain containing 3 (Ehd3), mRNA.	-2.71	-1.44	0.027312322	57440	NM_020578.2	NM_020578.2
ILMN_2507331	Xylt1	xylosyltransferase 1 (Xylt1), mRNA.	-2.68	-1.42	0.03681	233781	NM_175645.3	NM_175645.3

Supplementary Table S1. Continued…

ILMN_2654906	Mgat3	mannoside acetylglucosaminyltransferase 3 (Mgat3), mRNA.	-2.67	-1.41	0.030583858	17309	NM_010795.3	NM_010795.3
ILMN_2970834	Pex19	peroxisome biogenesis factor 19 (Pex19), mRNA.	-2.66	-1.41	0.018162947	19298	NM_023041.2	NM_023041.2
ILMN_2475271	Trappc5	trafficking protein particle complex 5 (Trappc5), mRNA.	-2.65	-1.41	0.041133914	66682	NM_025701.3	NM_025701.3
ILMN_3160771	Cd200	CD200 antigen	-2.64	-1.40	0.04206		NM_010818	
ILMN_2687011	Krt1-12	keratin 12	-2.62	-1.39	0.02799		NM_010661.1	NM_010661.1
ILMN_2747754	Pygb	brain glycogen phosphorylase (Pygb), mRNA.	-2.61	-1.38	0.010963273	110078	NM_153781.1	NM_153781.1
ILMN_1234100	Bsdc1	BSD domain containing 1 (Bsdc1), mRNA.	-2.60	-1.38	0.00711	100383	NM_133889.2	NM_133889.2
ILMN_1213374	KCNH7	potassium voltage-gated channel, subfamily H (eag-related), member 7	-2.59	-1.37	0.00912		AK052366	
ILMN_2867013	Zfp160	zinc finger protein 160 (Zfp160), mRNA.	-2.58	-1.37	0.02721	224585	NM_145483.1	NM_145483.1
ILMN_1255657	Yipf4	Yip1 domain family, member 4 (Yipf4), mRNA.	-2.53	-1.34	0.03421	67864	NM_026417.3	NM_026417.3
ILMN_1246132	E230029C05Rik	RIKEN cDNA E230029C05 gene (E230029C05Rik), mRNA.	-2.51	-1.33	0.03774	319711	XM_001473432.1	XM_001473432.1
ILMN_2686353	Sult2b1	sulfotransferase family, cytosolic, 2B, member 1 (Sult2b1), mRNA.	-2.51	-1.32	0.03863	54200	NM_017465.1	NM_017465.1
ILMN_1250569	Rapgefl1	Rap guanine nucleotide exchange factor (GEF)-like 1 (Rapgefl1), mRNA	-2.50	-1.32	0.02341	268480	NM_001080925.1	NM_001080925.1
ILMN_2628567	Phlda3	pleckstrin homology-like domain, family A, member 3 (Phlda3), mRNA.	-2.49	-1.32	0.00693	27280	NM_013750.1	NM_013750.1
ILMN_2613469	Psmb5	proteasome (prosome, macropain) subunit, beta type 5 (Psmb5), mRNA.	-2.49	-1.31	0.03880	19173	NM_011186.1	NM_011186.1
ILMN_1258526	Lgals3bp	lectin, galactoside-binding, soluble, 3 binding protein (Lgals3bp),	-2.48	-1.31	0.024340035	19039	NM_011150.2	NM_011150.2
ILMN_2503166	C330011F01Rik	RIKEN cDNA C330011F01 gene (C330011F01Rik), mRNA.	-2.48	-1.31	0.04254	78605	XM_001005388.1	XM_001005388.1
ILMN_2944843	LOC545056	ubiquitin-conjugating enzyme E2, J2 homolog pseudogene (LOC545056) on chromosome 14.	-2.47	-1.30	0.01096	545056	NR_002889.1	NR_002889.1
ILMN_2581972	E2F6	E2F transcription factor 6	-2.45	-1.29	0.009697126		AK075684	
ILMN_1221341	LOC100047226	hypothetical protein LOC100047226 (LOC100047226), misc RNA.	-2.44	-1.29	0.03648	100047226	XR_033502.1	XR_033502.1
ILMN_2457372	TRUB1	TruB pseudouridine (psi) synthase family member 1	-2.44	-1.28	0.03371		NM_028115	
ILMN_1238215	CTGF	connective tissue growth factor	-2.41	-1.27	0.01615		NM_010217	
ILMN_1236107	Ogfrl1	opioid growth factor receptor-like 1 (Ogfrl1), mRNA.	-2.40	-1.26	0.02456886	70155	XM_973033.1	XM_973033.1
ILMN_3158444	Ank2	ankyrin 2, brain (Ank2), transcript variant 2, mRNA.	-2.40	-1.26	0.04867	109676	NM_178655.3	NM_178655.3
ILMN_3135697	1200003I07Rik	RIKEN cDNA 1200003I07 gene (1200003I07Rik), transcript variant 3, mR	-2.39	-1.26	0.021878595	66869	NM_181274.3	NM_181274.3
ILMN_2637484	Nanos2	nanos homolog 2 (Drosophila) (Nanos2), mRNA.	-2.38	-1.25	0.03449	378430	NM_194064.2	NM_194064.2
ILMN_1257622	Tubgcp4	tubulin, gamma complex associated protein 4 (Tubgcp4), mRNA.	-2.36	-1.24	0.02623	51885	NM_153387.2	NM_153387.2
ILMN_1225461	PEX6	peroxisomal biogenesis factor 6	-2.33	-1.22	0.02222		AK030565	
ILMN_1244343	SUN2	Sad1 and UNC84 domain containing 2	-2.31	-1.21	0.02780		NM_194342	
ILMN_2886468	Fbxo46	F-box protein 46 (Fbxo46), mRNA.	-2.31	-1.21	0.012356669	243867	NM_175530.2	NM_175530.2
ILMN_2498787	Zfp236	zinc finger protein 236, transcript variant 1 (Zfp236), mRNA.	-2.31	-1.21	0.018842775	329002	XM_484752.5	XM_484752.5
ILMN_3151840	Ppm1m	protein phosphatase 1M (Ppm1m), transcript variant 1, mRNA.	-2.31	-1.21	0.010963273	67905	NM_026447.2	NM_026447.2
ILMN_2777722	KDM5B	lysine (K)-specific demethylase 5B	-2.30	-1.20	0.01578		NM_152895	
ILMN_1224437	PPM1M	protein phosphatase 1M	-2.29	-1.20	0.007164895		NM_026447	
ILMN_2739816	AW822252	expressed sequence AW822252 (AW822252), mRNA.	-2.29	-1.20	0.019147642	331578	XM_001474711.1	XM_001474711.1
ILMN_1213448	LOC669658	similar to melanoma antigen (LOC669658), mRNA.	-2.29	-1.20	0.037029903	669658	XM_976371.1	XM_976371.1
ILMN_1216136	Cbfa2t3h	core-binding factor, runt domain, alpha subunit 2, translocated to, 3 homolog (human) (Cbfa2t3h)	-2.29	-1.19	0.00656	12398	NM_009824.1	NM_009824.1
ILMN_2819558	Bach2	BTB and CNC homology 2 (Bach2), mRNA.	-2.28	-1.19	0.02072362	12014	NM_007521.2	NM_007521.2
ILMN_2903698	Snapc3	small nuclear RNA activating complex, polypeptide 3 (Snapc3), mRNA.	-2.28	-1.19	0.006992973	77634	NM_029949.1	NM_029949.1
ILMN_1253848	Neo1	neogenin	-2.26	-1.17	0.04165		AK077830	
ILMN_2776952	Tmem55b	transmembrane protein 55b (Tmem55b), mRNA. XM_919952 XM_919965	-2.25	-1.17	0.01649	219024	NM_001033271.3	NM_001033271.3
ILMN_1221985	1700123A16Rik	RIKEN cDNA 1700123A16 gene (1700123A16Rik), mRNA.	-2.25	-1.17	0.016258812	73610	XM_984400.1	XM_984400.1
ILMN_2602257	Tmem68	transmembrane protein 68 (Tmem68), mRNA.	-2.25	-1.17	0.021965643	72098	NM_028097.3	NM_028097.3
ILMN_2636403	Axud1	AXIN1 up-regulated 1 (Axud1), mRNA.	-2.24	-1.17	0.02869	215418	NM_153287.3	NM_153287.3
ILMN_2773909	1600014C10Rik	RIKEN cDNA 1600014C10 gene (1600014C10Rik), transcript variant 2, mR	-2.22	-1.15	0.010963273	72244	NM_028166.3	NM_028166.3
ILMN_1224540	Eltd1	EGF, latrophilin seven transmembrane domain containing 1 (Eltd1), mR	-2.20	-1.14	0.03649	170757	NM_133222.2	NM_133222.2
ILMN_2714415	4922501C03Rik	RIKEN cDNA 4922501C03 gene (4922501C03Rik), mRNA.	-2.20	-1.14	0.04202	382090	NM_199316.2	NM_199316.2
ILMN_1253316	Polr3f	polymerase (RNA) III (DNA directed) polypeptide F	-2.19	-1.13	0.03421134		NM_027417	
ILMN_1254717	Zfp429	zinc finger protein 429 (Zfp429), mRNA.	-2.18	-1.13	0.00937	72807	NM_001080941.1	NM_001080941.1
ILMN_1221822	Rbm12	RNA binding motif protein 12 (Rbm12), transcript variant 2, mRNA.	-2.18	-1.12	0.02577	75710	NM_170598.2	NM_170598.2
ILMN_1229343	Mboat2	membrane bound O-acyltransferase domain containing 2 (Mboat2), transcript variant 2	-2.18	-1.12	0.039169736	67216	NM_001083341.1	NM_001083341.1
ILMN_1255776	BC017643	cDNA sequence BC017643 (BC017643), mRNA.	-2.17	-1.11	0.030094454	217370	NM_144832.1	NM_144832.1
ILMN_2747430	Atp7a	ATPase, Cu++ transporting, alpha polypeptide (Atp7a), mRNA.	-2.16	-1.11	0.04193	11977	NM_009726.3	NM_009726.3
ILMN_2920541	Tbc1d9	TBC1 domain family, member 9 (Tbc1d9), mRNA.	-2.15	-1.10	0.032536317	71310	NM_027758.1	NM_027758.1
ILMN_1236868	Idb2	Id2, inhibitor of DNA binding 2	-2.13	-1.09	0.00688		AK013239	
ILMN_2654651	Thbs3	thrombospondin 3 (Thbs3), mRNA.	-2.13	-1.09	0.03864	21827	NM_013691.1	NM_013691.1
ILMN_2968211	Lgals4	lectin, galactose binding, soluble 4 (Lgals4), mRNA.	-2.12	-1.09	0.036344867	16855	NM_010706.1	NM_010706.1
ILMN_1217054	Ckmt1	creatine kinase, mitochondrial 1, ubiquitous	-2.12	-1.08	0.04381		AK018487	
ILMN_2497745	Mtap9	microtubule-associated protein 9 (Mtap9), mRNA.	-2.12	-1.08	0.01096	213582	NM_001081230.1	NM_001081230.1
ILMN_2571616	TPM4	tropomyosin 4	-2.11	-1.08	0.03880		AK049383	
ILMN_1230963	dguok	deoxyguanosine kinase	-2.10	-1.07	0.00656		AK003639	
ILMN_2435505	Usf1	upstream transcription factor 1 (Usf1), mRNA.	-2.10	-1.07	0.04167	22278	NM_009480.2	NM_009480.2
ILMN_1225370	Wdr92	WD repeat domain 92 (Wdr92), mRNA.	-2.09	-1.07	0.02486	103784	NM_178909.4	NM_178909.4
ILMN_2606711	LOC100046163	similar to Nme6 protein (LOC100046163), mRNA.	-2.09	-1.06	0.02139	100046163	XM_001475710.1	XM_001475710.1
ILMN_1217776	Mll5	Kmt2e, lysine (K)-specific methyltransferase 2E	-2.09	-1.06	0.03449		XM_485570	
ILMN_2862706	H2-Q5	histocompatibility 2, Q region locus 5 (H2-Q5), mRNA.	-2.08	-1.06	0.04842014	15016	NM_010393.1	NM_010393.1
ILMN_1228942	Cd59a	CD59a antigen (Cd59a), mRNA.	-2.07	-1.05	0.01151	12509	NM_007652.2	NM_007652.2
ILMN_1257178	Zfp367	zinc finger protein 367 (Zfp367), mRNA.	-2.07	-1.05	0.02504	238673	NM_175494.4	NM_175494.4
ILMN_1253609	EED	embryonic ectoderm development	-2.06	-1.04	0.038795386		AK016228	
ILMN_2645460	2410146L05Rik	RIKEN cDNA 2410146L05 gene (2410146L05Rik), mRNA.	-2.05	-1.04	0.019121023	67968	NM_026480.2	NM_026480.2
ILMN_1216709	PBX4	pre B cell leukemia homeobox 4	-2.04	-1.03	0.03421		NM_030555	

Supplementary Table S1. Continued…

ILMN_1218136	USP53	ubiquitin specific peptidase 53	-2.04	-1.03	0.010963273		AK030317	
ILMN_2459211	Dgkh	diacylglycerol kinase, eta	-2.01	-1.01	0.037781753		XM_484397	
ILMN_2696066	3110070M22Rik	RIKEN cDNA 3110070M22 gene (3110070M22Rik), mRNA.	-2.01	-1.01	0.04722	67304	NM_026084.2	NM_026084.2
ILMN_1227126	Ppp2r3a	protein phosphatase 2 (formerly 2A), regulatory subunit B'', alpha (Ppp2r3a)	-2.00	-1.00	0.04899	19054	XM_001471965.1	XM_001471965.1
ILMN_1233455	Olfml3	olfactomedin-like 3 (Olfml3), mRNA.	-1.99	-0.99	0.04166539	99543	NM_133859.2	NM_133859.2
ILMN_2703182	Lgals7	lectin, galactose binding, soluble 7 (Lgals7), mRNA.	-1.98	-0.98	0.02139	16858	NM_008496.4	NM_008496.4
ILMN_1257623	Pik3ap1	phosphoinositide-3-kinase adaptor protein 1 (Pik3ap1), mRNA.	-1.95	-0.96	0.019778939	83490	NM_031376.2	NM_031376.2
ILMN_2775098	Cyb5	cytochrome b-5 (Cyb5), mRNA.	-1.93	-0.95	0.016469356	109672	NM_025797.3	NM_025797.3
ILMN_3004949	D930028F11Rik	RIKEN cDNA D930028F11 gene (D930028F11Rik), mRNA.	-1.92	-0.94	0.04165212	244853	NM_172921.2	NM_172921.2
ILMN_2676512	Riok1	RIO kinase 1 (yeast) (Riok1), mRNA.	-1.91	-0.94	0.024906604	71340	NM_024242.2	NM_024242.2
ILMN_2519789	Zfp101	zinc finger protein 101 (Zfp101), mRNA.	-1.90	-0.93	0.03482	22643	NM_009542.2	NM_009542.2
ILMN_1258206	Atm	ataxia telangiectasia mutated homolog (human) (Atm), mRNA.	-1.89	-0.92	0.026658215	11920	NM_007499.1	NM_007499.1
ILMN_2667814	Iars2	isoleucine-tRNA synthetase 2, mitochondrial (Iars2), mRNA.	-1.89	-0.92	0.01355	381314	NM_198653.1	NM_198653.1
ILMN_1222767	H2afj	H2A histone family, member J (H2afj), mRNA.	-1.88	-0.91	0.00830	232440	NM_177688.4	NM_177688.4
ILMN_1249466	Fut10	fucosyltransferase 10 (Fut10), transcript variant A, mRNA.	-1.88	-0.91	0.04050	171167	NM_001012517.4	NM_001012517.4
ILMN_2703621	Zfp593	zinc finger protein 593 (Zfp593), mRNA.	-1.87	-0.90	0.02607	68040	NM_024215.2	NM_024215.2
ILMN_2702403	Htr3a	5-hydroxytryptamine (serotonin) receptor 3A (Htr3a), transcript variant 2	-1.86	-0.90	0.03580043	15561	NM_001099644.1	NM_001099644.1
ILMN_2593993	Eif4el3	EIF4E2, eukaryotic translation initiation factor 4E family member 2	-1.86	-0.90	0.01497		NM_023314.2	NM_023314.2
ILMN_2509817	Atp8a1	ATPase, aminophospholipid transporter (APLT), class I, type 8A, member 1 (Atp8a1), transcript variant 2	-1.86	-0.89	0.02577	11980	NM_009727.2	NM_009727.2
ILMN_1257336	Cwf19l1	CWF19-like 1, cell cycle control (S. pombe) (Cwf19l1), mRNA.	-1.86	-0.89	0.016151201	72502	NM_001081077.1	NM_001081077.1
ILMN_1252850	Rbm8a	RNA binding motif protein 8a (Rbm8a), mRNA.	-1.85	-0.89	0.012917056	60365	NM_025875.1	NM_025875.1
ILMN_2668886	6720456H20Rik	RIKEN cDNA 6720456H20 gene (6720456H20Rik), mRNA.	-1.85	-0.89	0.02173	218989	NM_172600.2	NM_172600.2
ILMN_2883164	Serpine2	serine (or cysteine) peptidase inhibitor, clade E, member 2 (Serpine2)	-1.85	-0.88	0.039169736	20720	NM_009255.2	NM_009255.2
ILMN_1250138	Muc1	mucin 1, transmembrane (Muc1), mRNA.	-1.84	-0.88	0.01102	17829	NM_013605.1	NM_013605.1
ILMN_2740869	Rims2	regulating synaptic membrane exocytosis 2 (Rims2), mRNA.	-1.84	-0.88	0.03159	116838	NM_053271.1	NM_053271.1
ILMN_1252144	Mif4gd	MIF4G domain containing (Mif4gd), mRNA.	-1.84	-0.88	0.04423	69674	NM_027162.3	NM_027162.3
ILMN_2621921	Clcn7	chloride channel 7 (Clcn7), mRNA.	-1.83	-0.87	0.005791558	26373	NM_011930.3	NM_011930.3
ILMN_1232341	A730008H23Rik	RIKEN cDNA A730008H23 gene (A730008H23Rik), mRNA.	-1.83	-0.87	0.018325465	212427	NM_172505.1	NM_172505.1
ILMN_1239863	E330016A19Rik	RIKEN cDNA E330016A19 gene (E330016A19Rik), mRNA.	-1.83	-0.87	0.0291027	214763	NM_173386.3	NM_173386.3
ILMN_2683222	Srd5a3	steroid 5 alpha-reductase 3 (Srd5a3), mRNA.	-1.82	-0.87	0.02955	57357	NM_020611.3	NM_020611.3
ILMN_2713898	Ddr1	discoidin domain receptor family, member 1 (Ddr1), transcript variant 1	-1.82	-0.86	0.00716	12305	NM_007584.2	NM_007584.2
ILMN_2768972	Fam107a	family with sequence similarity 107, member A (Fam107a), mRNA.	-1.82	-0.86	0.041925836	268709	NM_183187.3	NM_183187.3
ILMN_2671923	Ly86	lymphocyte antigen 86 (Ly86), mRNA.	-1.82	-0.86	0.04254	17084	NM_010745.1	NM_010745.1
ILMN_2852533	Ahcy	S-adenosylhomocysteine hydrolase (Ahcy), mRNA.	-1.82	-0.86	0.042954423	269378	NM_016661.2	NM_016661.2
ILMN_3060512	Sel1l	sel-1 suppressor of lin-12-like (C. elegans) (Sel1l), transcript variant 1	-1.81	-0.86	0.02911	20338	NM_001039089.1	NM_001039089.1
ILMN_1223832	LOC100047833	similar to MK-5 type 2 (LOC100047833), mRNA.	-1.80	-0.85	0.03031592	100047833	XM_001479242.1	XM_001479242.1
ILMN_1213815	Mtap6	Map6, microtubule-associated protein	-1.80	-0.85	0.02257		AK082709	
ILMN_2840958	Thbs3	thrombospondin 3 (Thbs3), mRNA.	-1.79	-0.84	0.03880	21827	NM_013691.1	NM_013691.1
ILMN_2741872	Heatr5a	HEAT repeat containing 5A (Heatr5a), mRNA.	-1.79	-0.84	0.04909	320487	NM_177171.4	NM_177171.4
ILMN_2925281	Abcd3	ATP-binding cassette, sub-family D (ALD), member 3 (Abcd3), mRNA.	-1.79	-0.84	0.002930004	19299	NM_008991.2	NM_008991.2
ILMN_2562131	Fmo1	flavin containing monooxygenase 1	-1.78	-0.84	0.02526		AK042457	
ILMN_2954868	Oasl2	2'-5' oligoadenylate synthetase-like 2 (Oasl2), mRNA.	-1.78	-0.84	0.042792667	23962	NM_011854.1	NM_011854.1
ILMN_2756041	Pkm2	pyruvate kinase, muscle (Pkm2), mRNA. XM_979725 XM_979753 XM_979779	-1.78	-0.83	0.028798057	18746	NM_011099.2	NM_011099.2
ILMN_3097950	Asb1	ankyrin repeat and SOCS box-containing 1 (Asb1), transcript variant 2	-1.78	-0.83	0.010963273	65247	NM_023046.4	NM_023046.4
ILMN_1213855	Extl2	exostoses (multiple)-like 2 (Extl2), mRNA.	-1.76	-0.81	0.00648	58193	NM_021388.3	NM_021388.3
ILMN_2958484	Nlrx1	NLR family member X1 (Nlrx1), mRNA.	-1.75	-0.81	0.025932586	270151	NM_178420.2	NM_178420.2
ILMN_3135566	Tsc2	tuberous sclerosis 2 (Tsc2), transcript variant 2, mRNA.	-1.75	-0.81	0.01752	22084	NM_001039363.1	NM_001039363.1
ILMN_1253819	Prkar2b	protein kinase, cAMP dependent regulatory, type II beta (Prkar2b), m	-1.75	-0.81	0.019147642	19088	NM_011158.3	NM_011158.3
ILMN_2992836	Zfp87	zinc finger protein 87 (Zfp87), mRNA.	-1.75	-0.81	0.03880	170763	NM_133228.2	NM_133228.2
ILMN_2781353	Ush2a	Usher syndrome 2A (autosomal recessive, mild) homolog (human) (Ush2a)	-1.75	-0.81	0.03880	22283	NM_021408.2	NM_021408.2
ILMN_1248210	LOC100041290	hypothetical protein LOC100041290 (LOC100041290), mRNA.	-1.74	-0.80	0.021967819	100041290	XM_001476109.1	XM_001476109.1
ILMN_2867158	3110007F17Rik	RIKEN cDNA 3110007F17 gene (3110007F17Rik), mRNA.	-1.74	-0.80	0.03421134	73061	NM_028426.1	NM_028426.1
ILMN_3135409	Elmo2	engulfment and cell motility 2, ced-12 homolog (C. elegans) (Elmo2), transcript variant 1	-1.74	-0.80	0.01417	140579	NM_207705.1	NM_207705.1
ILMN_2787844	Serinc3	serine incorporator 3 (Serinc3), mRNA.	-1.74	-0.80	0.025932586	26943	NM_012032.2	NM_012032.2
ILMN_2501267	Tmc7	transmembrane channel-like gene family 7 (Tmc7), mRNA.	-1.73	-0.79	0.025129603	209760	NM_172476.4	NM_172476.4
ILMN_2912111	D10627	cDNA sequence D10627 (D10627), mRNA.	-1.73	-0.79	0.04193	234358	NM_001013379.2	NM_001013379.2
ILMN_2763772	Rpap3	RNA polymerase II associated protein 3 (Rpap3), mRNA.	-1.73	-0.79	0.01954	71919	NM_028003.2	NM_028003.2
ILMN_1259294	Tmem126b	transmembrane protein 126B (Tmem126b), mRNA.	-1.72	-0.79	0.01798	68472	NM_026734.1	NM_026734.1
ILMN_2851251	Hn1l	hematological and neurological expressed 1-like (Hn1l), mRNA.	-1.72	-0.78	0.03880	52009	NM_198937.2	NM_198937.2
ILMN_2685770	Vangl2	vang-like 2 (van gogh, Drosophila) (Vangl2), mRNA.	-1.72	-0.78	0.02933	93840	NM_033509.3	NM_033509.3
ILMN_3160963	AI316807	expressed sequence AI316807 (AI316807), mRNA.	-1.71	-0.77	0.030787675	102032	NM_001012667.1	NM_001012667.1
ILMN_2589312	Entpd4	ectonucleoside triphosphate diphosphohydrolase 4 (Entpd4), mRNA.	-1.70	-0.76	0.01881207	67464	NM_026174.2	NM_026174.2
ILMN_1256528	Flywch2	FLYWCH family member 2 (Flywch2), mRNA.	-1.68	-0.75	0.01980	76917	NM_029798.2	NM_029798.2
ILMN_1249550	Fkbp9	FK506 binding protein 9 (Fkbp9), mRNA.	-1.68	-0.75	0.02504	27055	NM_012056.1	NM_012056.1
ILMN_2773286	Dorz1	Abhd14a, abhydrolase domain containing 14A	-1.68	-0.75	0.02261		NM_145919	
ILMN_1250182	Cep164	centrosomal protein 164 (Cep164), mRNA.	-1.68	-0.75	0.04113	214552	NM_001081373.1	NM_001081373.1
ILMN_1241211	Gucy1a3	guanylate cyclase 1, soluble, alpha 3 (Gucy1a3), mRNA.	-1.68	-0.74	0.04816268	60596	NM_021896.4	NM_021896.4
ILMN_2600043	Slc35a5	solute carrier family 35, member A5 (Slc35a5), mRNA.	-1.67	-0.74	0.03652	74102	NM_028756.3	NM_028756.3
ILMN_2940642	St6galnac2	ST6 (alpha-N-acetyl-neuraminyl-2,3-beta-galactosyl-1, 3)-N-acetylgalactosaminide alpha-2,6-sialyltransferase 2	-1.66	-0.73	0.04167	20446	NM_009180.3	NM_009180.3
ILMN_1241611	Zfp39	zinc finger protein 39 (Zfp39), mRNA.	-1.64	-0.72	0.00951	22698	NM_011758.2	NM_011758.2
ILMN_2651556	Otof	otoferlin (Otof), transcript variant 2, mRNA.	-1.63	-0.71	0.01825	83762	NM_031875.2	NM_031875.2

Supplementary Table S1. Continued…

ILMN_1241271	Smarcd1	SWI/SNF related, matrix associated, actin dependent regulator of chromatin, subfamily d, member 1	-1.63	-0.70	0.04165		NM_031842	
ILMN_2662990	Xkr8	X Kell blood group precursor related family member 8 homolog (Xkr8)	-1.63	-0.70	0.02363	381560	NM_201368.1	NM_201368.1
ILMN_2645276	Spsb3	splA/ryanodine receptor domain and SOCS box containing 3 (Spsb3)	-1.63	-0.70	0.03058	79043	NM_027141.1	NM_027141.1
ILMN_1256523	Trpc2	transient receptor potential cation channel, subfamily C, member 2 (Trpc2), transcript variant 1	-1.63	-0.70	0.03155	22064	NM_011644.2	NM_011644.2
ILMN_2661082	Lpar2	lysophosphatidic acid receptor 2 (Lpar2), mRNA.	-1.63	-0.70	0.04113	53978	NM_020028.3	NM_020028.3
ILMN_3090545	Dppa5	developmental pluripotency associated 5 (Dppa5), mRNA.	-1.62	-0.70	0.03783255	434423	NM_025274.1	NM_025274.1
ILMN_2944226	Ccdc124	coiled-coil domain containing 124 (Ccdc124), mRNA.	-1.62	-0.70	0.028798057	234388	NM_026964.2	NM_026964.2
ILMN_2678661	Fancc	Fanconi anemia, complementation group C (Fancc), transcript variant 1	-1.58	-0.66	0.01820	14088	NM_007985.2	NM_007985.2
ILMN_2605183	EG232599	predicted gene, EG232599 (EG232599), mRNA.	-1.58	-0.66	0.048673403	232599	NM_177689.3	NM_177689.3
ILMN_2770926	Ap1m1	adaptor-related protein complex AP-1, mu subunit 1 (Ap1m1), mRNA.	-1.56	-0.65	0.026721232	11767	NM_007456.3	NM_007456.3
ILMN_1241225	Dctd	dCMP deaminase (Dctd), mRNA.	-1.56	-0.64	0.01265	320685	NM_178788.3	NM_178788.3
ILMN_2791179	Sult2b1	sulfotransferase family, cytosolic, 2B, member 1 (Sult2b1), mRNA.	-1.55	-0.63	0.02173	54200	NM_017465.1	NM_017465.1
ILMN_2571191	Mrpl3	mitochondrial ribosomal protein L3	-1.55	-0.63	0.043184724		AK054185	
ILMN_2795412	Tmem176a	transmembrane protein 176A (Tmem176a), mRNA.	-1.55	-0.63	0.03155	66058	NM_025326.2	NM_025326.2
ILMN_2737158	Ankle2	ankyrin repeat and LEM domain containing 2 (Ankle2), mRNA.	-1.54	-0.62	0.04639	71782	NM_027922.1	NM_027922.1
ILMN_2702193	Mxra7	matrix-remodelling associated 7 (Mxra7), mRNA.	-1.52	-0.60	0.037781753	67622	NM_026280.2	NM_026280.2
ILMN_1252621	Itfg2	integrin alpha FG-GAP repeat containing 2 (Itfg2), mRNA.	-1.51	-0.60	0.030787675	101142	NM_133927.1	NM_133927.1
ILMN_1251301	Klc4	kinesin light chain 4 (Klc4), mRNA.	-1.51	-0.59	0.015657812	74764	NM_029091.2	NM_029091.2
ILMN_3018711	Dppa5	developmental pluripotency associated 5 (Dppa5), mRNA.	-1.51	-0.59	0.04714064	434423	NM_025274.1	NM_025274.1
ILMN_2973824	Car14	carbonic anhydrase 14 (Car14), mRNA.	-1.51	-0.59	0.038524948	23831	NM_011797.1	NM_011797.1
ILMN_2592810	Map3k3	mitogen-activated protein kinase kinase kinase 3 (Map3k3), mRNA.	1.50	0.59	0.045168124	26406	NM_011947.3	NM_011947.3
ILMN_2672597	Gng10	guanine nucleotide binding protein (G protein), gamma 10 (Gng10), mR	1.51	0.59	0.011701967	14700	NM_025277.3	NM_025277.3
ILMN_1244225	Cdc40	cell division cycle 40 homolog (yeast), transcript variant 3 (Cdc40	1.51	0.60	0.03580	71713	XM_899512.2	XM_899512.2
ILMN_3104118	AW548124	expressed sequence AW548124 (AW548124), mRNA.	1.52	0.60	0.006866085	106522	NM_134117.1	NM_134117.1
ILMN_1222333	Slc2a4	solute carrier family 2 (facilitated glucose transporter), member 4 (Slc2a4)	1.52	0.60	0.03652	20528	NM_009204.2	NM_009204.2
ILMN_2870672	Fbln1	fibulin 1 (Fbln1), mRNA.	1.52	0.60	0.043805897	14114	NM_010180.1	NM_010180.1
ILMN_1217372	Tpmt	thiopurine methyltransferase	1.52	0.60	0.029008582		AK002335	
ILMN_2675090	Dusp15	dual specificity phosphatase-like 15 (Dusp15), mRNA.	1.52	0.60	0.010963273	252864	NM_145744.2	NM_145744.2
ILMN_2775402	Shisa4	shisa homolog 4 (Xenopus laevis) (Shisa4), mRNA.	1.52	0.61	0.02072	77552	NM_175259.4	NM_175259.4
ILMN_1260566	4930429B21Rik	RIKEN cDNA 4930429B21 gene (4930429B21Rik), misc RNA.	1.52	0.61	0.024856472	67576	XR_035269.1	XR_035269.1
ILMN_2872419	Zbtb8os	zinc finger and BTB domain containing 8 opposite strand (Zbtb8os), m	1.52	0.61	0.02691	67106	NM_025970.1	NM_025970.1
ILMN_2777650	Catna2	Ctnna2, catenin (cadherin associated protein), alpha 2	1.54	0.62	0.04746		NM_145732	
ILMN_2585676	Slc6a4	solute carrier family 6 (neurotransmitter transporter, serotonin), member 4	1.55	0.63	0.01851		AK078634	
ILMN_1253189	2610014I16Rik	RIKEN cDNA 2610014I16 gene (2610014I16Rik), mRNA.	1.55	0.63	0.02933	66300	XM_001480001.1	XM_001480001.1
ILMN_2719973	Gpc3	glypican 3 (Gpc3), mRNA.	1.57	0.65	0.02898	14734	NM_016697.2	NM_016697.2
ILMN_2757283	Clcnkb	chloride channel Kb (Clcnkb), mRNA.	1.57	0.65	0.041398313	56365	NM_019701.1	NM_019701.1
ILMN_2497410	Pcdhb6	protocadherin beta 6	1.58	0.66	0.03925		NM_053131	
ILMN_2993720	Commd6	COMM domain containing 6 (Commd6), mRNA. XM_919080	1.59	0.67	0.03974	66200	NM_001033132.1	NM_001033132.1
ILMN_1242946	LOC629364	similar to actin related protein 2/3 complex, subunit 5 (LOC629364)	1.59	0.67	0.04067	629364	XM_001479142.1	XM_001479142.1
ILMN_2628339	Bin1	bridging integrator 1 (Bin1), transcript variant 2, mRNA.	1.59	0.67	0.01825	30948	NM_001083334.1	NM_001083334.1
ILMN_2486863	Rai16	retinoic acid induced 16 (Rai16), mRNA.	1.59	0.67	0.01236	239170	XM_001480783.1	XM_001480783.1
ILMN_1249743	LOC100048638	similar to lens fiber cell beaded-filament structure protein (LOC100048638)	1.59	0.67	0.04735	100048638	XR_034832.1	XR_034832.1
ILMN_3143088	AI449175	expressed sequence AI449175 (AI449175), transcript variant 1, mRNA.	1.59	0.67	0.045955587	234362	NM_172754.2	NM_172754.2
ILMN_1235657	Rnase4	ribonuclease, RNase A family 4 (Rnase4), transcript variant 1, mRNA.	1.60	0.67	0.03421134	58809	NM_021472.3	NM_021472.3
ILMN_2416488	Usp37	ubiquitin specific peptidase 37 (Usp37), mRNA.	1.60	0.68	0.02595	319651	NM_176972.3	NM_176972.3
ILMN_1256633	LOC100045567	similar to purine nucleoside phosphorylase (LOC100045567), mRNA.	1.60	0.68	0.01978	100045567	XM_001474536.1	XM_001474536.1
ILMN_1219169	Gfer	growth factor, erv1 (S. cerevisiae)-like (augmenter of liver regeneration) (Gfer)	1.60	0.68	0.03693	11692	NM_023040.3	NM_023040.3
ILMN_1218920	D130043K22Rik	RIKEN cDNA D130043K22 gene (D130043K22Rik), mRNA.	1.61	0.69	0.034129914	210108	NM_001081051.1	NM_001081051.1
ILMN_1217021	Sfxn3	sideroflexin 3 (Sfxn3), mRNA.	1.62	0.69	0.030083355	94280	NM_053197.2	NM_053197.2
ILMN_2726905	Gnb4	guanine nucleotide binding protein (G protein), beta 4 (Gnb4), mRNA.	1.62	0.70	0.03648	14696	NM_013531.3	NM_013531.3
ILMN_2672380	Arhgap22	Rho GTPase activating protein 22 (Arhgap22), mRNA.	1.62	0.70	0.04237	239027	NM_153800.3	NM_153800.3
ILMN_2430220	Tmem2	transmembrane protein 2 (Tmem2), transcript variant 2, mRNA.	1.62	0.70	0.03971729	83921	NM_001033759.1	NM_001033759.1
ILMN_2663576	Mic2l1	Cd99l2, CD99 antigen-like 2	1.62	0.70	0.03289		NM_138309.1	NM_138309.1
ILMN_2820369	Gstcd	glutathione S-transferase, C-terminal domain containing (Gstcd), mRN	1.64	0.72	0.036654163	67553	NM_026231.2	NM_026231.2
ILMN_3040069	Hist2h3c1	histone cluster 2, H3c1 (Hist2h3c1), transcript variant 2, mRNA.	1.65	0.72	0.027386699	15077	NM_178216.1	NM_178216.1
ILMN_1245717	Epb4.9	erythrocyte protein band 4.9 (Epb4.9), mRNA.	1.65	0.72	0.02355	13829	NM_013514.3	NM_013514.3
ILMN_2737129	Klf12	Kruppel-like factor 12	1.65	0.72	0.04442		NM_010636	
ILMN_1256137	Hecw1	HECT, C2 and WW domain containing E3 ubiquitin protein ligase 1 (Hecw1)	1.65	0.72	0.03580043	94253	NM_001081348.2	NM_001081348.2
ILMN_1234387	Acp1	acid phosphatase 1, soluble	1.65	0.73	0.01685		AK019186	
ILMN_1260389	Lin7b	lin-7 homolog B (C. elegans) (Lin7b), mRNA.	1.66	0.73	0.04771	22342	NM_011698.1	NM_011698.1
ILMN_2896768	Cbr3	carbonyl reductase 3 (Cbr3), mRNA.	1.68	0.75	0.01229	109857	NM_173047.2	NM_173047.2
ILMN_2562107	Usp14	ubiquitin specific peptidase 14	1.69	0.76	0.00912		AK042356	
ILMN_1223215	LOC636687	similar to 6820431F20Rik protein (LOC636687), mRNA.	1.70	0.76	0.02816	636687	XM_912173.3	XM_912173.3
ILMN_1231621	Unc5h3	Unc5c, unc-5 netrin receptor C	1.70	0.76	0.015066082		AK034558	
ILMN_2535238	Xkr4	X Kell blood group precursor related family member 4 (Xkr4), mRNA.	1.72	0.79	0.00912	497097	NM_001011874.1	NM_001011874.1
ILMN_2511806	Clec16a	C-type lectin domain family 16, member A (Clec16a), mRNA.	1.73	0.79	0.02859	74374	NM_177562.4	NM_177562.4
ILMN_2643057	Trappc6a	trafficking protein particle complex 6A (Trappc6a), mRNA.	1.73	0.79	0.01881	67091	NM_025960.3	NM_025960.3
ILMN_1251874	Pscd2	pleckstrin homology, Sec7 and coiled-coil domains 2 (Pscd2), mRNA.	1.73	0.79	0.04771	19158	NM_011181.2	NM_011181.2
ILMN_1214738	Snapc3	small nuclear RNA activating complex, polypeptide 3 (Snapc3), mRNA.	1.73	0.79	0.02486	77634	NM_029949.3	NM_029949.3
ILMN_1223261	Mkrn1	makorin, ring finger protein, 1	1.74	0.80	0.00711		AK005137	
ILMN_1240445	Stard4	StAR-related lipid transfer (START) domain containing 4 (Stard4), mR	1.77	0.82	0.027312322	170459	NM_133774.4	NM_133774.4

Supplementary Table S1. Continued…

ILMN_2687403	Fcgr3	Fc receptor, IgG, low affinity III (Fcgr3), mRNA.	1.77	0.83	0.00688	14131	NM_010188.4	NM_010188.4
ILMN_2616772	Slc39a9	solute carrier family 39 (zinc transporter), member 9, transcript variant 2 (Slc39a9)	1.78	0.84	0.02486	328133	XM_891220.2	XM_891220.2
ILMN_2579266	Neo1	neogenin	1.79	0.84	0.02661		AK084609	
ILMN_2864844	Vps33b	vacuolar protein sorting 33B (yeast) (Vps33b), mRNA.	1.79	0.84	0.04219	233405	NM_178070.2	NM_178070.2
ILMN_1237595	Fv1	Friend virus susceptibility 1 (Fv1), mRNA.	1.79	0.84	0.044227295	14349	NM_010244.3	NM_010244.3
ILMN_3060788	9030025P20Rik	RIKEN cDNA 9030025P20 gene (9030025P20Rik), transcript variant 2, mR	1.80	0.85	0.027452955	381062	NM_001034891.2	NM_001034891.2
ILMN_1241482	LOC100039607	hypothetical protein LOC100039607 (LOC100039607), mRNA.	1.80	0.85	0.02486	100039607	XM_001473184.1	XM_001473184.1
ILMN_2610148	LOC100040897	similar to Predicted gene, EG545013, transcript variant 1 (LOC100040897)	1.81	0.86	0.03329	100040897	XM_001475780.1	XM_001475780.1
ILMN_2732642	Kptn	kaptin (Kptn), mRNA.	1.82	0.86	0.019147642	70394	NM_133727.1	NM_133727.1
ILMN_1226311	Chd9	chromodomain helicase DNA binding protein 9	1.84	0.88	0.04370		XM_284439	
ILMN_2566538	Fbn2	fibrillin 2	1.84	0.88	0.01096		AK053721	
ILMN_2529089	Csmd3	CUB and Sushi multiple domains 3, transcript variant 2 (Csmd3), mRN	1.84	0.88	0.009510696	239420	XM_484467.4	XM_484467.4
ILMN_2655015	Alad	aminolevulinate, delta-, dehydratase (Alad), mRNA.	1.86	0.89	0.02997	17025	NM_008525.3	NM_008525.3
ILMN_3070389	ENSMUSG00000068790	predicted gene, ENSMUSG00000068790 (ENSMUSG00000068790), mRNA.	1.87	0.90	0.01138	545007	NM_001029930.1	NM_001029930.1
ILMN_2958159	Eno1	enolase 1, alpha non-neuron (Eno1), mRNA.	1.87	0.91	0.00699	13806	NM_023119.1	NM_023119.1
ILMN_2543433	5330426P16Rik	RIKEN cDNA 5330426P16 gene (5330426P16Rik), mRNA.	1.89	0.92	0.02577	68190	XM_916412.3	XM_916412.3
ILMN_3136656	Nrp2	neuropilin 2 (Nrp2), transcript variant 5, mRNA.	1.90	0.93	0.04140	18187	NM_001077406.1	NM_001077406.1
ILMN_2831656	Epha3	Eph receptor A3 (Epha3), mRNA.	1.90	0.93	0.02997	13837	NM_010140.1	NM_010140.1
ILMN_2546510	EG434402	predicted gene, EG434402 (EG434402), mRNA.	1.94	0.95	0.03783	434402	XM_001479948.1	XM_001479948.1
ILMN_2667352	Glo1	glyoxalase 1 (Glo1), mRNA.	1.96	0.97	0.00699	109801	NM_025374.2	NM_025374.2
ILMN_3067404	Zfp386	zinc finger protein 386 (Kruppel-like) (Zfp386), transcript variant 1	1.96	0.97	0.04838	56220	NM_001004066.2	NM_001004066.2
ILMN_1236500	Glce	glucuronyl C5-epimerase	1.97	0.98	0.01833		NM_033320	
ILMN_2591345	Csnk1g1	casein kinase 1, gamma 1 (Csnk1g1), mRNA.	1.98	0.99	0.04718	214897	NM_173185.2	NM_173185.2
ILMN_1244801	2410066E13Rik	RIKEN cDNA 2410066E13 gene (2410066E13Rik), mRNA.	1.99	1.00	0.04165	68235	NM_026629.3	NM_026629.3
ILMN_1230916	LOC433955	similar to HSPC008 (LOC433955), misc RNA.	2.00	1.00	0.03648413	433955	XR_033167.1	XR_033167.1
ILMN_1215984	Clcn4-2	Clcn4, chloride channel, voltage-sensitive 4	2.00	1.00	0.04094		AK088317	
ILMN_3001341	Cnnm1	cyclin M1 (Cnnm1), mRNA.	2.00	1.00	0.01851	83674	NM_031396.1	NM_031396.1
ILMN_1248763	Thex1	three prime histone mRNA exonuclease 1 (Thex1), mRNA.	2.02	1.01	0.03783	67276	NM_026067.2	NM_026067.2
ILMN_2600659	Epb4.1l1	erythrocyte protein band 4.1-like 1 (Epb4.1l1), transcript variant 1	2.03	1.02	0.02106	13821	NM_013510.3	NM_013510.3
ILMN_1227722	Tgfb1i1	transforming growth factor beta 1 induced transcript 1 (Tgfb1i1)	2.03	1.02	0.02799	21804	NM_009365.2	NM_009365.2
ILMN_2955694	Spag1	sperm associated antigen 1 (Spag1), mRNA.	2.04	1.03	0.02905	26942	NM_012031.1	NM_012031.1
ILMN_3099758	Pi4k2b	phosphatidylinositol 4-kinase type 2 beta (Pi4k2b), transcript variant 2	2.05	1.03	0.02177	67073	NM_028744.1	NM_028744.1
ILMN_2841280	Habp4	hyaluronic acid binding protein 4 (Habp4), mRNA.	2.05	1.03	0.03550	56541	NM_019986.1	NM_019986.1
ILMN_2607926	Kdelr2	KDEL (Lys-Asp-Glu-Leu) endoplasmic reticulum protein retention receptor 2 (Kdelr2)	2.05	1.04	0.04997	66913	NM_025841.3	NM_025841.3
ILMN_1226295	FRMD3	FERM domain containing 3	2.05	1.04	0.034161568		NM_172869	
ILMN_2531100	Gm949	gene model 949, (NCBI) (Gm949), mRNA.	2.06	1.04	0.02557	381142	NM_001033446.2	NM_001033446.2
ILMN_1220594	Fv1	Friend virus susceptibility 1 (Fv1), mRNA.	2.07	1.05	0.04228096	14349	NM_010244.3	NM_010244.3
ILMN_1247028	Lrrc27	leucine rich repeat containing 27 (Lrrc27), mRNA.	2.07	1.05	0.022351176	76612	NM_027164.1	NM_027164.1
ILMN_1239134	Lypd1	Ly6/Plaur domain containing 1 (Lypd1), mRNA.	2.08	1.05	0.03681	72585	NM_145100.3	NM_145100.3
ILMN_1243548	AMPH	amphiphysin	2.08	1.06	0.03924928		AK047144	
ILMN_2846297	3110035E14Rik	RIKEN cDNA 3110035E14 gene (3110035E14Rik), mRNA.	2.09	1.06	0.007164895	76982	NM_178399.2	NM_178399.2
ILMN_1236474	SLC6A1	solute carrier family 6 (neurotransmitter transporter, GABA), member 1	2.10	1.07	0.03783255		AK053831	
ILMN_2733852	Gnb1	guanine nucleotide binding protein (G protein), beta 1 (Gnb1), mRNA.	2.15	1.10	0.01825	14688	NM_008142.3	NM_008142.3
ILMN_2718406	Rab26	RAB26, member RAS oncogene family, transcript variant 1 (Rab26)	2.15	1.11	0.00688	328778	XM_283428.6	XM_283428.6
ILMN_2566477	THRAP3	thyroid hormone receptor associated protein 3	2.16	1.11	0.02619		AK050208	
ILMN_1251909	Vars2	valyl-tRNA synthetase 2, mitochondrial (putative) (Vars2), mRNA.	2.17	1.12	0.03254	68915	NM_175137.3	NM_175137.3
ILMN_2925570	Nubp2	nucleotide binding protein 2 (Nubp2), mRNA.	2.17	1.12	0.00818	26426	NM_011956.2	NM_011956.2
ILMN_2909238	Spnb1	spectrin beta 1 (Spnb1), mRNA.	2.18	1.13	0.02486	20741	NM_013675.3	NM_013675.3
ILMN_2935012	Anxa4	annexin A4 (Anxa4), mRNA.	2.18	1.13	0.037967987	11746	NM_013471.1	NM_013471.1
ILMN_2697790	Fgfr1op	Fgfr1 oncogene partner (Fgfr1op), mRNA.	2.19	1.13	0.00716	75296	NM_201230.4	NM_201230.4
ILMN_1213858	Itgb3bp	integrin beta 3 binding protein (beta3-endonexin) (Itgb3bp), mRNA.	2.19	1.13	0.012745504	67733	NM_026348.3	NM_026348.3
ILMN_1244545	OCIAD1	OCIA domain containing 1	2.22	1.15	0.015777528		AK078102	
ILMN_2984110	Plvap	plasmalemma vesicle associated protein (Plvap), mRNA.	2.22	1.15	0.02066799	84094	NM_032398.1	NM_032398.1
ILMN_2672772	Abhd1	abhydrolase domain containing 1 (Abhd1), transcribed RNA.	2.24	1.16	0.01798	57742	NR_003522.1	NR_003522.1
ILMN_2642985	Hsd17b11	hydroxysteroid (17-beta) dehydrogenase 11 (Hsd17b11), mRNA.	2.26	1.18	0.04997015	114664	NM_053262.3	NM_053262.3
ILMN_2815889	Grwd1	glutamate-rich WD repeat containing 1 (Grwd1), mRNA.	2.28	1.19	0.03421	101612	NM_153419.1	NM_153419.1
ILMN_1240677	Gadd45gip1	growth arrest and DNA-damage-inducible, gamma interacting protein 1, (Gadd45gip1)	2.29	1.19	0.02331	102060	NM_183358.3	NM_183358.3
ILMN_1229634	Usp29	ubiquitin specific peptidase 29 (Usp29), mRNA.	2.30	1.20	0.018513808	57775	NM_021323.2	NM_021323.2
ILMN_2519313	Tmod4	tropomodulin 4 (Tmod4), mRNA.	2.30	1.20	0.01175	50874	NM_016712.2	NM_016712.2
ILMN_2419998	Soat1	sterol O-acyltransferase 1 (Soat1), mRNA.	2.31	1.21	0.04455	20652	NM_009230.3	NM_009230.3
ILMN_2459388	TTLL12	tubulin tyrosine ligase-like family, member 12	2.31	1.21	0.00912		NM_183017	
ILMN_1216690	Samd11	sterile alpha motif domain containing 11 (Samd11), mRNA.	2.33	1.22	0.00340	231004	NM_173736.2	NM_173736.2
ILMN_2430229	Lats1	large tumor suppressor	2.35	1.23	0.029901467			
ILMN_1229544	LOC100041569	hypothetical protein LOC100041569 (LOC100041569), mRNA.	2.36	1.24	0.03329	100041569	XM_001476596.1	XM_001476596.1
ILMN_2752545	Lama3	laminin, alpha 3	2.37	1.24	0.02524		XM_128926.3	XM_128926.3
ILMN_1255527	Fmr1	fragile X mental retardation syndrome 1	2.37	1.25	0.03353		AK080898	
ILMN_2482662	A730036E13Rik	RIKEN cDNA A730036E13 gene (A730036E13Rik), mRNA.	2.41	1.27	0.03880	320291	XM_989657.2	XM_989657.2
ILMN_2694782	2410076I21Rik	RIKEN cDNA 2410076I21 gene, transcript variant 1 (2410076I21Rik)	2.41	1.27	0.02427	73673	XM_134948.5	XM_134948.5
ILMN_2901363	2310002B06Rik	RIKEN cDNA 2310002B06 gene (2310002B06Rik), mRNA.	2.42	1.27	0.03449	53951	NM_181649.4	NM_181649.4
ILMN_2625114	Cdkal1	CDK5 regulatory subunit associated protein 1-like 1	2.43	1.28	0.00243		NM_144536.1	NM_144536.1
ILMN_2699307	Hebp1	heme binding protein 1 (Hebp1), mRNA.	2.43	1.28	0.04431	15199	NM_013546.2	NM_013546.2

Supplementary Table S1. Continued…

ILMN_2904703	Ctsl	cathepsin L (Ctsl), mRNA.	2.51	1.33	0.022304757	13039	NM_009984.2	NM_009984.2
ILMN_1242672	EG434168	predicted gene, EG434168 (EG434168), mRNA.	2.53	1.34	0.009116249	434168	XM_973360.2	XM_973360.2
ILMN_1243314	LOC622994	hypothetical LOC622994 (LOC622994), misc RNA.	2.54	1.34	0.01013	622994	XR_031862.1	XR_031862.1
ILMN_1227376	BC042720	cDNA sequence BC042720 (BC042720), mRNA.	2.54	1.34	0.01626	329178	XM_990462.1	XM_990462.1
ILMN_2658227	Pparbp	med1, mediator complex subunit 1	2.55	1.35	0.042792667		NM_013634.1	NM_013634.1
ILMN_2669793	Ccnd1	cyclin D1 (Ccnd1), mRNA.	2.55	1.35	0.03580043	12443	NM_007631.2	NM_007631.2
ILMN_2575087	PKN1	protein kinase N1	2.57	1.36	0.01170		AK050643	
ILMN_2570478	SNX10	sorting nexin 10	2.66	1.41	0.03008		AK042505	
ILMN_2729289	Matn4	matrilin 4 (Matn4), mRNA.	2.69	1.43	0.043498833	17183	NM_013592.2	NM_013592.2
ILMN_2705578	Snx30	sorting nexin family member 30 (Snx30), mRNA.	2.74	1.45	0.007164895	209131	NM_172468.2	NM_172468.2
ILMN_1228540	6430590A10Rik	RIKEN cDNA 6430590A10 gene (6430590A10Rik), misc RNA.	2.74	1.46	0.01685	319850	XR_035434.1	XR_035434.1
ILMN_2421925	WDFY1	WD repeat and FYVE domain containing 1	2.92	1.54	0.02799		NM_027057	
ILMN_1236799	Ttll2	tubulin tyrosine ligase-like family, member 2 (Ttll2), mRNA.	2.95	1.56	0.01666	625850	NM_001098267.1	NM_001098267.1
ILMN_1215660	DMTF1	cyclin D binding myb-like transcription factor 1	2.99	1.58	0.04202		AK030508	
ILMN_2664909	Mesdc1	mesoderm development candidate 1 (Mesdc1), mRNA.	3.06	1.61	0.01886	80889	NM_030705.4	NM_030705.4
ILMN_2724055	1700001E04Rik	RIKEN cDNA 1700001E04 gene (1700001E04Rik), mRNA.	3.07	1.62	0.012357241	75438	NM_029288.2	NM_029288.2
ILMN_2624176	Nsun2	NOL1/NOP2/Sun domain family member 2 (Nsun2), mRNA.	3.11	1.63	0.02022	28114	NM_145354.3	NM_145354.3
ILMN_1260199	Casc4	cancer susceptibility candidate 4 (Casc4), transcript variant 1, mRN	3.12	1.64	0.02177	319996	NM_177054.3	NM_177054.3
ILMN_1237719	Grasp	GRP1 (general receptor for phosphoinositides 1)-associated scaffold protein	3.14	1.65	0.021725355		AK051199	
ILMN_2881296	Tmem66	transmembrane protein 66 (Tmem66), mRNA.	3.18	1.67	0.00492	67887	NM_026432.2	NM_026432.2
ILMN_2481836	LOC668047	similar to ring finger protein 170 (LOC668047), mRNA.	3.21	1.68	0.010963273	668047	XM_001001100.1	XM_001001100.1
ILMN_1249578	Sorl1	sortilin-related receptor, LDLR class A repeats-containing (Sorl1),	3.34	1.74	0.01800	20660	NM_011436.3	NM_011436.3
ILMN_1224719	LOC665032	similar to ribosomal protein (LOC665032), mRNA.	3.40	1.76	0.01046	665032	XM_991641.1	XM_991641.1
ILMN_2761128	Clasp2	CLIP associating protein 2 (Clasp2), transcript variant 2, mRNA.	3.43	1.78	0.009116249	76499	NM_001081960.1	NM_001081960.1
ILMN_1222253	Neto2	neuropilin (NRP) and tolloid (TLL)-like 2, transcript variant 8 (Neto2)	3.49	1.80	0.015777528	74513	XM_922064.2	XM_922064.2
ILMN_2968123	Slc7a14	solute carrier family 7 (cationic amino acid transporter, y+ system)	3.65	1.87	0.04303	241919	NM_172861.2	NM_172861.2
ILMN_1225187	LOC675899	similar to H2A histone family, member Z (LOC675899), mRNA.	3.75	1.91	0.03421	675899	XM_985882.1	XM_985882.1
ILMN_1223496	LOC100046025	similar to mKIAA1230 protein, transcript variant 1 (LOC100046025),	3.86	1.95	0.006992973	100046025	XM_001475823.1	XM_001475823.1
ILMN_2644008	Chl1	cell adhesion molecule L1-like	4.17	2.06	0.01170		NM_007697.1	NM_007697.1
ILMN_2477213	LOC100045240	hypothetical protein LOC100045240 (LOC100045240), mRNA.	4.24	2.09	0.006876021	100045240	XM_001473915.1	XM_001473915.1
ILMN_1246800	Serpina3n	serine (or cysteine) peptidase inhibitor, clade A, member 3N (Serpina3n)	4.30	2.10	0.02666	20716	NM_009252.2	NM_009252.2
ILMN_2705097	Deadc1	deaminase domain containing 1 (Deadc1), mRNA.	4.45	2.15	0.010145757	66757	NM_025748.3	NM_025748.3
ILMN_1214706	SASH1	SAM and SH3 domain containing 1	4.52	2.18	0.016258812		AK049453	
ILMN_2800466	Tpmt	thiopurine methyltransferase (Tpmt), mRNA.	4.62	2.21	0.00823	22017	NM_016785.1	NM_016785.1
ILMN_2502614	Ttc15	tetratricopeptide repeat domain 15 (Ttc15), mRNA.	4.97	2.31	0.04193	217449	NM_178811.3	NM_178811.3
ILMN_2590520	H2-Bl	histocompatibility 2, blastocyst (H2-Bl), mRNA.	5.00	2.32	0.00912	14963	NM_008199.1	NM_008199.1
ILMN_3022092	LOC544988	hypothetical protein LOC544988 (LOC544988), mRNA.	5.06	2.34	0.01851	544988	NM_001024712.1	NM_001024712.1
ILMN_1254148	GOLGA7	golgi autoantigen, golgin subfamily a, 7	5.20	2.38	0.01046493		AK089856	
ILMN_2752569	Paip1	polyadenylate binding protein-interacting protein 1 (Paip1), transcript variant 1	5.28	2.40	0.027312322	218693	NM_145457.3	NM_145457.3
ILMN_1240828	LOC100040657	similar to 1700001E04Rik protein (LOC100040657), mRNA.	5.30	2.41	0.005568372	100040657	XM_001475283.1	XM_001475283.1
ILMN_1229454	Cuedc1	CUE domain containing 1 (Cuedc1), mRNA.	5.32	2.41	0.018513808	103841	NM_198013.1	NM_198013.1
ILMN_2667463	Ocel1	occludin/ELL domain containing 1 (Ocel1), mRNA.	5.72	2.52	0.002151931	77090	NM_029865.1	NM_029865.1
ILMN_1216142	Masp2	mannan-binding lectin serine peptidase 2 (Masp2), transcript variant	5.98	2.58	0.00648	17175	NM_010767.3	NM_010767.3
ILMN_2604282	Sfrp1	secreted frizzled-related protein 1	6.50	2.70	0.01977		NM_013834.1	NM_013834.1
ILMN_2838965	4930455C21Rik	RIKEN cDNA 4930455C21 gene (4930455C21Rik), mRNA.	7.11	2.83	0.034819607	76916	NM_024273.1	NM_024273.1
ILMN_2969172	Tmem87a	transmembrane protein 87A (Tmem87a), mRNA.	7.43	2.89	0.02727	211499	NM_173734.2	NM_173734.2
ILMN_2559669	PBX1	pre B cell leukemia homeobox 1	9.33	3.22	0.007114192		AK037006	
ILMN_2729513	Hbb-b2	hemoglobin, beta adult minor chain (Hbb-b2), mRNA.	9.56	3.26	0.01861	15130	NM_016956.2	NM_016956.2
ILMN_2728465	Sstr1	somatostatin receptor 1 (Sstr1), mRNA.	11.07	3.47	0.00333	20605	NM_009216.3	NM_009216.3
ILMN_1214408	LOC666403	similar to ribosomal protein S2 (LOC666403), misc RNA.	36.82	5.20	0.00383	666403	XR_034389.1	XR_034389.1

[*] Fold change in 129 relative to B6

Supplementary Table S2. Differentially expressed genes between nm1054 129 and nm1054 B6.

Probe ID	Gene Name	Gene Description	Fold Change*	Log FC	p (Corr)	Entrez Gene ID	Accession Number	RefSeq ID
ILMN_2825109	Zfp330	zinc finger protein 330 (Zfp330), mRNA.	-44.02	-5.46	0.000813	30932	NM_145600.1	NM_145600.1
ILMN_2895177	Epm2aip1	EPM2A (laforin) interacting protein 1 (Epm2aip1), mRNA.	-31.70	-4.99	0.001554	77781	NM_175266.2	NM_175266.2
ILMN_1242107	Cox7a2l	cytochrome c oxidase subunit VIIa polypeptide 2-like	-29.01	-4.86	0.001565787		XM_123188.1	XM_123188.1
ILMN_1218868	Cops8	COP9 (constitutive photomorphogenic) homolog, subunit 8 (Arabidopsis thaliana) (Cops8)	-20.81	-4.38	0.001364	108679	NM_133805.3	NM_133805.3
ILMN_2768053	Supt16h	suppressor of Ty 16 homolog (S. cerevisiae) (Supt16h), mRNA.	-20.56	-4.36	0.003881304	114741	NM_033618.3	NM_033618.3
ILMN_3009910	Rbm13	RNA binding motif protein 13 (Rbm13), mRNA.	-16.12	-4.01	0.001565787	67920	NM_026453.1	NM_026453.1
ILMN_1256161	Myt1l	myelin transcription factor 1-like	-15.43	-3.95	0.001566		AK012660	
ILMN_1242829	Prdx2	peroxiredoxin 2 (Prdx2), mRNA.	-14.78	-3.89	0.002181379	21672	NM_011563.2	NM_011563.2
ILMN_3161626	Prkag2	protein kinase, AMP-activated, gamma 2 non-catalytic subunit (Prkag2	-14.38	-3.85	0.001873	108099	NM_145401.1	NM_145401.1
ILMN_2514377	Cxadr	us and adenovirus receptor(Cxadr)	-14.14	-3.82	0.001368498		NM_009988	
ILMN_2730005	Rpl29	ribosomal protein L29 (Rpl29), mRNA.	-13.90	-3.80	0.001554	19944	NM_009082.2	NM_009082.2
ILMN_2746483	Wdr82	WD repeat domain containing 82 (Wdr82), mRNA.	-13.20	-3.72	0.001566	77305	NM_029896.1	NM_029896.1
ILMN_2776922	Glrx1	glutaredoxin	-12.45	-3.64	0.002545065		NM_053108	
ILMN_2507182	Tomm22	translocase of outer mitochondrial membrane 22 homolog (Tomm22), nuclear gene encoding mitochondrial protein	-12.04	-3.59	0.004066	223696	NM_172609.3	NM_172609.3
ILMN_1221102	Arl5a	ADP-ribosylation factor-like 5A (Arl5a), mRNA.	-11.08	-3.47	0.00301546	75423	NM_182994.2	NM_182994.2
ILMN_2678019	Psmd8	proteasome (prosome, macropain) 26S subunit, non-ATPase, 8 (Psmd8),	-10.90	-3.45	0.011974	57296	NM_026545.2	NM_026545.2
ILMN_2691261	Ndufb10	NADH dehydrogenase (ubiquinone) 1 beta subcomplex, 10	-10.55	-3.40	0.004258		XM_128594.4	XM_128594.4
ILMN_1219583	Capn2	calpain 2 (Capn2), mRNA.	-10.48	-3.39	0.004589092	12334	NM_009794.1	NM_009794.1
ILMN_2455850	Gpr137b-ps	G protein-coupled receptor 137B, pseudogene (Gpr137b-ps), non-coding RNA.	-10.46	-3.39	0.001873	664862	NR_003568.1	NR_003568.1
ILMN_2425376	Cttnbp2	cortactin binding protein 2, transcript variant 5 (Cttnbp2), mRNA.	-10.17	-3.35	0.001873	30785	XM_987526.1	XM_987526.1
ILMN_2615468	Fgfr1op2	FGFR1 oncogene partner 2 (Fgfr1op2), mRNA.	-9.94	-3.31	0.005133764	67529	NM_026218.2	NM_026218.2
ILMN_2769656	Picalm	phosphatidylinositol binding clathrin assembly protein	-9.38	-3.23	0.002860728		NM_146194	
ILMN_2484987	Man2b1	mannosidase 2, alpha B1 (Man2b1), mRNA.	-9.17	-3.20	0.005770014	17159	NM_010764.2	NM_010764.2
ILMN_2733542	LOC100044862	similar to Fbxl3 protein (LOC100044862), mRNA.	-8.91	-3.16	0.001554	100044862	XM_001473206.1	XM_001473206.1
ILMN_1257724	Adamts9	a disintegrin-like and metallopeptidase (reprolysin type) with thrombospondin type 1 motif, 9	-8.69	-3.12	0.002117617			
ILMN_2966034	Zfp365	zinc finger protein 365 (Zfp365), mRNA.	-8.50	-3.09	0.003881	216049	NM_178679.2	NM_178679.2
ILMN_1238801	Arl3	ADP-ribosylation factor-like 3 (Arl3), mRNA.	-8.22	-3.04	0.009200	56350	NM_019718.2	NM_019718.2
ILMN_2658392	Atp2c1	ATPase, Ca++-sequestering (Atp2c1), mRNA.	-8.17	-3.03	0.002807294	235574	NM_175025.2	NM_175025.2
ILMN_3160292	Akr1c19	aldo-keto reductase family 1, member C19 (Akr1c19), mRNA.	-7.96	-2.99	0.00875329	432720	NM_001013785.2	NM_001013785.2
ILMN_1245850	4933427D14Rik	RIKEN cDNA 4933427D14 gene (4933427D14Rik), mRNA.	-7.83	-2.97	0.013060313	74477	NM_028963.2	NM_028963.2
ILMN_2700233	Ccng2	cyclin G2 (Ccng2), mRNA.	-7.79	-2.96	0.004346	12452	NM_007635.3	NM_007635.3
ILMN_1235372	Hbb-b1	hemoglobin, beta adult major chain (Hbb-b1), mRNA.	-7.55	-2.92	0.009647857	15129	NM_008220.3	NM_008220.3
ILMN_1220270	Mef2c	myocyte enhancer factor 2C	-7.41	-2.89	0.019092		AK047994	
ILMN_2737296	Lars2	leucyl-tRNA synthetase, mitochondrial (Lars2), nuclear gene encoding mitochondrial protein	-7.32	-2.87	0.002860728	102436	NM_153168.2	NM_153168.2
ILMN_1218118	Tia1	cytotoxic granule-associated RNA binding protein 1	-7.15	-2.84	0.007005		AK009502	
ILMN_1250335	Fin15	Gsdme, gasdermin E	-7.13	-2.83	0.001565787		NM_008016.1	NM_008016.1
ILMN_2704027	Slc25a18	solute carrier family 25 (mitochondrial carrier), member 18 (Slc25a18)	-7.05	-2.82	0.021365	71803	NM_001081048.1	NM_001081048.1
ILMN_1249654	Sfrs16	Clasrp, CLK4-associating serine/arginine rich protein	-7.00	-2.81	0.001566		NM_016680	
ILMN_1253773	Nudt6	nudix (nucleoside diphosphate linked moiety X)-type motif 6 (Nudt6),	-6.92	-2.79	0.003487	229228	NM_153561.2	NM_153561.2
ILMN_2690232	BC068157	cDNA sequence BC068157 (BC068157), mRNA.	-6.87	-2.78	0.004756	73072	NM_207203.1	NM_207203.1
ILMN_1255287	Mela	melanoma antigen	-6.76	-2.76	0.009648		NM_008581	
ILMN_2696182	Actl6b	actin-like 6B (Actl6b), mRNA.	-6.38	-2.67	0.010241	83766	NM_031404.4	NM_031404.4
ILMN_1223734	Atf4	activating transcription factor 4 (Atf4), mRNA.	-6.33	-2.66	0.004784712	11911	NM_009716.2	NM_009716.2
ILMN_2449449	Zfp68	zinc finger protein 68 (Zfp68), transcript variant 1, mRNA.	-6.32	-2.66	0.003444308	24135	NM_013844.2	NM_013844.2
ILMN_2893063	Rnf219	ring finger protein 219 (Rnf219), mRNA.	-6.25	-2.64	0.000813	72486	NM_026047.4	NM_026047.4
ILMN_1249638	Rbbp4	retinoblastoma binding protein 4 (Rbbp4), mRNA.	-6.06	-2.60	0.004755816	19646	NM_009030.3	NM_009030.3
ILMN_1243212	Sparc	secreted acidic cysteine rich glycoprotein (Sparc), mRNA.	-5.97	-2.58	0.005844	20692	NM_009242.3	NM_009242.3
ILMN_1251839	Myo7a	myosin VIIa (Myo7a), mRNA.	-5.85	-2.55	0.006630	17921	NM_008663.2	NM_008663.2
ILMN_2775050	Arv1	ARV1 homolog (yeast) (Arv1), mRNA.	-5.84	-2.55	0.008040877	68865	NM_026855.3	NM_026855.3
ILMN_2666279	Arrdc3	arrestin domain containing 3	-5.72	-2.52	0.006675651		NM_178917.2	NM_178917.2
ILMN_1221526	LOC100041516	similar to 4933409K07Rik protein (LOC100041516), misc RNA.	-5.72	-2.52	0.001565787	100041516	XR_031127.1	XR_031127.1
ILMN_2892292	Alg9	asparagine-linked glycosylation 9 homolog (yeast, alpha 1,2 mannosyltransferase) (Alg9)	-5.59	-2.48	0.002861	102580	NM_133981.1	NM_133981.1
ILMN_2942353	Ccl25	chemokine (C-C motif) ligand 25 (Ccl25), mRNA.	-5.47	-2.45	0.009236907	20300	NM_009138.1	NM_009138.1
ILMN_2703061	2810408P10Rik	RIKEN cDNA 2810408P10 gene (2810408P10Rik), mRNA.	-5.45	-2.45	0.001573	242747	NM_198619.2	NM_198619.2
ILMN_1228242	Zfp383	zinc finger protein 383 (Zfp383), mRNA.	-5.26	-2.40	0.004065645	73729	XM_001003466.1	XM_001003466.1
ILMN_2502542	Uap1	UDP-N-acetylglucosamine pyrophosphorylase 1 (Uap1), mRNA.	-5.20	-2.38	0.004694872	107652	NM_133806.4	NM_133806.4
ILMN_1255006	Rpe	ribulose-5-phosphate-3-epimerase (Rpe), mRNA.	-5.15	-2.37	0.006622	66646	NM_025683.2	NM_025683.2
ILMN_2687140	B3galt6	UDP-Gal:betaGal beta 1,3-galactosyltransferase, polypeptide 6 (B3galt6)	-5.15	-2.36	0.003444308	117592	NM_080445.4	NM_080445.4
ILMN_2800380	Trim9	tripartite motif protein 9 (Trim9), mRNA.	-5.11	-2.35	0.009063	94090	NM_053167.1	NM_053167.1
ILMN_3150536	1200015F23Rik	RIKEN cDNA 1200015F23 gene (1200015F23Rik), mRNA. XM_924991	-5.09	-2.35	0.003881	67809	NM_001033136.2	NM_001033136.2
ILMN_1241137	Taf6	TAF6 RNA polymerase II, TATA box binding protein (TBP)-associated factor (Taf6)	-4.98	-2.32	0.010848	21343	NM_009315.3	NM_009315.3
ILMN_1247404	B230312I18Rik	RIKEN cDNA B230312I18 gene, transcript variant 3 (B230312I18Rik)	-4.98	-2.31	0.005843534	233058	XM_001002154.1	XM_001002154.1
ILMN_1219670	Gpatch4	G patch domain containing 4 (Gpatch4), mRNA.	-4.86	-2.28	0.011304034	66614	NM_025663.2	NM_025663.2
ILMN_1219686	Esd	esterase D/formylglutathione hydrolase (Esd), mRNA.	-4.86	-2.28	0.001566	13885	NM_016903.4	NM_016903.4
ILMN_2543507	1190007I07Rik	RIKEN cDNA 1190007I07 gene (1190007I07Rik), mRNA.	-4.73	-2.24	0.008099381	544717	XM_985039.1	XM_985039.1
ILMN_3049465	D830030K20Rik	RIKEN cDNA D830030K20 gene (D830030K20Rik), mRNA.	-4.72	-2.24	0.003881	320333	NM_177135.2	NM_177135.2

Supplementary Table S2. Continued…

ILMN_1214139	Fmn2	formin 2	-4.51	-2.17	0.002782		AK013585	
ILMN_1233531	2610528E23Rik	RIKEN cDNA 2610528E23 gene (2610528E23Rik), mRNA.	-4.49	-2.17	0.011326	66497	NM_025599.2	NM_025599.2
ILMN_1214850	Pak1	p21 (CDKN1A)-activated kinase 1 (Pak1), mRNA.	-4.46	-2.16	0.001565787	18479	NM_011035.2	NM_011035.2
ILMN_1255422	Ccrn4l	CCR4 carbon catabolite repression 4-like (S. cerevisiae) (Ccrn4l)	-4.42	-2.14	0.001755	12457	NM_009834.1	NM_009834.1
ILMN_2701304	Ube2cbp	ubiquitin-conjugating enzyme E2C binding protein (Ube2cbp), mRNA.	-4.42	-2.14	0.009270558	70348	NM_027394.2	NM_027394.2
ILMN_1214511	Ern2	endoplasmic reticulum (ER) to nucleus signalling 2 (Ern2), mRNA.	-4.38	-2.13	0.022901023	26918	NM_012016.2	NM_012016.2
ILMN_1259641	LOC674912	similar to melanoma antigen, transcript variant 1 (LOC674912), mRNA	-4.33	-2.11	0.043918	674912	XM_979860.1	XM_979860.1
ILMN_1241363	Anxa6	annexin A6	-4.10	-2.04	0.012617388		AK013026	
ILMN_1216231	Scoc	short coiled-coil protein (Scoc), transcript variant 1, mRNA.	-4.10	-2.04	0.024591	56367	NM_001039137.2	NM_001039137.2
ILMN_2604226	Sema5a	sema domain, seven thrombospondin repeats (type 1 and type 1-like), (semaphorin) 5A (Sema5a)	-4.09	-2.03	0.005770	20356	NM_009154.2	NM_009154.2
ILMN_3133817	Arhgap12	Rho GTPase activating protein 12 (Arhgap12), transcript variant 2	-4.07	-2.03	0.035264	75415	NM_029277.2	NM_029277.2
ILMN_2728516	Psmc4	proteasome (prosome, macropain) 26S subunit, ATPase, 4	-4.03	-2.01	0.008145653		XM_355872.1	XM_355872.1
ILMN_2764047	Hmgcll1	3-hydroxymethyl-3-methylglutaryl-Coenzyme A lyase-like 1 (Hmgcll1)	-3.93	-1.97	0.004346	208982	NM_173731.2	NM_173731.2
ILMN_1247036	Ttc3	tetratricopeptide repeat domain 3 (Ttc3), mRNA.	-3.91	-1.97	0.011301	22129	NM_009441.2	NM_009441.2
ILMN_1234842	Msi2h	Msi2, musashi RNA-binding protein 2	-3.88	-1.96	0.014884465		AK051269	
ILMN_2814350	Mkks	McKusick-Kaufman syndrome protein (Mkks), mRNA.	-3.86	-1.95	0.006979737	59030	NM_021527.1	NM_021527.1
ILMN_2518483	Pla2g12a	phospholipase A2, group XIIA	-3.86	-1.95	0.019092		NM_023196	
ILMN_2998738	EG381438	predicted gene, EG381438 (EG381438), mRNA.	-3.82	-1.93	0.009200	381438	NM_198657.1	NM_198657.1
ILMN_2558387	Kcnq5	potassium voltage-gated channel, subfamily Q, member 5	-3.78	-1.92	0.025546		AK039046	
ILMN_2482572	Flnb	filamin, beta (Flnb), mRNA. XM_904364 XM_990154	-3.75	-1.91	0.008630	286940	NM_134080.1	NM_134080.1
ILMN_1214375	Gpn2	GPN-loop GTPase 2 (Gpn2), mRNA.	-3.74	-1.90	0.020629	100210	NM_133884.1	NM_133884.1
ILMN_2826826	Rtbdn	retbindin (Rtbdn), mRNA.	-3.71	-1.89	0.04443638	234542	NM_144929.2	NM_144929.2
ILMN_1229553	Igsf3	immunoglobulin superfamily, member 3 (Igsf3), mRNA.	-3.67	-1.88	0.019093713	78908	NM_207205.1	NM_207205.1
ILMN_1248465	Zcchc3	zinc finger, CCHC domain containing 3 (Zcchc3), mRNA.	-3.66	-1.87	0.016949	67917	NM_175126.3	NM_175126.3
ILMN_2773540	Thap4	THAP domain containing 4 (Thap4), mRNA.	-3.65	-1.87	0.019837	67026	NM_025920.3	NM_025920.3
ILMN_1222402	Pla2g4b	phospholipase A2, group IVB (cytosolic)	-3.52	-1.82	0.008146		XM_358347.1	XM_358347.1
ILMN_1245711	C030034I22Rik	RIKEN cDNA C030034I22 gene (C030034I22Rik), mRNA.	-3.51	-1.81	0.009237526	77533	XM_001478807.1	XM_001478807.1
ILMN_1214422	2810408P10Rik	RIKEN cDNA 2810408P10 gene (2810408P10Rik), mRNA.	-3.49	-1.80	0.014357	242747	NM_198619.2	NM_198619.2
ILMN_2441501	Clstn1	calsyntenin 1	-3.46	-1.79	0.004541		NM_023051	
ILMN_1237919	Ahcy	S-adenosylhomocysteine hydrolase	-3.46	-1.79	0.004330		NM_016661	
ILMN_2819558	Bach2	BTB and CNC homology 2 (Bach2), mRNA.	-3.44	-1.78	0.011300852	12014	NM_007521.2	NM_007521.2
ILMN_2890496	Rbbp9	retinoblastoma binding protein 9 (Rbbp9), mRNA.	-3.44	-1.78	0.00875329	26450	NM_015754.2	NM_015754.2
ILMN_2510187	Slc39a2	solute carrier family 39 (zinc transporter), member 2, transcript variant 2 (Slc39a2)	-3.41	-1.77	0.011301	214922	XM_989872.1	XM_989872.1
ILMN_1232668	Mad	Mxd1, MAX dimerization protein 1	-3.37	-1.75	0.02062865		NM_010751	
ILMN_2723346	1700029J07Rik	RIKEN cDNA 1700029J07 gene (1700029J07Rik), mRNA.	-3.35	-1.74	0.030302377	69479	NM_001033148.2	NM_001033148.2
ILMN_1236666	Acsl6	acyl-CoA synthetase long-chain family member 6 (Acsl6), transcript variant 4	-3.34	-1.74	0.012574284	216739	NM_001033599.1	NM_001033599.1
ILMN_1255053	Plk1	polo-like kinase 1 (Drosophila) (Plk1), mRNA.	-3.27	-1.71	0.011441	18817	NM_011121.3	NM_011121.3
ILMN_1240178	Ccndbp1	cyclin D-type binding-protein 1 (Ccndbp1), mRNA.	-3.26	-1.71	0.003487045	17151	NM_010761.2	NM_010761.2
ILMN_2690256	Insig2	insulin induced gene 2 (Insig2), mRNA.	-3.24	-1.70	0.001565787	72999	NM_133748.1	NM_133748.1
ILMN_1260038	Eif4e	eukaryotic translation initiation factor 4E (Eif4e), mRNA.	-3.23	-1.69	0.011973972	13684	NM_007917.3	NM_007917.3
ILMN_1230157	Rnd3	Rho family GTPase 3 (Rnd3), mRNA.	-3.21	-1.68	0.009814727	74194	NM_028810.2	NM_028810.2
ILMN_2550240	Gtpbp2	GTP binding protein 2	-3.19	-1.67	0.019094		AK008731	
ILMN_1223045	2810417H13Rik	RIKEN cDNA 2810417H13 gene (2810417H13Rik), mRNA.	-3.10	-1.63	0.018774	68026	NM_026515.2	NM_026515.2
ILMN_2475271	Trappc5	trafficking protein particle complex 5 (Trappc5), mRNA.	-3.09	-1.63	0.010446396	66682	NM_025701.3	NM_025701.3
ILMN_1236868	Idb2	Id2, inhibitor of DNA binding 2	-3.05	-1.61	0.007111		AK013239	
ILMN_2507331	Xylt1	xylosyltransferase 1 (Xylt1), mRNA.	-3.03	-1.60	0.008814	233781	NM_175645.3	NM_175645.3
ILMN_2603081	Nt5c3	5'-nucleotidase, cytosolic III (Nt5c3), mRNA.	-3.03	-1.60	0.001566	107569	NM_026004.2	NM_026004.2
ILMN_2657728	Stxbp2	syntaxin binding protein 2	-3.01	-1.59	0.024225084		NM_011503.2	NM_011503.2
ILMN_2722129	Hps1	Hermansky-Pudlak syndrome 1 homolog (human) (Hps1), mRNA.	-3.01	-1.59	0.009236907	192236	NM_019424.2	NM_019424.2
ILMN_2690014	Syt4	synaptotagmin IV (Syt4), mRNA.	-3.00	-1.59	0.039135	20983	NM_009308.3	NM_009308.3
ILMN_2598402	Parp8	poly (ADP-ribose) polymerase family, member 8 (Parp8), mRNA.	-2.99	-1.58	0.006144	52552	NM_001081009.1	NM_001081009.1
ILMN_2588199	Mrps27	mitochondrial ribosomal protein S27 (Mrps27), nuclear gene encoding mitochondrial protein	-2.98	-1.58	0.009281348	218506	NM_173757.3	NM_173757.3
ILMN_2860964	Med23	mediator complex subunit 23 (Med23), mRNA.	-2.98	-1.58	0.017100014	70208	NM_027347.2	NM_027347.2
ILMN_1250939	Agpat5	1-acylglycerol-3-phosphate O-acyltransferase 5 (lysophosphatidic acid acyltransferase, epsilon) (Agpat5)	-2.98	-1.57	0.024591194	52123	NM_026792.3	NM_026792.3
ILMN_1234412	LOC674427	similar to ribosomal protein L7a (LOC674427), misc RNA.	-2.97	-1.57	0.036241	674427	XR_030796.1	XR_030796.1
ILMN_2599861	Defb11	defensin beta 11 (Defb11), mRNA.	-2.91	-1.54	0.012574	246081	NM_139221.1	NM_139221.1
ILMN_2687011	Krt1-12	Krt12, keratin 12	-2.88	-1.53	0.007288		NM_010661.1	NM_010661.1
ILMN_2736042	Cdh6	cadherin 6 (Cdh6), mRNA.	-2.87	-1.52	0.008145653	12563	NM_007666.3	NM_007666.3
ILMN_2620942	Trim62	tripartite motif-containing 62 (Trim62), mRNA.	-2.83	-1.50	0.012070	67525	NM_178110.2	NM_178110.2
ILMN_3006575	6330503K22Rik	RIKEN cDNA 6330503K22 gene (6330503K22Rik), mRNA.	-2.82	-1.49	0.003881	101565	NM_182995.1	NM_182995.1
ILMN_1223318	4833420G17Rik	RIKEN cDNA 4833420G17 gene (4833420G17Rik), mRNA.	-2.82	-1.49	0.009509529	67392	NM_026127.3	NM_026127.3
ILMN_2747430	Atp7a	ATPase, Cu++ transporting, alpha polypeptide (Atp7a), mRNA.	-2.80	-1.49	0.009271	11977	NM_009726.3	NM_009726.3
ILMN_1220121	Kif23	kinesin family member 23	-2.78	-1.48	0.017585		NM_024245	
ILMN_1242402	Npas3	neuronal PAS domain protein 3	-2.78	-1.47	0.022621		AK050957	
ILMN_1236107	Ogfrl1	opioid growth factor receptor-like 1 (Ogfrl1), mRNA.	-2.78	-1.47	0.036472224	70155	XM_973033.1	XM_973033.1
ILMN_2733330	Rps3a	ribosomal protein S3a (Rps3a), mRNA.	-2.76	-1.47	0.011574917	20091	NM_016959.2	NM_016959.2
ILMN_1217776	Mll5	Kmt2e, lysine (K)-specific methyltransferase 2E	-2.76	-1.46	0.045897		XM_485570	
ILMN_1252898	Sox6	SRY (sex determining region Y)-box 6	-2.76	-1.46	0.010236		AK084290	
ILMN_2435505	Usf1	upstream transcription factor 1 (Usf1), mRNA.	-2.75	-1.46	0.025546	22278	NM_009480.2	NM_009480.2
ILMN_2628567	Phlda3	pleckstrin homology-like domain, family A, member 3 (Phlda3), mRNA.	-2.74	-1.45	0.006810	27280	NM_013750.1	NM_013750.1
ILMN_2717176	Rgl1	ral guanine nucleotide dissociation stimulator,-like 1 (Rgl1), mRNA.	-2.72	-1.45	0.018774	19731	NM_016846.3	NM_016846.3

Supplementary Table S2. Continued…

ILMN_1250569	Rapgefl1	Rap guanine nucleotide exchange factor (GEF)-like 1 (Rapgefl1), mRNA	-2.72	-1.44	0.04732002	268480	NM_001080925.1	NM_001080925.1
ILMN_1219305	Gstm6	glutathione S-transferase, mu 6 (Gstm6), mRNA.	-2.70	-1.43	0.008814	14867	NM_008184.3	NM_008184.3
ILMN_1238215	Ctgf	connective tissue growth factor	-2.68	-1.42	0.015210		NM_010217	
ILMN_2602151	Rpap1	RNA polymerase II associated protein 1 (Rpap1), mRNA.	-2.67	-1.42	0.036369	68925	NM_177294.4	NM_177294.4
ILMN_2517290	Impact	imprinted and ancient (Impact), mRNA.	-2.67	-1.41	0.011974	16210	NM_008378.2	NM_008378.2
ILMN_2600315	Ercc8	excision repaiross-complementing rodent repair deficiency, complementation group 8 (Ercc8)	-2.65	-1.41	0.016949	71991	NM_028042.3	NM_028042.3
ILMN_2598576	Parp2	poly (ADP-ribose) polymerase family, member 2 (Parp2), mRNA.	-2.60	-1.38	0.006174062	11546	NM_009632.2	NM_009632.2
ILMN_1246132	E230029C05Rik	RIKEN cDNA E230029C05 gene (E230029C05Rik), mRNA.	-2.60	-1.38	0.008072	319711	XM_001473432.1	XM_001473432.1
ILMN_1251041	Btrc	beta-transducin repeat containing protein (Btrc), transcript variant 1	-2.59	-1.37	0.027877253	12234	NM_001037758.1	NM_001037758.1
ILMN_2747480	Srpr	signal recognition particle receptor ('docking protein') (Srpr), mRN	-2.58	-1.37	0.040968	67398	NM_026130.1	NM_026130.1
ILMN_2865527	Krt12	keratin 12 (Krt12), mRNA.	-2.58	-1.37	0.025546	268482	NM_010661.2	NM_010661.2
ILMN_2807229	Ccdc88c	coiled-coil domain containing 88C (Ccdc88c), mRNA.	-2.57	-1.36	0.006458	68339	NM_026681.4	NM_026681.4
ILMN_2717621	Rps15a	ribosomal protein S15a (Rps15a), mRNA.	-2.55	-1.35	0.044441078	267019	NM_170669.2	NM_170669.2
ILMN_2970834	Pex19	peroxisome biogenesis factor 19 (Pex19), mRNA.	-2.54	-1.34	0.035691973	19298	NM_023041.2	NM_023041.2
ILMN_1253316	Polr3f	polymerase (RNA) III (DNA directed) polypeptide F	-2.53	-1.34	0.015477458		NM_027417	
ILMN_2693858	D14Ertd449e	DNA segment, Chr 14, ERATO Doi 449, expressed (D14Ertd449e), mRNA.	-2.53	-1.34	0.023300	66039	NM_025311.1	NM_025311.1
ILMN_1230048	Fxr2	fragile X mental retardation, autosomal homolog 2 (Fxr2), mRNA.	-2.52	-1.33	0.027203253	23879	NM_011814.2	NM_011814.2
ILMN_1226111	LOC100043821	hypothetical protein LOC100043821 (LOC100043821), mRNA.	-2.51	-1.33	0.002860728	100043821	XM_001481017.1	XM_001481017.1
ILMN_1257622	Tubgcp4	tubulin, gamma complex associated protein 4 (Tubgcp4), mRNA.	-2.51	-1.33	0.008072	51885	NM_153387.2	NM_153387.2
ILMN_2867013	Zfp160	zinc finger protein 160 (Zfp160), mRNA.	-2.50	-1.32	0.009200	224585	NM_145483.1	NM_145483.1
ILMN_1214469	Slc38a6	solute carrier family 38, member 6 (Slc38a6), mRNA.	-2.49	-1.31	0.018471537	625098	XM_916892.2	XM_916892.2
ILMN_2672353	Dennd1c	DENN/MADD domain containing 1C (Dennd1c), mRNA.	-2.48	-1.31	0.044142	70785	NM_153551.1	NM_153551.1
ILMN_3132361	Ncoa4	nuclear receptor coactivator 4 (Ncoa4), transcript variant 2, mRNA.	-2.48	-1.31	0.036375	27057	NM_001033988.1	NM_001033988.1
ILMN_2773909	1600014C10Rik	RIKEN cDNA 1600014C10 gene (1600014C10Rik), transcript variant 2, mR	-2.47	-1.30	0.019091547	72244	NM_028166.3	NM_028166.3
ILMN_1234100	Bsdc1	BSD domain containing 1 (Bsdc1), mRNA.	-2.45	-1.29	0.020975	100383	NM_133889.2	NM_133889.2
ILMN_2498787	Zfp236	zinc finger protein 236, transcript variant 1 (Zfp236), mRNA.	-2.44	-1.29	0.001873293	329002	XM_484752.5	XM_484752.5
ILMN_2660803	Bckdhb	branched chain ketoacid dehydrogenase E1, beta polypeptide (Bckdhb), nuclear gene encoding mitochondrial protein	-2.44	-1.29	0.019186	12040	NM_199195.1	NM_199195.1
ILMN_1255939	Cdh4	cadherin 4 (Cdh4), mRNA.	-2.44	-1.28	0.013099109	12561	NM_009867.1	NM_009867.1
ILMN_2459211	Dgkh	diacylglycerol kinase, eta	-2.44	-1.28	0.024151135		XM_484397	
ILMN_2654906	Mgat3	mannoside acetylglucosaminyltransferase 3 (Mgat3), mRNA.	-2.42	-1.27	0.015193409	17309	NM_010795.3	NM_010795.3
ILMN_1221160	Unc13c	unc-13 homolog C (C. elegans) (Unc13c), mRNA.	-2.37	-1.25	0.033326	208898	NM_001081153.1	NM_001081153.1
ILMN_2944843	LOC545056	ubiquitin-conjugating enzyme E2, J2 homolog pseudogene (LOC545056) on chromosome 14.	-2.37	-1.24	0.010775	545056	NR_002889.1	NR_002889.1
ILMN_3011719	6430706D22Rik	RIKEN cDNA 6430706D22 gene (6430706D22Rik), mRNA.	-2.36	-1.24	0.011952	381280	NM_198652.1	NM_198652.1
ILMN_2960263	Slc8a2	solute carrier family 8 (sodium/calcium exchanger), member 2 (Slc8a2)	-2.36	-1.24	0.043918	110891	NM_148946.2	NM_148946.2
ILMN_1232989	Ehd3	EH-domain containing 3 (Ehd3), mRNA.	-2.34	-1.23	0.024591194	57440	NM_020578.2	NM_020578.2
ILMN_3048728	Centg1	centaurin, gamma 1 (Centg1), mRNA.	-2.33	-1.22	0.03636904	216439	NM_001033263.1	NM_001033263.1
ILMN_2744731	Csnrp3	cysteine-serine-rich nuclear protein 3 (Csnrp3), mRNA.	-2.33	-1.22	0.011974	77771	NM_153409.3	NM_153409.3
ILMN_1257178	Zfp367	zinc finger protein 367 (Zfp367), mRNA.	-2.32	-1.21	0.027931567	238673	NM_175494.4	NM_175494.4
ILMN_2747754	Pygb	brain glycogen phosphorylase (Pygb), mRNA.	-2.32	-1.21	0.015057101	110078	NM_153781.1	NM_153781.1
ILMN_2638958	Cacybp	calcyclin binding protein (Cacybp), mRNA.	-2.31	-1.21	0.004785	12301	NM_009786.1	NM_009786.1
ILMN_1253848	Neo1	neogenin	-2.31	-1.21	0.011301		AK077830	
ILMN_1255657	Yipf4	Yip1 domain family, member 4 (Yipf4), mRNA.	-2.30	-1.20	0.015346	67864	NM_026417.3	NM_026417.3
ILMN_2883164	Serpine2	serine (or cysteine) peptidase inhibitor, clade E, member 2 (Serpine2)	-2.29	-1.19	0.039944235	20720	NM_009255.2	NM_009255.2
ILMN_2440033	Pcm1	pericentriolar material 1	-2.29	-1.19	0.040968		NM_023662	
ILMN_2598239	Stk25	serine/threonine kinase 25 (yeast) (Stk25), mRNA.	-2.27	-1.18	0.011326	59041	NM_021537.3	NM_021537.3
ILMN_2776952	Tmem55b	transmembrane protein 55b (Tmem55b), mRNA. XM_919952 XM_919965	-2.27	-1.18	0.010503	219024	NM_001033271.3	NM_001033271.3
ILMN_1232341	A730008H23Rik	RIKEN cDNA A730008H23 gene (A730008H23Rik), mRNA.	-2.27	-1.18	0.032688152	212427	NM_172505.1	NM_172505.1
ILMN_2714415	4922501C03Rik	RIKEN cDNA 4922501C03 gene (4922501C03Rik), mRNA.	-2.25	-1.17	0.001566	382090	NM_199316.2	NM_199316.2
ILMN_2879759	Tagap	T-cell activation Rho GTPase-activating protein (Tagap), mRNA.	-2.24	-1.16	0.033941	72536	NM_145968.1	NM_145968.1
ILMN_2601928	Pnmal1	PNMA-like 1 (Pnmal1), mRNA.	-2.24	-1.16	0.009236907	71691	NM_001007569.1	NM_001007569.1
ILMN_2903698	Snapc3	small nuclear RNA activating complex, polypeptide 3 (Snapc3), mRNA.	-2.22	-1.15	0.011700333	77634	NM_029949.1	NM_029949.1
ILMN_2968211	Lgals4	lectin, galactose binding, soluble 4 (Lgals4), mRNA.	-2.22	-1.15	0.019290105	16855	NM_010706.1	NM_010706.1
ILMN_3060512	Sel1l	sel-1 suppressor of lin-12-like (C. elegans) (Sel1l), transcript variant 1	-2.20	-1.13	0.024225	20338	NM_001039089.1	NM_001039089.1
ILMN_2636403	Axud1	AXIN1 up-regulated 1 (Axud1), mRNA.	-2.19	-1.13	0.041449	215418	NM_153287.3	NM_153287.3
ILMN_2703585	Gbas	glioblastoma amplified sequence (Gbas), mRNA.	-2.16	-1.11	0.045266	14467	NM_008095.3	NM_008095.3
ILMN_1223868	Aim2	absent in melanoma 2 (Aim2), mRNA.	-2.16	-1.11	0.017100	383619	NM_001013779.1	NM_001013779.1
ILMN_1246870	Spata5l1	spermatogenesis associated 5-like 1 (Spata5l1), mRNA.	-2.16	-1.11	0.02611614	214616	XM_001481333.1	XM_001481333.1
ILMN_2676512	Riok1	RIO kinase 1 (yeast) (Riok1), mRNA.	-2.15	-1.11	0.019836556	71340	NM_024242.2	NM_024242.2
ILMN_2886468	Fbxo46	F-box protein 46 (Fbxo46), mRNA.	-2.15	-1.10	0.011151539	243867	NM_175530.2	NM_175530.2
ILMN_1239859	Nrxn1	neurexin I (Nrxn1), mRNA.	-2.15	-1.10	0.04352716	18189	NM_020252.2	NM_020252.2
ILMN_2503166	C330011F01Rik	RIKEN cDNA C330011F01 gene (C330011F01Rik), mRNA.	-2.14	-1.10	0.038814	78605	XM_001005388.1	XM_001005388.1
ILMN_2920541	Tbc1d9	TBC1 domain family, member 9 (Tbc1d9), mRNA.	-2.14	-1.10	0.012428658	71310	NM_027758.1	NM_027758.1
ILMN_1221822	Rbm12	RNA binding motif protein 12 (Rbm12), transcript variant 2, mRNA.	-2.14	-1.09	0.029402	75710	NM_170598.2	NM_170598.2
ILMN_3135697	1200003I07Rik	RIKEN cDNA 1200003I07 gene (1200003I07Rik), transcript variant 3, mR	-2.12	-1.09	0.001880773	66869	NM_181274.3	NM_181274.3
ILMN_1214255	Cd200	CD200 antigen (Cd200), mRNA.	-2.12	-1.08	0.039606	17470	NM_010818.3	NM_010818.3
ILMN_2674602	Rapgef5	Rap guanine nucleotide exchange factor (GEF) 5 (Rapgef5), mRNA.	-2.12	-1.08	0.042466	217944	NM_175930.4	NM_175930.4
ILMN_2602257	Tmem68	transmembrane protein 68 (Tmem68), mRNA.	-2.12	-1.08	0.005440894	72098	NM_028097.3	NM_028097.3
ILMN_2702857	Osbpl1a	oxysterol binding protein-like 1A	-2.11	-1.08	0.015166		NM_020573.1	NM_020573.1
ILMN_2654651	Thbs3	thrombospondin 3 (Thbs3), mRNA.	-2.11	-1.07	0.044510	21827	NM_013691.1	NM_013691.1
ILMN_1255776	BC017643	cDNA sequence BC017643 (BC017643), mRNA.	-2.10	-1.07	0.04077382	217370	NM_144832.1	NM_144832.1
ILMN_1252850	Rbm8a	RNA binding motif protein 8a (Rbm8a), mRNA.	-2.09	-1.07	0.037917413	60365	NM_025875.1	NM_025875.1

Supplementary Table S2. Continued…

ILMN_1242935	LOC633016	similar to Chromobox homolog 3 (HP1 gamma homolog, Drosophila), transcript variant 2 (LOC633016)	-2.09	-1.06	0.018510	633016	XM_921371.2	XM_921371.2
ILMN_1216136	Cbfa2t3h	core-binding factor, runt domain, alpha subunit 2, translocated to, 3 homolog (human) (Cbfa2t3h)	-2.09	-1.06	0.009611	12398	NM_009824.1	NM_009824.1
ILMN_3097950	Asb1	ankyrin repeat and SOCS box-containing 1 (Asb1), transcript variant 2	-2.09	-1.06	0.02938169	65247	NM_023046.4	NM_023046.4
ILMN_1213448	LOC669658	similar to melanoma antigen (LOC669658), mRNA.	-2.08	-1.06	0.014251053	669658	XM_976371.1	XM_976371.1
ILMN_1236437	B230311B06Rik	RIKEN cDNA B230311B06 gene (B230311B06Rik), mRNA.	-2.08	-1.06	0.038486	381914	XM_001001884.2	XM_001001884.2
ILMN_2425028	LOC675572	hypothetical LOC675572 (LOC675572), mRNA.	-2.08	-1.05	0.010784	675572	XM_001481199.1	XM_001481199.1
ILMN_1213124	Rrp7a	ribosomal RNA processing 7 homolog A (S. cerevisiae) (Rrp7a), mRNA.	-2.08	-1.05	0.043918	74778	NM_029101.3	NM_029101.3
ILMN_1229343	Mboat2	membrane bound O-acyltransferase domain containing 2 (Mboat2), transcript variant 2	-2.07	-1.05	0.033636715	67216	NM_001083341.1	NM_001083341.1
ILMN_3163567	Slc45a1	solute carrier family 45, member 1 (Slc45a1), mRNA.	-2.07	-1.05	0.023251	242773	NM_173774.3	NM_173774.3
ILMN_1218536	Cops5	COP9 signalosome subunit 5	-2.06	-1.04	0.014579		AK005113	
ILMN_2777722	Jarid1b	Kdm5b, lysine (K)-specific demethylase 5B	-2.06	-1.04	0.021900		NM_152895	
ILMN_2740869	Rims2	regulating synaptic membrane exocytosis 2 (Rims2), mRNA.	-2.05	-1.04	0.022901	116838	NM_053271.1	NM_053271.1
ILMN_2613469	Psmb5	proteasome (prosome, macropain) subunit, beta type 5 (Psmb5), mRNA.	-2.04	-1.03	0.036984	19173	NM_011186.1	NM_011186.1
ILMN_1246775	Col1a1	collagen, type I, alpha 1	-2.04	-1.03	0.035021875		AK086730	
ILMN_2769261	Ercc5	excision repair cross-complementing rodent repair deficiency, complementation group 5 (Ercc5)	-2.04	-1.03	0.03292831	22592	NM_011729.1	NM_011729.1
ILMN_2751492	Frag1	FGF receptor activating protein 1 (Frag1), mRNA.	-2.03	-1.02	0.017100014	233575	NM_145583.2	NM_145583.2
ILMN_2645460	2410146L05Rik	RIKEN cDNA 2410146L05 gene (2410146L05Rik), mRNA.	-2.03	-1.02	0.015193409	67968	NM_026480.2	NM_026480.2
ILMN_1252444	Usp53	ubiquitin specific peptidase 53 (Usp53), mRNA.	-2.03	-1.02	0.026692433	99526	NM_133857.3	NM_133857.3
ILMN_1239463	Snf1lk	SNF1-like kinase (Snf1lk), mRNA.	-2.02	-1.01	0.015057101	17691	NM_010831.2	NM_010831.2
ILMN_2875251	Ang	angiogenin, ribonuclease, RNase A family, 5 (Ang), mRNA.	-2.01	-1.01	0.039579	11727	NM_007447.2	NM_007447.2
ILMN_1226082	Mtap2	Map2, microtubule-associated protein 2	-2.01	-1.01	0.010423		AK079618	
ILMN_1218543	2310051N18Rik	RIKEN cDNA 2310051N18 gene, transcript variant 1 (2310051N18Rik)	-1.99	-1.00	0.04054272	71941	XM_977376.1	XM_977376.1
ILMN_2672035	Ccdc115	coiled-coil domain containing 115 (Ccdc115), mRNA.	-1.99	-0.99	0.049468	69668	NM_027159.1	NM_027159.1
ILMN_2587786	Fbxo3	F-box protein 3 (Fbxo3), transcript variant 2, mRNA.	-1.98	-0.98	0.03636904	57443	NM_020593.2	NM_020593.2
ILMN_1257771	LOC638301	similar to interferon activated gene 204 (LOC638301), mRNA.	-1.97	-0.97	0.007288012	638301	XM_914287.2	XM_914287.2
ILMN_2710159	MGC41689	Plac9a, placenta specific 9a	-1.96	-0.97	0.042650655		NM_207229	
ILMN_2783896	Atm	ataxia telangiectasia mutated homolog (human) (Atm), mRNA.	-1.95	-0.96	0.040856265	11920	NM_007499.1	NM_007499.1
ILMN_1250852	Ccdc5	coiled-coil domain containing 5 (Ccdc5), mRNA.	-1.95	-0.96	0.025546	225745	NM_146089.2	NM_146089.2
ILMN_1221985	1700123A16Rik	RIKEN cDNA 1700123A16 gene (1700123A16Rik), mRNA.	-1.95	-0.96	0.036506344	73610	XM_984400.1	XM_984400.1
ILMN_1218037	Tmie	transmembrane inner ear (Tmie), mRNA.	-1.94	-0.96	0.040363636	20776	NM_146260.2	NM_146260.2
ILMN_2717716	Rapgef5	Rap guanine nucleotide exchange factor (GEF) 5	-1.94	-0.96	0.031119		NM_175930	
ILMN_2741872	Heatr5a	HEAT repeat containing 5A (Heatr5a), mRNA.	-1.94	-0.95	0.019092	320487	NM_177171.4	NM_177171.4
ILMN_1248753	Grm5	glutamate receptor, metabotropic 5 (Grm5), mRNA.	-1.94	-0.95	0.031842	108071	NM_001081414.1	NM_001081414.1
ILMN_1244060	Pik3r4	phosphatidylinositol 3 kinase, regulatory subunit, polypeptide 4, p150, transcript variant 1 (Pik3r4)	-1.93	-0.95	0.034820	75669	XM_135116.7	XM_135116.7
ILMN_2524980	H2afj	H2A histone family, member J (H2afj), mRNA.	-1.92	-0.94	0.02545933	232440	NM_177688.4	NM_177688.4
ILMN_1259294	Tmem126b	transmembrane protein 126B (Tmem126b), mRNA.	-1.92	-0.94	0.030595	68472	NM_026734.1	NM_026734.1
ILMN_2897020	Dhrsx	dehydrogenase/reductase (SDR family) X chromosome (Dhrsx), mRNA.	-1.92	-0.94	0.016949	236082	NM_001033326.2	NM_001033326.2
ILMN_2739816	AW822252	expressed sequence AW822252 (AW822252), mRNA.	-1.91	-0.93	0.025545875	331578	XM_001474711.1	XM_001474711.1
ILMN_1227126	Ppp2r3a	protein phosphatase 2 (formerly 2A), regulatory subunit B'', alpha (Ppp2r3a)	-1.91	-0.93	0.009510	19054	XM_001471965.1	XM_001471965.1
ILMN_1254717	Zfp429	zinc finger protein 429 (Zfp429), mRNA.	-1.90	-0.93	0.015057	72807	NM_001080941.1	NM_001080941.1
ILMN_2686353	Sult2b1	sulfotransferase family, cytosolic, 2B, member 1 (Sult2b1), mRNA.	-1.90	-0.93	0.024591	54200	NM_017465.1	NM_017465.1
ILMN_2651556	Otof	otoferlin (Otof), transcript variant 2, mRNA.	-1.90	-0.93	0.046356	83762	NM_031875.2	NM_031875.2
ILMN_3004949	D930028F11Rik	RIKEN cDNA D930028F11 gene (D930028F11Rik), mRNA.	-1.88	-0.91	0.007083731	244853	NM_172921.2	NM_172921.2
ILMN_2606711	LOC100046163	similar to Nme6 protein (LOC100046163), mRNA.	-1.87	-0.90	0.019591	100046163	XM_001475710.1	XM_001475710.1
ILMN_1255148	H13	histocompatibility 13	-1.86	-0.89	0.042244		AK039008	
ILMN_2775098	Cyb5	cytochrome b-5 (Cyb5), mRNA.	-1.86	-0.89	0.033128966	109672	NM_025797.3	NM_025797.3
ILMN_2754253	Gna13	guanine nucleotide binding protein, alpha 13	-1.85	-0.89	0.029743174		NM_010303.2	NM_010303.2
ILMN_2668849	Prkcbp1	protein kinase C binding protein 1 (Prkcbp1), mRNA.	-1.84	-0.88	0.004177	228880	NM_027230.3	NM_027230.3
ILMN_1230688	Ilf2	interleukin enhancer binding factor 2	-1.83	-0.87	0.006979737		AK031892	
ILMN_3112363	Kcnj6	potassium inwardly-rectifying channel, subfamily J, member 6 (Kcnj6), transcript variant Girk2B	-1.83	-0.87	0.028130135	16522	NM_001025585.2	NM_001025585.2
ILMN_2654068	Med23	mediator complex subunit 23 (Med23), mRNA.	-1.82	-0.87	0.03406417	70208	NM_027347.2	NM_027347.2
ILMN_1250138	Muc1	mucin 1, transmembrane (Muc1), mRNA.	-1.82	-0.87	0.009570435	17829	NM_013605.1	NM_013605.1
ILMN_1247561	Pdhb	pyruvate dehydrogenase (lipoamide) beta	-1.81	-0.86	0.011974		AK084507	
ILMN_2626300	Rbm12	RNA binding motif protein 12	-1.81	-0.85	0.03943855		NM_029397.2	NM_029397.2
ILMN_1246321	Gstm6	glutathione S-transferase, mu 6	-1.80	-0.85	0.036369		NM_008184.1	NM_008184.1
ILMN_2761205	Hspb8	heat shock protein 8 (Hspb8), mRNA.	-1.80	-0.85	0.044510	80888	NM_030704.1	NM_030704.1
ILMN_2867158	3110007F17Rik	RIKEN cDNA 3110007F17 gene (3110007F17Rik), mRNA.	-1.79	-0.84	0.011126042	73061	NM_028426.1	NM_028426.1
ILMN_2781353	Ush2a	Usher syndrome 2A (autosomal recessive, mild) homolog (human) (Ush2a)	-1.78	-0.84	0.034064	22283	NM_021408.2	NM_021408.2
ILMN_2848548	Pts	6-pyruvoyl-tetrahydropterin synthase (Pts), mRNA.	-1.77	-0.83	0.045043	19286	NM_011220.2	NM_011220.2
ILMN_2497745	Mtap9	microtubule-associated protein 9 (Mtap9), mRNA.	-1.77	-0.82	0.023751	213582	NM_001081230.1	NM_001081230.1
ILMN_2862706	H2-Q5	histocompatibility 2, Q region locus 5 (H2-Q5), mRNA.	-1.77	-0.82	0.01126063	15016	NM_010393.1	NM_010393.1
ILMN_2562131	Fmo1	flavin containing monooxygenase 1	-1.77	-0.82	0.020421		AK042457	
ILMN_2703182	Lgals7	lectin, galactose binding, soluble 7 (Lgals7), mRNA.	-1.76	-0.82	0.033968	16858	NM_008496.4	NM_008496.4
ILMN_2851251	Hn1l	hematological and neurological expressed 1-like (Hn1l), mRNA.	-1.76	-0.82	0.008814	52009	NM_198937.2	NM_198937.2
ILMN_1257649	Zfp236	zinc finger protein 236, transcript variant 1 (Zfp236), mRNA.	-1.76	-0.81	0.03229423	329002	XM_484752.5	XM_484752.5
ILMN_1253819	Prkar2b	protein kinase, cAMP dependent regulatory, type II beta (Prkar2b), m	-1.75	-0.81	0.04352716	19088	NM_011158.3	NM_011158.3
ILMN_2580180	LOC100039514	similar to gag protein (LOC100039514), mRNA.	-1.75	-0.81	0.043918	100039514	XM_001473142.1	XM_001473142.1
ILMN_2668835	Npas2	neuronal PAS domain protein 2 (Npas2), mRNA.	-1.75	-0.81	0.034730	18143	NM_008719.1	NM_008719.1
ILMN_3135409	Elmo2	engulfment and cell motility 2, ced-12 homolog (C. elegans) (Elmo2), transcript variant 1	-1.75	-0.80	0.029331	140579	NM_207705.1	NM_207705.1
ILMN_2501267	Tmc7	transmembrane channel-like gene family 7 (Tmc7), mRNA.	-1.72	-0.78	0.031842183	209760	NM_172476.4	NM_172476.4
ILMN_2519789	Zfp101	zinc finger protein 101 (Zfp101), mRNA.	-1.71	-0.77	0.009597	22643	NM_009542.2	NM_009542.2

Supplementary Table S2. Continued…

ILMN_1216709	Pbx4	pre B cell leukemia homeobox 4	-1.71	-0.77	0.043652		NM_030555	
ILMN_1252144	Mif4gd	MIF4G domain containing (Mif4gd), mRNA.	-1.71	-0.77	0.017497	69674	NM_027162.3	NM_027162.3
ILMN_3058975	Fnta	farnesyltransferase, CAAX box, alpha (Fnta), mRNA.	-1.71	-0.77	0.047248047	14272	NM_008033.2	NM_008033.2
ILMN_3090545	Dppa5	developmental pluripotency associated 5 (Dppa5), mRNA.	-1.71	-0.77	0.033488423	434423	NM_025274.1	NM_025274.1
ILMN_1252865	Zfp46	zinc finger protein 46	-1.70	-0.77	0.009648		AK051031	
ILMN_1250182	Cep164	centrosomal protein 164 (Cep164), mRNA.	-1.70	-0.76	0.021340	214552	NM_001081373.1	NM_001081373.1
ILMN_2803286	Tex9	testis expressed gene 9 (Tex9), mRNA.	-1.69	-0.76	0.021365	21778	NM_009359.2	NM_009359.2
ILMN_2621921	Clcn7	chloride channel 7 (Clcn7), mRNA.	-1.69	-0.76	0.014969261	26373	NM_011930.3	NM_011930.3
ILMN_1241225	Dctd	dCMP deaminase (Dctd), mRNA.	-1.69	-0.75	0.024591194	320685	NM_178788.3	NM_178788.3
ILMN_2840247	Zfp157	zinc finger protein 157 (Zfp157), mRNA.	-1.68	-0.75	0.010121631	72154	NM_028130.2	NM_028130.2
ILMN_2840958	Thbs3	thrombospondin 3 (Thbs3), mRNA.	-1.67	-0.74	0.024591	21827	NM_013691.1	NM_013691.1
ILMN_1256991	Stk3	serine/threonine kinase 3	-1.67	-0.74	0.036603		AK084333	
ILMN_2711754	Foxred1	FAD-dependent oxidoreductase domain containing 1 (Foxred1), mRNA.	-1.66	-0.73	0.023661984	235169	NM_172291.1	NM_172291.1
ILMN_3059476	Sesn1	sestrin 1 (Sesn1), mRNA.	-1.66	-0.73	0.030745	140742	NM_001013370.1	NM_001013370.1
ILMN_1247094	Nrp	neural regeneration protein	-1.66	-0.73	0.020253		AK030358	
ILMN_2596998	Lypd6b	LY6/PLAUR domain containing 6B (Lypd6b), mRNA.	-1.66	-0.73	0.049089342	71897	NM_027990.3	NM_027990.3
ILMN_1218584	LOC100044172	hypothetical protein LOC100044172 (LOC100044172), mRNA.	-1.65	-0.72	0.043943	100044172	XM_001471720.1	XM_001471720.1
ILMN_2713898	Ddr1	discoidin domain receptor family, member 1 (Ddr1), transcript variant 1	-1.65	-0.72	0.030316	12305	NM_007584.2	NM_007584.2
ILMN_1237736	Plekhf2	pleckstrin homology domain containing, family F (with FYVE domain) member 2 (Plekhf2)	-1.64	-0.72	0.022362	71801	NM_175175.3	NM_175175.3
ILMN_1213855	Extl2	exostoses (multiple)-like 2 (Extl2), mRNA.	-1.64	-0.71	0.039236	58193	NM_021388.3	NM_021388.3
ILMN_2777019	Spo11	sporulation protein, meiosis-specific, SPO11 homolog (S. cerevisiae) (Spo11), transcript variant 1	-1.64	-0.71	0.0480551	26972	NM_012046.2	NM_012046.2
ILMN_2601515	Bpnt1	bisphosphate 3'-nucleotidase 1	-1.64	-0.71	0.041141		NM_011794.2	NM_011794.2
ILMN_1257336	Cwf19l1	CWF19-like 1, cell cycle control (S. pombe) (Cwf19l1), mRNA.	-1.64	-0.71	0.027735153	72502	NM_001081077.1	NM_001081077.1
ILMN_3001398	Sall3	sal-like 3 (Drosophila) (Sall3), mRNA.	-1.63	-0.71	0.034481	20689	NM_178280.3	NM_178280.3
ILMN_2593993	Eif4el3	Eif4e2, eukaryotic translation initiation factor 4E member 2	-1.63	-0.71	0.014817		NM_023314.2	NM_023314.2
ILMN_2951446	Ypel5	yippee-like 5 (Drosophila) (Ypel5), mRNA.	-1.63	-0.71	0.045266	383295	NM_027166.3	NM_027166.3
ILMN_2460491	Slc39a9	solute carrier family 39 (zinc transporter), member 9 (Slc39a9)	-1.63	-0.71	0.009597168	328133	XM_992396.1	XM_992396.1
ILMN_1236139	Myef2	myelin basic protein expression factor 2, repressor (Myef2), mRNA.	-1.62	-0.70	0.043918	17876	NM_010852.1	NM_010852.1
ILMN_2777706	Mrps12	mitochondrial ribosomal protein S12	-1.62	-0.70	0.039236		NM_011885	
ILMN_2868188	Usp10	ubiquitin specific peptidase 10 (Usp10), mRNA.	-1.62	-0.69	0.019094	22224	NM_009462.1	NM_009462.1
ILMN_2456243	Zfp148	zinc finger protein 148 (Zfp148), mRNA.	-1.62	-0.69	0.017983304	22661	NM_011749.4	NM_011749.4
ILMN_2666380	Spag1	sperm associated antigen 1 (Spag1), mRNA.	-1.62	-0.69	0.040233	26942	NM_012031.1	NM_012031.1
ILMN_2952661	Dbf4	DBF4 homolog (S. cerevisiae) (Dbf4), mRNA.	-1.61	-0.69	0.032269	27214	NM_013726.2	NM_013726.2
ILMN_1250075	Dpysl3	dihydropyrimidinase-like 3 (Dpysl3), mRNA.	-1.61	-0.69	0.018093	22240	NM_009468.3	NM_009468.3
ILMN_3160963	AI316807	expressed sequence AI316807 (AI316807), mRNA.	-1.61	-0.69	0.032248124	102032	NM_001012667.1	NM_001012667.1
ILMN_1237963	Alg8	asparagine-linked glycosylation 8 homolog (yeast, alpha-1,3-glucosyltransferase) (Alg8)	-1.61	-0.68	0.008146	381903	NM_199035.1	NM_199035.1
ILMN_1254218	Nisch	nischarin	-1.60	-0.68	0.006809535		AK036043	
ILMN_1254734	Mrps12	mitochondrial ribosomal protein S12 (Mrps12), mRNA.	-1.60	-0.68	0.034820	24030	NM_011885.3	NM_011885.3
ILMN_1239863	E330016A19Rik	RIKEN cDNA E330016A19 gene (E330016A19Rik), mRNA.	-1.60	-0.68	0.008145653	214763	NM_173386.3	NM_173386.3
ILMN_1258517	Gbe1	glucan (1,4-alpha-), branching enzyme 1	-1.60	-0.68	0.035391994		AK050423	
ILMN_1256245	Rlf	rearranged L-myc fusion sequence, transcript variant 3 (Rlf), mRNA.	-1.59	-0.67	0.008823	109263	XM_900557.2	XM_900557.2
ILMN_1232132	EG667705	predicted gene, EG667705 (EG667705), mRNA.	-1.59	-0.67	0.042244	667705	XM_001475654.1	XM_001475654.1
ILMN_2683222	Srd5a3	steroid 5 alpha-reductase 3 (Srd5a3), mRNA.	-1.56	-0.64	0.018774	57357	NM_020611.3	NM_020611.3
ILMN_2660864	Klhl7	kelch-like 7 (Drosophila) (Klhl7), mRNA.	-1.56	-0.64	0.003420	52323	NM_026448.2	NM_026448.2
ILMN_2939012	1500012F01Rik	RIKEN cDNA 1500012F01 gene (1500012F01Rik), mRNA.	-1.55	-0.63	0.038415	68949	NM_001081005.1	NM_001081005.1
ILMN_1252089	Nfatc3	nuclear factor of activated T cells, cytoplasmic, calcineurin dependent 3	-1.55	-0.63	0.04180203		AK084848	
ILMN_2672325	LOC100046163	similar to Nme6 protein (LOC100046163), mRNA.	-1.55	-0.63	0.027877	100046163	XM_001475710.1	XM_001475710.1
ILMN_2653402	Vps13b	vacuolar protein sorting 13B (yeast), transcript variant 5 (Vps13b)	-1.54	-0.62	0.045363	666173	XM_981774.2	XM_981774.2
ILMN_2632509	Ppp1r14c	protein phosphatase 1, regulatory (inhibitor) subunit 14c (Ppp1r14c)	-1.53	-0.61	0.042436	76142	NM_133485.2	NM_133485.2
ILMN_2475184	Taf6	TAF6 RNA polymerase II, TATA box binding protein (TBP)-associated factor (Taf6)	-1.52	-0.61	0.035640	21343	NM_009315.3	NM_009315.3
ILMN_2645276	Spsb3	splA/ryanodine receptor domain and SOCS box containing 3 (Spsb3), mR	-1.52	-0.60	0.044510	79043	NM_027141.1	NM_027141.1
ILMN_1228489	Hmg20a	high mobility group 20A	-1.52	-0.60	0.044521		AK017716	
ILMN_2878430	Rpl30	ribosomal protein L30 (Rpl30), mRNA.	-1.51	-0.60	0.03994772	19946	NM_009083.2	NM_009083.2
ILMN_2650392	Rbm28	RNA binding motif protein 28 (Rbm28), transcript variant 2, mRNA.	-1.51	-0.60	0.027877	68272	NM_133925.1	NM_133925.1
ILMN_1231066	Hist2h2be	histone cluster 2, H2be (Hist2h2be), mRNA.	-1.50	-0.59	0.033512	319190	NM_178214.3	NM_178214.3
ILMN_2454325	3200001K10Rik	RIKEN cDNA 3200001K10 gene, transcript variant 1 (3200001K10Rik), m	-1.50	-0.59	0.048926	381626	XM_988683.1	XM_988683.1
ILMN_3136656	Nrp2	neuropilin 2 (Nrp2), transcript variant 5, mRNA.	1.50	0.59	0.043918	18187	NM_001077406.1	NM_001077406.1
ILMN_2710604	Dbnl	drebrin-like (Dbnl), mRNA.	1.50	0.59	0.030491142	13169	NM_013810.2	NM_013810.2
ILMN_1212649	Gcc2	GRIP and coiled-coil domain containing 2 (Gcc2), mRNA.	1.51	0.59	0.020754682	70297	NM_027375.2	NM_027375.2
ILMN_1234204	Gm1060	gene model 1060, (NCBI) (Gm1060), mRNA.	1.51	0.59	0.045819	381738	NM_001033460.2	NM_001033460.2
ILMN_1257702	Lars	leucyl-tRNA synthetase (Lars), mRNA.	1.51	0.60	0.032920	107045	NM_134137.2	NM_134137.2
ILMN_2770917	Blvrb	biliverdin reductase B (flavin reductase (NADPH))	1.51	0.60	0.012348		NM_144923	
ILMN_2673776	E2f2	E2F transcription factor 2 (E2f2), mRNA.	1.52	0.60	0.021846129	242705	NM_177733.2	NM_177733.2
ILMN_2928875	Atp6v1g1	ATPase, H+ transporting, lysosomal V1 subunit G1 (Atp6v1g1), mRNA.	1.52	0.61	0.020552	66290	NM_024173.1	NM_024173.1
ILMN_2854858	Zfp161	zinc finger protein 161 (Zfp161), mRNA.	1.53	0.62	0.019738	22666	NM_009547.2	NM_009547.2
ILMN_1227907	Gmfg	glia maturation factor, gamma (Gmfg), transcript variant 1, mRNA.	1.54	0.62	0.035692	63986	NM_022024.2	NM_022024.2
ILMN_1219563	Gprasp1	G protein-coupled receptor associated sorting protein 1 (Gprasp1), transcript variant 3, mRNA.	1.54	0.62	0.023152	67298	NM_001005385.1	NM_001005385.1
ILMN_2876325	Fbxo34	F-box protein 34 (Fbxo34), mRNA.	1.54	0.62	0.044510	78938	NM_030236.1	NM_030236.1
ILMN_1252821	3110035E14Rik	RIKEN cDNA 3110035E14 gene (3110035E14Rik), mRNA.	1.55	0.63	0.043918	76982	NM_178399.4	NM_178399.4
ILMN_2851330	Nagk	N-acetylglucosamine kinase (Nagk), mRNA.	1.55	0.63	0.01675983	56174	NM_019542.1	NM_019542.1
ILMN_1245119	Pcdh12	protocadherin 12 (Pcdh12), mRNA.	1.55	0.63	0.046257753	53601	NM_017378.2	NM_017378.2

Supplementary Table S2. Continued…

ILMN_2788036	Acsbg1	acyl-CoA synthetase bubblegum family member 1 (Acsbg1), mRNA.	1.55	0.63	0.021846129	94180	NM_053178.1	NM_053178.1
ILMN_2903926	Pdgfrl	platelet-derived growth factor receptor-like (Pdgfrl), mRNA.	1.55	0.63	0.028059	68797	NM_026840.2	NM_026840.2
ILMN_2481771	Nr1i3	nuclear receptor subfamily 1, group I, member 3 (Nr1i3), mRNA.	1.55	0.63	0.011300852	12355	NM_009803.4	NM_009803.4
ILMN_2865558	Vps33a	vacuolar protein sorting 33A (yeast) (Vps33a), mRNA.	1.55	0.63	0.012916	77573	NM_029929.2	NM_029929.2
ILMN_1243170	Ubiad1	UbiA prenyltransferase domain containing 1 (Ubiad1), mRNA.	1.56	0.64	0.048232593	71707	NM_027873.2	NM_027873.2
ILMN_2578829	Slc11a2	solute carrier family 11 (proton-coupled divalent metal ion transporters), member 2	1.56	0.64	0.005667		AK083478	
ILMN_2590638	Bcl2l11	BCL2-like 11 (apoptosis facilitator)	1.56	0.64	0.034233		NM_009754.2	NM_009754.2
ILMN_1230457	Cd99l2	CD99 antigen-like 2 (Cd99l2), mRNA.	1.56	0.64	0.048888	171486	NM_138309.2	NM_138309.2
ILMN_2707198	As3mt	arsenic (+3 oxidation state) methyltransferase	1.56	0.65	0.023368		NM_020577.1	NM_020577.1
ILMN_2640188	Taf1a	TATA-box binding protein associated factor, RNA polymerase I, A	1.56	0.65	0.034729872		NM_021466.1	NM_021466.1
ILMN_1241482	LOC100039607	hypothetical protein LOC100039607 (LOC100039607), mRNA.	1.57	0.65	0.028423578	100039607	XM_001473184.1	XM_001473184.1
ILMN_1226311	Chd9	chromodomain helicase DNA binding protein 9	1.57	0.65	0.015057		XM_284439	
ILMN_1215768	Riok3	RIO kinase 3 (yeast) (Riok3), mRNA.	1.57	0.65	0.025546	66878	NM_024182.4	NM_024182.4
ILMN_2628271	LOC100045967	hypothetical protein LOC100045967 (LOC100045967), misc RNA.	1.57	0.65	0.032248124	100045967	XR_032154.1	XR_032154.1
ILMN_2653765	Nusap1	nucleolar and spindle associated protein 1 (Nusap1), transcript variant 2	1.57	0.65	0.036472	108907	NM_001042652.1	NM_001042652.1
ILMN_1221564	Ltbp1	latent transforming growth factor beta binding protein 1 (Ltbp1), transcript variant 2, mRNA.	1.57	0.65	0.007288012	268977	NM_206958.1	NM_206958.1
ILMN_2801540	Chchd5	coiled-coil-helix-coiled-coil-helix domain containing 5 (Chchd5), mR	1.57	0.65	0.046356	66170	NM_025395.1	NM_025395.1
ILMN_1251142	3110052M02Rik	RIKEN cDNA 3110052M02 gene, transcript variant 2 (3110052M02Rik), m	1.57	0.66	0.049089342	73229	XM_976578.1	XM_976578.1
ILMN_2537089	4933402J24Rik	RIKEN cDNA 4933402J24 gene, transcript variant 3 (4933402J24Rik), m	1.58	0.66	0.027914697	74438	XM_622899.3	XM_622899.3
ILMN_2872419	Zbtb8os	zinc finger and BTB domain containing 8 opposite strand (Zbtb8os), m	1.58	0.66	0.045846	67106	NM_025970.1	NM_025970.1
ILMN_2694074	Gins4	GINS complex subunit 4 (Sld5 homolog) (Gins4), mRNA.	1.58	0.66	0.042797513	109145	NM_024240.3	NM_024240.3
ILMN_2815383	Srfbp1	serum response factor binding protein 1 (Srfbp1), mRNA.	1.59	0.67	0.005844	67222	NM_026040.2	NM_026040.2
ILMN_1213886	Meox1	mesenchyme homeobox 1 (Meox1), mRNA.	1.59	0.67	0.035644	17285	NM_010791.3	NM_010791.3
ILMN_2983686	Trub2	TruB pseudouridine (psi) synthase homolog 2 (E. coli) (Trub2), mRNA.	1.59	0.67	0.009271	227682	NM_145520.2	NM_145520.2
ILMN_2692762	Ap4m1	adaptor-related protein complex AP-4, mu 1 (Ap4m1), mRNA.	1.60	0.68	0.004230	11781	NM_021392.3	NM_021392.3
ILMN_2981801	Hist1h2ag	histone cluster 1, H2ag (Hist1h2ag), mRNA.	1.61	0.69	0.04596005	319167	NM_178186.2	NM_178186.2
ILMN_1260389	Lin7b	lin-7 homolog B (C. elegans) (Lin7b), mRNA.	1.61	0.69	0.029743	22342	NM_011698.1	NM_011698.1
ILMN_2618794	Ptcd2	pentatricopeptide repeat domain 2	1.61	0.69	0.026115		XM_127497.2	XM_127497.2
ILMN_2497410	Pcdhb6	protocadherin beta 6	1.62	0.70	0.018774		NM_053131	
ILMN_2683856	Noxo1	NADPH oxidase organizer 1 (Noxo1), mRNA.	1.62	0.70	0.033512	71893	NM_027988.1	NM_027988.1
ILMN_1251018	Fxyd2	FXYD domain-containing ion transport regulator 2 (Fxyd2), transcript variant b	1.63	0.70	0.021318	11936	NM_052823.2	NM_052823.2
ILMN_2911283	Ap2s1	adaptor-related protein complex 2, sigma 1 subunit (Ap2s1), mRNA.	1.63	0.71	0.038619	232910	NM_198613.1	NM_198613.1
ILMN_3133085	Zfp68	zinc finger protein 68 (Zfp68), transcript variant 2, mRNA.	1.63	0.71	0.038813885	24135	NM_001044747.1	NM_001044747.1
ILMN_3102072	Srrm2	serine/arginine repetitive matrix 2 (Srrm2), mRNA.	1.63	0.71	0.02315249	75956	NM_175229.3	NM_175229.3
ILMN_2918002	Gbp3	guanylate nucleotide binding protein 3 (Gbp3), mRNA.	1.65	0.72	0.040856	55932	NM_018734.2	NM_018734.2
ILMN_2972249	4930555G01Rik	RIKEN cDNA 4930555G01 gene (4930555G01Rik), mRNA.	1.65	0.72	0.02315249	108978	NM_175393.2	NM_175393.2
ILMN_2738629	Acot8	acyl-CoA thioesterase 8 (Acot8), mRNA.	1.65	0.72	0.011426305	170789	NM_133240.1	NM_133240.1
ILMN_1227086	Pik3cg	phosphoinositide-3-kinase, catalytic, gamma polypeptide (Pik3cg), mR	1.66	0.73	0.028806	30955	NM_020272.1	NM_020272.1
ILMN_2754561	A430033K04Rik	RIKEN cDNA A430033K04 gene (A430033K04Rik), mRNA.	1.66	0.73	0.042088	243308	NM_183025.1	NM_183025.1
ILMN_2608025	Palm2	paralemmin 2 (Palm2), mRNA.	1.66	0.73	0.03209266	242481	NM_172868.2	NM_172868.2
ILMN_2628281	Scly	selenocysteine lyase (Scly), mRNA.	1.67	0.74	0.001566	50880	NM_016717.3	NM_016717.3
ILMN_2969314	Mtrr	5-methyltetrahydrofolate-homocysteine methyltransferase reductase (Mtrr)	1.68	0.74	0.038415	210009	NM_172480.1	NM_172480.1
ILMN_1229544	LOC100041569	hypothetical protein LOC100041569 (LOC100041569), mRNA.	1.68	0.75	0.044201	100041569	XM_001476596.1	XM_001476596.1
ILMN_1223261	Mkrn1	makorin, ring finger protein, 1	1.68	0.75	0.017694		AK005137	
ILMN_1242946	LOC629364	similar to actin related protein 2/3 complex, subunit 5 (LOC629364)	1.68	0.75	0.005914	629364	XM_001479142.1	XM_001479142.1
ILMN_2707291	Prdm2	PR domain containing 2, with ZNF domain (Prdm2), mRNA.	1.68	0.75	0.005155001	110593	NM_001081355.1	NM_001081355.1
ILMN_1244310	Gm106	gene model 106, (NCBI) (Gm106), mRNA.	1.68	0.75	0.031842183	226866	NM_001033288.3	NM_001033288.3
ILMN_2639155	Prcp	prolylcarboxypeptidase (angiotensinase C) (Prcp), mRNA.	1.68	0.75	0.014737642	72461	NM_028243.2	NM_028243.2
ILMN_1250422	Lhx6	LIM homeobox protein 6	1.69	0.75	0.040219		AK090339	
ILMN_2535238	Xkr4	X Kell blood group precursor related family member 4 (Xkr4), mRNA.	1.69	0.76	0.023294	497097	NM_001011874.1	NM_001011874.1
ILMN_3060788	9030025P20Rik	RIKEN cDNA 9030025P20 gene (9030025P20Rik), transcript variant 2, mR	1.69	0.76	0.04180203	381062	NM_001034891.2	NM_001034891.2
ILMN_2543456	D2Bwg1335e	DNA segment, Chr 2, Brigham & Women's Genetics 1335 expressed (D2Bwg1335e)	1.70	0.77	0.035692	52838	NM_026828.2	NM_026828.2
ILMN_3001341	Cnnm1	cyclin M1 (Cnnm1), mRNA.	1.71	0.77	0.039579	83674	NM_031396.1	NM_031396.1
ILMN_1221817	Cd74	CD74 antigen (invariant polypeptide of major histocompatibility complex, class II antigen-associated) (Cd74)	1.71	0.77	0.045043	16149	NM_001042605.1	NM_001042605.1
ILMN_3155626	EG245297	predicted gene, EG245297 (EG245297), mRNA.	1.72	0.78	0.047022	245297	NM_001018086.2	NM_001018086.2
ILMN_2737129	Klf12	Kruppel-like factor 12	1.72	0.79	0.038438		NM_010636	
ILMN_1213470	Ugcgl2	UDP-glucose ceramide glucosyltransferase-like 2 (Ugcgl2), mRNA.	1.72	0.79	0.001565787	66435	NM_001081252.1	NM_001081252.1
ILMN_2862111	Hmgn2	high mobility group nucleosomal binding domain 2 (Hmgn2), mRNA.	1.73	0.79	0.044510	15331	NM_016957.3	NM_016957.3
ILMN_1250014	Crmp5-pending	Dpysl5, dihydropyrimidinase-like 5	1.74	0.80	0.038911156		AK082132	
ILMN_2629805	Epha3	Eph receptor A3 (Epha3), mRNA.	1.75	0.80	0.042798	13837	NM_010140.3	NM_010140.3
ILMN_1220583	2610109H07Rik	RIKEN cDNA 2610109H07 gene (2610109H07Rik), mRNA.	1.75	0.81	0.04612947	70433	NM_027426.2	NM_027426.2
ILMN_1218127	Pcp4l1	Purkinje cell protein 4-like 1 (Pcp4l1), mRNA.	1.75	0.81	0.03447103	66425	XM_484933.5	XM_484933.5
ILMN_3143557	Adi1	acireductone dioxygenase 1 (Adi1), mRNA.	1.76	0.81	0.025676811	104923	NM_134052.2	NM_134052.2
ILMN_1235196	Atp10d	ATPase, Class V, type 10D (Atp10d), mRNA.	1.77	0.82	0.004066	231287	NM_153389.2	NM_153389.2
ILMN_1232969	Bxdc2	brix domain containing 2 (Bxdc2), mRNA.	1.77	0.82	0.035608	67832	NM_026396.3	NM_026396.3
ILMN_3086899	Tcte3	t-complex-associated testis expressed 3 (Tcte3), transcript variant 2	1.78	0.83	0.040233	21647	NM_198104.2	NM_198104.2
ILMN_2826110	Cat	catalase (Cat), mRNA.	1.78	0.83	0.024082	12359	NM_009804.1	NM_009804.1
ILMN_1220357	Zfp536	zinc finger protein 536	1.79	0.84	0.006458		NM_172385	
ILMN_1249743	LOC100048638	similar to lens fiber cell beaded-filament structure protein (LOC100048638), misc RNA.	1.79	0.84	0.02466517	100048638	XR_034832.1	XR_034832.1
ILMN_2591345	Csnk1g1	casein kinase 1, gamma 1 (Csnk1g1), mRNA.	1.79	0.84	0.041643	214897	NM_173185.2	NM_173185.2
ILMN_2562107	Usp14	ubiquitin specific peptidase 14	1.80	0.85	0.039981518		AK042356	

Supplementary Table S2. Continued…

ILMN_1247765	Mfhas1	malignant fibrous histiocytoma amplified sequence 1 (Mfhas1), mRNA.	1.80	0.85	0.042323	52065	NM_001081279.1	NM_001081279.1
ILMN_1235943	Bptf	bromodomain PHD finger transcription factor (Bptf), mRNA.	1.81	0.85	0.027877253	207165	NM_176850.2	NM_176850.2
ILMN_2879534	Extl1	exostoses (multiple)-like 1 (Extl1), mRNA.	1.81	0.86	0.000813	56219	NM_019578.1	NM_019578.1
ILMN_1237595	Fv1	Friend virus susceptibility 1 (Fv1), mRNA.	1.82	0.86	0.009199905	14349	NM_010244.3	NM_010244.3
ILMN_1220680	Lpin2	lipin 2	1.83	0.87	0.033967786		NM_022882	
ILMN_2661367	Hyou1	hypoxia up-regulated 1 (Hyou1), mRNA.	1.83	0.87	0.036472	12282	NM_021395.2	NM_021395.2
ILMN_3001540	Lum	lumican (Lum), mRNA.	1.85	0.89	0.031331	17022	NM_008524.1	NM_008524.1
ILMN_1256633	LOC100045567	similar to purine nucleoside phosphorylase (LOC100045567), mRNA.	1.89	0.92	0.026578	100045567	XM_001474536.1	XM_001474536.1
ILMN_3162618	Luc7l	Luc7 homolog (S. cerevisiae)-like (Luc7l), transcript variant 2	1.90	0.93	0.015409398	66978	NM_028190.2	NM_028190.2
ILMN_2904703	Ctsl	cathepsin L (Ctsl), mRNA.	1.92	0.94	0.017658675	13039	NM_009984.2	NM_009984.2
ILMN_2607926	Kdelr2	KDEL (Lys-Asp-Glu-Leu) endoplasmic reticulum protein retention receptor 2 (Kdelr2)	1.92	0.94	0.015057	66913	NM_025841.3	NM_025841.3
ILMN_2546510	EG434402	predicted gene, EG434402 (EG434402), mRNA.	1.92	0.94	0.029976	434402	XM_001479948.1	XM_001479948.1
ILMN_2896768	Cbr3	carbonyl reductase 3 (Cbr3), mRNA.	1.93	0.95	0.041802	109857	NM_173047.2	NM_173047.2
ILMN_2655015	Alad	aminolevulinate, delta-, dehydratase (Alad), mRNA.	1.93	0.95	0.035577	17025	NM_008525.3	NM_008525.3
ILMN_1225291	LOC668631	hypothetical protein LOC668631 (LOC668631), mRNA.	1.93	0.95	0.04505528	668631	XR_002223.1	XR_002223.1
ILMN_1221007	Prkd3	protein kinase D3 (Prkd3), mRNA.	1.93	0.95	0.031842	75292	NM_029239.2	NM_029239.2
ILMN_2625114	Cdkal1	CDK5 regulatory subunit associated protein 1-like 1	1.94	0.95	0.031152		NM_144536.1	NM_144536.1
ILMN_3067404	Zfp386	zinc finger protein 386 (Kruppel-like) (Zfp386), transcript variant1	1.95	0.97	0.009736721	56220	NM_001004066.2	NM_001004066.2
ILMN_1226295	Frmd3	FERM domain containing 3	1.97	0.98	0.004258337		NM_172869	
ILMN_2834728	Prkd3	protein kinase D3 (Prkd3), mRNA.	1.97	0.98	0.024225	75292	NM_029239.2	NM_029239.2
ILMN_1230916	LOC433955	similar to HSPC008 (LOC433955), misc RNA.	1.98	0.98	0.003586719	433955	XR_033167.1	XR_033167.1
ILMN_1228540	6430590A10Rik	RIKEN cDNA 6430590A10 gene (6430590A10Rik), misc RNA.	1.98	0.98	0.015057	319850	XR_035434.1	XR_035434.1
ILMN_1248763	Thex1	three prime histone mRNA exonuclease 1 (Thex1), mRNA.	1.98	0.99	0.032062	67276	NM_026067.2	NM_026067.2
ILMN_2694782	2410076I21Rik	RIKEN cDNA 2410076I21 gene, transcript variant 1 (2410076I21Rik), m	1.98	0.99	0.0457604	73673	XM_134948.5	XM_134948.5
ILMN_3022981	LOC545013	hypothetical protein LOC545013 (LOC545013), mRNA.	1.99	0.99	0.036339074	545013	NM_001025085.1	NM_001025085.1
ILMN_2699665	Cd209f	CD209f antigen, transcript variant 1 (Cd209f), mRNA.	2.00	1.00	0.039944235	69142	XM_284386.5	XM_284386.5
ILMN_2525605	EG667728	predicted gene, EG667728 (EG667728), misc RNA.	2.02	1.01	0.022362	667728	XR_035278.1	XR_035278.1
ILMN_2488846	Tmod4	tropomodulin 4 (Tmod4), mRNA.	2.03	1.02	0.024591	50874	NM_016712.2	NM_016712.2
ILMN_1213858	Itgb3bp	integrin beta 3 binding protein (beta3-endonexin) (Itgb3bp), mRNA.	2.03	1.02	0.008629682	67733	NM_026348.3	NM_026348.3
ILMN_2669793	Ccnd1	cyclin D1 (Ccnd1), mRNA.	2.03	1.02	0.028911574	12443	NM_007631.2	NM_007631.2
ILMN_2569127	Ncaml-pending	Igsf9, immunoglobulin superfamily, member 9	2.03	1.02	0.030694		AK053544	
ILMN_2864844	Vps33b	vacuolar protein sorting 33B (yeast) (Vps33b), mRNA.	2.04	1.03	0.043594	233405	NM_178070.2	NM_178070.2
ILMN_3070389	ENSMUSG00000068790	predicted gene, ENSMUSG00000068790 (ENSMUSG00000068790), mRNA.	2.04	1.03	0.045960	545007	NM_001029930.1	NM_001029930.1
ILMN_2667352	Glo1	glyoxalase 1 (Glo1), mRNA.	2.05	1.03	0.011304	109801	NM_025374.2	NM_025374.2
ILMN_2717146	Nphs2	nephrosis 2 homolog, podocin (human) (Nphs2), mRNA.	2.08	1.05	0.045043	170484	NM_130456.3	NM_130456.3
ILMN_2672772	Abhd1	abhydrolase domain containing 1 (Abhd1), transcribed RNA.	2.08	1.05	0.001566	57742	NR_003522.1	NR_003522.1
ILMN_2697790	Fgfr1op	Fgfr1 oncogene partner (Fgfr1op), mRNA.	2.08	1.06	0.039845	75296	NM_201230.4	NM_201230.4
ILMN_2450460	Ube2j2	ubiquitin-conjugating enzyme E2, J2 homolog (yeast) (Ube2j2), transcript variant 3	2.08	1.06	0.025546	140499	NM_001039158.1	NM_001039158.1
ILMN_1251909	Vars2	valyl-tRNA synthetase 2, mitochondrial (putative) (Vars2), mRNA.	2.11	1.08	0.033516	68915	NM_175137.3	NM_175137.3
ILMN_1240677	Gadd45gip1	growth arrest and DNA-damage-inducible, gamma interacting protein 1 (Gadd45gip1)	2.16	1.11	0.047962	102060	NM_183358.3	NM_183358.3
ILMN_2511806	Clec16a	C-type lectin domain family 16, member A (Clec16a), mRNA.	2.17	1.12	0.033968	74374	NM_177562.4	NM_177562.4
ILMN_2745212	Hist1h2ai	histone cluster 1, H2ai (Hist1h2ai), mRNA.	2.17	1.12	0.046572	319191	NM_178182.1	NM_178182.1
ILMN_2958159	Eno1	enolase 1, alpha non-neuron (Eno1), mRNA.	2.19	1.13	0.010234	13806	NM_023119.1	NM_023119.1
ILMN_1255527	Fmr1	fragile X mental retardation syndrome 1	2.19	1.13	0.028911		AK080898	
ILMN_2846297	3110035E14Rik	RIKEN cDNA 3110035E14 gene (3110035E14Rik), mRNA.	2.21	1.14	0.01077485	76982	NM_178399.2	NM_178399.2
ILMN_2752545	Lama3	laminin, alpha 3	2.21	1.14	0.026148		XM_128926.3	XM_128926.3
ILMN_2733852	Gnb1	guanine nucleotide binding protein (G protein), beta 1 (Gnb1), mRNA.	2.23	1.16	0.025546	14688	NM_008142.3	NM_008142.3
ILMN_2841280	Habp4	hyaluronic acid binding protein 4 (Habp4), mRNA.	2.23	1.16	0.006942	56541	NM_019986.1	NM_019986.1
ILMN_2925567	Nubp2	nucleotide binding protein 2 (Nubp2), mRNA.	2.24	1.16	0.039579153	26426	NM_011956.2	NM_011956.2
ILMN_1215964	Lhfpl3	lipoma HMGIC fusion partner-like 3 (Lhfpl3), mRNA.	2.24	1.16	0.026882	269629	NM_001081231.1	NM_001081231.1
ILMN_2674425	Sys1	SYS1 Golgi-localized integral membrane protein homolog (S. cerevisiae) (Sys1)	2.25	1.17	0.02368987	66460	NM_025575.3	NM_025575.3
ILMN_2699307	Hebp1	heme binding protein 1 (Hebp1), mRNA.	2.25	1.17	0.018510	15199	NM_013546.2	NM_013546.2
ILMN_2426079	Gpr137b	G protein-coupled receptor 137B (Gpr137b), mRNA.	2.25	1.17	0.036369	83924	NM_031999.2	NM_031999.2
ILMN_2718406	Rab26	RAB26, member RAS oncogene family, transcript variant 1 (Rab26), mR	2.27	1.18	0.028395	328778	XM_283428.6	XM_283428.6
ILMN_1236500	Glce	glucuronyl C5-epimerase	2.28	1.19	0.022163		NM_033320	
ILMN_1226839	Hist1h2ad	histone cluster 1, H2ad (Hist1h2ad), mRNA.	2.28	1.19	0.039579153	319165	NM_178188.3	NM_178188.3
ILMN_1229634	Usp29	ubiquitin specific peptidase 29 (Usp29), mRNA.	2.31	1.21	0.020754682	57775	NM_021323.2	NM_021323.2
ILMN_2654679	LOC100040671	similar to 1700001E04Rik protein (LOC100040671), misc RNA.	2.32	1.21	0.035384	100040671	XR_032179.1	XR_032179.1
ILMN_3076680	EG245297	predicted gene, EG245297 (EG245297), mRNA.	2.33	1.22	0.004745	245297	NM_001018086.2	NM_001018086.2
ILMN_2658227	Pparbp	Med1, mediator complex subunit 1	2.33	1.22	0.0190119		NM_013634.1	NM_013634.1
ILMN_2847144	Hist1h2ak	histone cluster 1, H2ak (Hist1h2ak), mRNA.	2.36	1.24	0.023690	319169	NM_178183.1	NM_178183.1
ILMN_2730329	Hist1h2ah	histone cluster 1, H2ah (Hist1h2ah), mRNA.	2.36	1.24	0.029402383	319168	NM_175659.1	NM_175659.1
ILMN_2909238	Spnb1	spectrin beta 1 (Spnb1), mRNA.	2.37	1.25	0.032809	20741	NM_013675.3	NM_013675.3
ILMN_1242672	EG434168	predicted gene, EG434168 (EG434168), mRNA.	2.41	1.27	0.010420112	434168	XM_973360.2	XM_973360.2
ILMN_2729289	Matn4	matrilin 4 (Matn4), mRNA.	2.41	1.27	0.009570435	17183	NM_013592.2	NM_013592.2
ILMN_3099758	Pi4k2b	phosphatidylinositol 4-kinase type 2 beta (Pi4k2b), transcript variant 2	2.41	1.27	0.020519	67073	NM_028744.1	NM_028744.1
ILMN_2815889	Grwd1	glutamate-rich WD repeat containing 1 (Grwd1), mRNA.	2.44	1.29	0.008071645	101612	NM_153419.1	NM_153419.1
ILMN_2901363	2310002B06Rik	RIKEN cDNA 2310002B06 gene (2310002B06Rik), mRNA.	2.44	1.29	0.012512	53951	NM_181649.4	NM_181649.4
ILMN_2421925	Wdfy1	WD repeat and FYVE domain containing 1	2.45	1.29	0.021159		NM_027057	
ILMN_2979430	Thg1l	tRNA-histidine guanylyltransferase 1-like (S. cerevisiae) (Thg1l), m	2.47	1.30	0.035094	66628	NM_001080969.1	NM_001080969.1
ILMN_2822850	Serpinh1	serine (or cysteine) peptidase inhibitor, clade H, member 1 (Serpinh1)	2.52	1.33	0.01959061	12406	NM_009825.1	NM_009825.1

Supplementary Table S2. Continued…

ILMN_1221684	Scp2	sterol carrier protein 2, liver (Scp2), mRNA.	2.55	1.35	0.022890	20280	NM_011327.2	NM_011327.2
ILMN_2935012	Anxa4	annexin A4 (Anxa4), mRNA.	2.56	1.36	0.02009626	11746	NM_013471.1	NM_013471.1
ILMN_1233149	Shhrs	distal-less homeobox 6, opposite strand 1	2.58	1.37	0.008146		XM_489124	
ILMN_1215984	Clcn4-2	Clcn4, chloride channel, voltage-sensitive 4	2.61	1.38	0.015166		AK088317	
ILMN_1215660	Dmtf1	cyclin D binding myb-like transcription factor 1	2.64	1.40	0.012699		AK030508	
ILMN_3014529	2610042L04Rik	RIKEN cDNA 2610042L04 gene (2610042L04Rik), mRNA.	2.65	1.41	0.028130	545015	NM_025940.3	NM_025940.3
ILMN_2968123	Slc7a14	solute carrier family 7 (cationic amino acid transporter, y+ system), member 14 (Slc7a14)	2.66	1.41	0.003881	241919	NM_172861.2	NM_172861.2
ILMN_1216690	Samd11	sterile alpha motif domain containing 11 (Samd11), mRNA.	2.71	1.44	0.016949	231004	NM_173736.2	NM_173736.2
ILMN_1243314	LOC622994	hypothetical LOC622994 (LOC622994), misc RNA.	2.71	1.44	0.010848	622994	XR_031862.1	XR_031862.1
ILMN_2644008	Chl1	cell adhesion molecule L1-like	2.71	1.44	0.027646		NM_007697.1	NM_007697.1
ILMN_2881296	Tmem66	transmembrane protein 66 (Tmem66), mRNA.	2.73	1.45	0.029786	67887	NM_026432.2	NM_026432.2
ILMN_1249578	Sorl1	sortilin-related receptor, LDLR class A repeats-containing (Sorl1),	2.75	1.46	0.010775	20660	NM_011436.3	NM_011436.3
ILMN_1237719	Grasp	GRP1 (general receptor for phosphoinositides 1)-associated scaffold protein	2.84	1.50	0.02168166		AK051199	
ILMN_1236799	Ttll2	tubulin tyrosine ligase-like family, member 2 (Ttll2), mRNA.	2.89	1.53	0.011388	625850	NM_001098267.1	NM_001098267.1
ILMN_2634349	Grlf1	glucocorticoid receptor DNA binding factor 1 (Grlf1), mRNA.	2.91	1.54	0.008275	232906	NM_172739.4	NM_172739.4
ILMN_2481836	LOC668047	similar to ring finger protein 170 (LOC668047), mRNA.	2.99	1.58	0.02315249	668047	XM_001001100.1	XM_001001100.1
ILMN_2477213	LOC100045240	hypothetical protein LOC100045240 (LOC100045240), mRNA.	3.00	1.59	0.01412672	100045240	XM_001473915.1	XM_001473915.1
ILMN_3163340	Rbm45	RNA binding motif protein 45 (Rbm45), mRNA.	3.08	1.62	0.005287885	241490	NM_153405.2	NM_153405.2
ILMN_2724055	1700001E04Rik	RIKEN cDNA 1700001E04 gene (1700001E04Rik), mRNA.	3.21	1.68	0.023368089	75438	NM_029288.2	NM_029288.2
ILMN_1225187	LOC675899	similar to H2A histone family, member Z (LOC675899), mRNA.	3.36	1.75	0.023690	675899	XM_985882.1	XM_985882.1
ILMN_1260199	Casc4	cancer susceptibility candidate 4 (Casc4), transcript variant 1	3.41	1.77	0.017259	319996	NM_177054.3	NM_177054.3
ILMN_1228629	Zfp704	zinc finger protein 704 (Zfp704), mRNA.	3.51	1.81	0.032688152	170753	NM_133218.1	NM_133218.1
ILMN_2664909	Mesdc1	mesoderm development candidate 1 (Mesdc1), mRNA.	3.53	1.82	0.002969	80889	NM_030705.4	NM_030705.4
ILMN_1246800	Serpina3n	serine (or cysteine) peptidase inhibitor, clade A, member 3N (Serpina3n)	3.55	1.83	0.032854	20716	NM_009252.2	NM_009252.2
ILMN_1224719	LOC665032	similar to ribosomal protein (LOC665032), mRNA.	3.56	1.83	0.012699	665032	XM_991641.1	XM_991641.1
ILMN_1222253	Neto2	neuropilin (NRP) and tolloid (TLL)-like 2, transcript variant 8 (Neto2)	3.66	1.87	0.010269403	74513	XM_922064.2	XM_922064.2
ILMN_2624176	Nsun2	NOL1/NOP2/Sun domain family member 2 (Nsun2), mRNA.	3.70	1.89	0.003881	28114	NM_145354.3	NM_145354.3
ILMN_1223496	LOC100046025	similar to mKIAA1230 protein, transcript variant 1 (LOC100046025),	3.71	1.89	0.011700333	100046025	XM_001475823.1	XM_001475823.1
ILMN_2752569	Paip1	polyadenylate binding protein-interacting protein 1 (Paip1), transcript variant 1	3.95	1.98	0.018093077	218693	NM_145457.3	NM_145457.3
ILMN_2800466	Tpmt	thiopurine methyltransferase (Tpmt), mRNA.	4.18	2.06	0.003973	22017	NM_016785.1	NM_016785.1
ILMN_2648661	Cap1	CAP, adenylate cyclase-associated protein 1 (yeast) (Cap1), mRNA.	4.38	2.13	0.002860728	12331	NM_007598.2	NM_007598.2
ILMN_1229454	Cuedc1	CUE domain containing 1 (Cuedc1), mRNA.	4.62	2.21	0.002719146	103841	NM_198013.1	NM_198013.1
ILMN_2961165	Ttl	tubulin tyrosine ligase (Ttl), mRNA.	4.81	2.27	0.009237	69737	NM_027192.1	NM_027192.1
ILMN_1240828	LOC100040657	similar to 1700001E04Rik protein (LOC100040657), mRNA.	4.85	2.28	0.001873293	100040657	XM_001475283.1	XM_001475283.1
ILMN_2705097	Deadc1	deaminase domain containing 1 (Deadc1), mRNA.	4.86	2.28	0.002444489	66757	NM_025748.3	NM_025748.3
ILMN_3022092	LOC544988	hypothetical protein LOC544988 (LOC544988), mRNA.	4.87	2.28	0.009597	544988	NM_001024712.1	NM_001024712.1
ILMN_2667463	Ocel1	occludin/ELL domain containing 1 (Ocel1), mRNA.	4.97	2.31	0.009237526	77090	NM_029865.1	NM_029865.1
ILMN_2502614	Ttc15	tetratricopeptide repeat domain 15 (Ttc15), mRNA.	5.17	2.37	0.007288012	217449	NM_178811.3	NM_178811.3
ILMN_2590520	H2-Bl	histocompatibility 2, blastocyst (H2-Bl), mRNA.	5.40	2.43	0.008072	14963	NM_008199.1	NM_008199.1
ILMN_1216142	Masp2	mannan-binding lectin serine peptidase 2 (Masp2), transcript variant 2	5.61	2.49	0.007074	17175	NM_010767.3	NM_010767.3
ILMN_2838965	4930455C21Rik	RIKEN cDNA 4930455C21 gene (4930455C21Rik), mRNA.	5.62	2.49	0.008071645	76916	NM_024273.1	NM_024273.1
ILMN_2969172	Tmem87a	transmembrane protein 87A (Tmem87a), mRNA.	6.67	2.74	0.004541	211499	NM_173734.2	NM_173734.2
ILMN_2559669	Pbx1	pre B cell leukemia homeobox 1	7.60	2.93	0.001873293		AK037006	
ILMN_2604282	Sfrp1	secreted frizzled-related protein 1	7.95	2.99	0.001566		NM_013834.1	NM_013834.1
ILMN_2729513	Hbb-b2	hemoglobin, beta adult minor chain (Hbb-b2), mRNA.	9.11	3.19	0.003881	15130	NM_016956.2	NM_016956.2
ILMN_2728465	Sstr1	somatostatin receptor 1 (Sstr1), mRNA.	9.83	3.30	0.014969	20605	NM_009216.3	NM_009216.3
ILMN_1214408	LOC666403	similar to ribosomal protein S2 (LOC666403), misc RNA.	36.64	5.20	0.002395	666403	XR_034389.1	XR_034389.1

* Fold change in 129 relative to B6

Supplementary Table S3. Differentially expressed genes between WT B6 and nm1054 B6.

Probe ID	Gene Name	Gene Description	Fold Change*	Log FC	p (Corr)	Entrez Gene ID	Accession Number	RefSeq ID
ILMN_1215469	Dbi	diazepam binding inhibitor (Dbi), transcript variant 2, mRNA.	-155.43	-7.28	0.0000000000	13167	NM_007830.3	NM_007830.3

* Fold change in *nm1054* relative to WT

Supplementary Table S4. Differentially expressed genes between WT 129 and nm1054 129.

Probe ID	Gene Name	Gene Description	Fold Change*	Log FC	p (Corr)	Entrez Gene ID	Accession Number	RefSeq ID
ILMN_1215469	Dbi	diazepam binding inhibitor (Dbi), transcript variant 2, mRNA.	-141.75829	-7.147289	0.001210398	13167	NM_007830.3	NM_007830.3

* Fold change in *nm1054* relative to WT

Supplementary Table S5. Genes absent in B6 and present or marginal in 129.

Strain 129		Strain B6							
WT	nm1054	WT	nm1054	Probe ID	Gene Name	Gene Description	Entrez Gene II	Accession Number	RefSeq ID
P	P	A	A	ILMN_2564493	9630038C08Rik			AK036131	
P	P	A	A	ILMN_2844820	Angptl7	Mus musculus angiopoietin-like 7 (Angptl7), mRNA.	654812	NM_001039554.1	NM_001039554.1
P	P	A	A	ILMN_1225440	C430046P22Rik	Whrn, whirlin			
P	P	A	A	ILMN_1241260	D330011G23Rik			AK052232	
P	P	A	A	ILMN_1233293	Gbp1	Mus musculus guanylate binding protein 1 (Gbp1), mRNA.	14468	NM_010259.2	NM_010259.2
P/M	P	A	A	ILMN_1241298	Hal	Mus musculus histidine ammonia lyase (Hal), mRNA.	15109	NM_010401.3	NM_010401.3
P/M	P	A	A	ILMN_2841352	Nos2	Mus musculus nitric oxide synthase 2, inducible, macrophage (Nos2), mRNA.	18126	NM_010927.1	NM_010927.1
P	P	A	A	ILMN_2898886	OTTMUSG00000010673	Mus musculus predicted gene, OTTMUSG00000010673 (OTTMUSG00000010673), mRNA	433804	NM_001014397.1	NM_001014397.1
P/M	P/M	A	A	ILMN_1236032	0610025J13Rik	RIKEN cDNA 0610025J13 gene		XM_355548	
M	P/M	A	A	ILMN_2633926	1110018H23Rik	Ptx4, pentraxin 4		XM_128459.2	XM_128459.2
P/M	P/M	A	A	ILMN_1257831	2310005G13Rik	Mus musculus RIKEN cDNA 2310005G13 gene (2310005G13Rik), mRNA.	69457	NM_183281.1	NM_183281.1
P/M	P/M	A	A	ILMN_2463970	3110052M02Rik	Zfp983, zinc finger protein 983		XM_484601	
	P/M	A	A	ILMN_1229563	4930512B01Rik	RIKEN cDNA 4930512B01 gene		XM_484154	
	P/M	A	A	ILMN_2948344	4930594M22Rik	Mus musculus RIKEN cDNA 4930594M22 gene (4930594M22Rik), mRNA.	654799	NM_001039531.1	NM_001039531.1
P/M	P/M	A	A	ILMN_1253324	5330417K06Rik	Mob3a, MOB kinase activator 3A		NM_172457.1	NM_172457.1
P/M	P/M	A	A	ILMN_1229834	B930088D13Rik			AK047558	
P/M	P/M	A	A	ILMN_1244203	C330020F18Rik			AK049297	
	P/M	A	A	ILMN_1239753	Cd226	Mus musculus CD226 antigen (Cd226), transcript variant 2, mRNA.	225825	NM_001039149.1	NM_001039149.1
	P/M	A	A	ILMN_1240543	Esco1	Mus musculus establishment of cohesion 1 homolog 1 (S. cerevisiae) (Esco1), mRNA.	77805	NM_001081222.1	NM_001081222.1
P/M	P/M	A	A	ILMN_1242928	Gm41	PREDICTED: Mus musculus gene model 41, (NCBI) (Gm41), mRNA.	245502	XM_982501.2	XM_982501.2
	P/M	A	A	ILMN_1234449	Hsd3b5	Mus musculus hydroxy-delta-5-steroid dehydrogenase, 3 beta- and steroid delta-isomerase 5 (Hsd3b5), mRNA.	15496	NM_008295.2	NM_008295.2
	P/M	A	A	ILMN_1216191	Lcp2	lymphocyte cytosolic protein 2		AK037970	
P/M	P/M	A	A	ILMN_1221961	LOC100047693	PREDICTED: Mus musculus similar to cell adhesion molecule nectin-3 beta (LOC100047693), mRNA.	100047693	XM_001478695.1	XM_001478695.1
	P/M	A	A	ILMN_1245187	LOC233437	Vmn2r66, vomeronasal 2, receptor 66		XM_145883.3	XM_145883.3
P/M	P/M	A	A	ILMN_2529219	LOC241901			XM_143135.3	XM_143135.3
M	P/M	A	A	ILMN_2533110	LOC245187			XM_142713.2	XM_142713.2
P/M	P/M	A	A	ILMN_2531455	LOC328949	Mcc, mutated in colorectal cancers		XM_140309.3	XM_140309.3
P/M	P/M	A	A	ILMN_2534365	LOC331762			XM_284940.2	XM_284940.2
	P/M	A	A	ILMN_2540403	LOC381897			XM_355914.1	XM_355914.1
M	P/M	A	A	ILMN_2538876	LOC384311			XM_357560.1	XM_357560.1
P/M	P/M	A	A	ILMN_2537853	LOC385649			XM_358844.1	XM_358844.1
P/M	P/M	A	A	ILMN_1259502	LOC386372			XM_359190.1	XM_359190.1
	P/M	A	A	ILMN_2532295	LOC635720	PREDICTED: Mus musculus similar to odorant binding protein Ib (LOC635720), mRNA.	635720	XM_910856.3	XM_910856.3
P	P/M	A	A	ILMN_1240746	Mcpt4	Mus musculus mast cell protease 4 (Mcpt4), mRNA.	17227	NM_010779.2	NM_010779.2
	P/M	A	A	ILMN_1241965	Mitf	Mus musculus microphthalmia-associated transcription factor (Mitf), mRNA.	17342	NM_008601.2	NM_008601.2
	P/M	A	A	ILMN_1237477	Olfr267	olfactory receptor 267		XM_143799.1	XM_143799.1
M	P/M	A	A	ILMN_1239542	Raet1c	Mus musculus retinoic acid early transcript gamma (Raet1c), mRNA.	19370	NM_009018.1	NM_009018.1
P/M	P/M	A	A	ILMN_2522952	scl0003425.1_488				
	P/M	A	A	ILMN_3161253	Shisa3	Mus musculus shisa homolog 3 (Xenopus laevis) (Shisa3), mRNA.	330096	NM_001033415.2	NM_001033415.2
P	P/M	A	A	ILMN_2474239	Sla2	Src-like-adaptor 2			
P/M	P/M	A	A	ILMN_2760848	Tbx22	Mus musculus T-box 22 (Tbx22), transcript variant 2, mRNA.	245572	NM_181319.2	NM_181319.2
P/M	P/M	A	A	ILMN_2814385	Tlcd2	Mus musculus TLC domain containing 2 (Tlcd2), mRNA.	380712	NM_027249.2	NM_027249.2
	P/M	A	A	ILMN_2613685	Tmc8	Mus musculus transmembrane channel-like gene family 8 (Tmc8), mRNA.	217356	NM_181856.1	NM_181856.1
M	P/M	A	A	ILMN_2473612	Unc93a	Mus musculus unc-93 homolog A (C. elegans) (Unc93a), mRNA.	381058	NM_199252.1	NM_199252.1
	P/M	A	A	ILMN_2516531	Yes	Yes1, YES proto-oncogene 1, Src family tyrosine kinase		NM_009535	
	P/M	A	A	ILMN_2757924	Zpbp2	Mus musculus zona pellucida binding protein 2 (Zpbp2), transcript variant 1, mRNA.	69376	NM_027061.1	NM_027061.1
P/M		A	A	ILMN_2676754	1700042B14Rik	Cldn34b2, claudin 34B2		XM_136155.1	XM_136155.1
P/M		A	A	ILMN_2446908	1700109F18Rik	PREDICTED: Mus musculus RIKEN cDNA 1700109F18 gene (1700109F18Rik), mRNA.	73429	XM_984549.1	XM_984549.1
M		A	A	ILMN_2422077	4933437C06Rik	Atp5l, ATP synthase, H+ transporting, mitochondrial F0 complex, subunit G			
P/M		A	A	ILMN_2690214	5830405N20Rik	Mus musculus RIKEN cDNA 5830405N20 gene (5830405N20Rik), mRNA.	67596	NM_183264.3	NM_183264.3
P		A	A	ILMN_1234381	B230220B15Rik	RIKEN cDNA B230220B15 gene		NM_177246.2	NM_177246.2
M		A	A	ILMN_2993962	BC099439	Mus musculus cDNA sequence BC099439 (BC099439), mRNA.	217066	NM_001025564.1	NM_001025564.1
P/M		A	A	ILMN_2581154	D930007B01Rik			AK086117	
P/M		A	A	ILMN_2419183	Golph4	Golim4, golgi integral membrane protein 4		NM_175193	
P/M		A	A	ILMN_1219819	Lamc3	laminin gamma 3		NM_011836	
M		A	A	ILMN_2531788	LOC213411			XM_124808.2	XM_124808.2
P/M		A	A	ILMN_2528331	LOC213480	Trav7-6, T cell receptor alpha variable 7-6		XM_135569.2	XM_135569.2
P/M		A	A	ILMN_2538843	LOC381729			XM_355706.1	XM_355706.1
P/M		A	A	ILMN_2540329	LOC382246			XM_356362.1	XM_356362.1
P/M		A	A	ILMN_1241220	LOC384019			XM_357372.1	XM_357372.1

Supplementary Table S5. Continued…

P/M	A	A	ILMN_1223450	Nphs2	Mus musculus nephrosis 2 homolog, podocin (human) (Nphs2), mRNA.	170484	NM_130456.3	NM_130456.3
P/M	A	A	ILMN_1242348	Snrpb2	Mus musculus U2 small nuclear ribonucleoprotein B (Snrpb2), mRNA.	20639	NM_021335.3	NM_021335.3
P/M	A	A	ILMN_2482651	Syne2	Mus musculus synaptic nuclear envelope 2 (Syne2), mRNA.	319565	NM_001005510.2	NM_001005510.2

A: absent; M: marginal; P: present. No call is indicated if any of the samples in a sample group failed to pass QC.

Supplementary Table S6. Genes absent in 129 and present or marginal in B6.

Strain 129		Strain B6							
WT	nm1054	WT	nm1054	Probe ID	Gene Name	Gene Description	Entrez Gene II	Accession Number	RefSeq ID
A	A	P/M	P/M	ILMN_1234774	2010005H15Rik	Mus musculus RIKEN cDNA 2010005H15 gene (2010005H15Rik), mRNA.	76770	NM_029733.2	NM_029733.2
A	A	P/M	M	ILMN_2679749	4930415F15Rik	PREDICTED: Mus musculus RIKEN cDNA 4930415F15 gene (4930415F15Rik), mRNA.	73862	XM_203398.3	XM_203398.3
A	A	P/M	P	ILMN_2756396	4930579C15Rik	Mus musculus RIKEN cDNA 4930579C15 gene (4930579C15Rik), mRNA.	67753	NM_027089.3	NM_027089.3
A	A	P/M	P/M	ILMN_1259267	A630032B19Rik			AK041719	
A	A	P/M	P/M	ILMN_2665478	Alox8	Mus musculus arachidonate 8-lipoxygenase (Alox8), mRNA.	11688	NM_009661.3	NM_009661.3
A	A	P/M		ILMN_2751345	Arhgef6	Mus musculus Rac/Cdc42 guanine nucleotide exchange factor (GEF) 6 (Arhgef6), mRNA.	73341	NM_152801.1	NM_152801.1
A	A	P/M	P/M	ILMN_2682984	Cacna1c	calcium channel, voltage-dependent, L type, alpha 1C subunit		NM_009781.1	NM_009781.1
A	A	P/M	P/M	ILMN_2811737	Casp4	Mus musculus caspase 4, apoptosis-related cysteine peptidase (Casp4), mRNA.	12363	NM_007609.1	NM_007609.1
A	A	P/M		ILMN_1216226	Cbln2	Mus musculus cerebellin 2 precursor protein (Cbln2), mRNA.	12405	NM_172633.3	NM_172633.3
A	A	P/M	P/M	ILMN_1212607	Cradd	Mus musculus CASP2 and RIPK1 domain containing adaptor with death domain (Cradd), mRNA.	12905	NM_009950.2	NM_009950.2
A	A	P/M		ILMN_2688262	Csf2rb	Mus musculus colony stimulating factor 2 receptor, beta, low-affinity (granulocyte-macrophage) (Csf2rb), mRNA.	12983	NM_007780.3	NM_007780.3
A	A	P/M		ILMN_1227152	D430036O16Rik			AK085103	
A	A	P/M	P/M	ILMN_2669234	Dbh	dopamine beta hydroxylase		NM_138942.2	NM_138942.2
A	A	P/M		ILMN_1222377	E130003A04Rik			AK053263	
A	A	P/M		ILMN_2936911	Fat2	Mus musculus FAT tumor suppressor homolog 2 (Drosophila) (Fat2), mRNA.	245827	NM_001029988.2	NM_001029988.2
A	A	P/M	P/M	ILMN_1226182	G430005B15Rik			AK089927	
A	A	P/M	P	ILMN_2756771	Gmfb	Mus musculus glia maturation factor, beta (Gmfb), mRNA.	63985	NM_022023.1	NM_022023.1
A	A	P/M		ILMN_2867076	H2-Oa	Mus musculus histocompatibility 2, O region alpha locus (H2-Oa), mRNA.	15001	NM_008206.1	NM_008206.1
A	A	P/M	P/M	ILMN_2647044	Ift74	Mus musculus intraflagellar transport 74 homolog (Chlamydomonas) (Ift74), mRNA.	67694	NM_026319.2	NM_026319.2
A	A	P/M		ILMN_1215523	LOC629554	PREDICTED: Mus musculus hypothetical protein LOC629554 (LOC629554), misc RNA.	629554	XR_035395.1	XR_035395.1
A	A	P/M	P/A	ILMN_2985249	LOC631002	Mus musculus synovial sarcoma, X member B family member (LOC631002), mRNA.	631002	NM_001081565.1	NM_001081565.1
A	A	P/M	P	ILMN_1248363	Mpdz	multiple PDZ domain protein		AK051782	
A	A	P/M	P	ILMN_2900151	Olfr123	Mus musculus olfactory receptor 123 (Olfr123), mRNA.	258623	NM_146630.1	NM_146630.1
A	A	P/M		ILMN_2864972	Olfr1330	Mus musculus olfactory receptor 1330 (Olfr1330), mRNA.	258331	NM_146334.1	NM_146334.1
A	A	P/M		ILMN_2875857	Olfr462	Mus musculus olfactory receptor 462 (Olfr462), mRNA.	258406	NM_146411.1	NM_146411.1
A	A	P/M		ILMN_3160741	Olfr610	Mus musculus olfactory receptor 610 (Olfr610), mRNA.	259085	NM_147081.1	NM_147081.1
A	A	P/M	M	ILMN_2679243	Pitx3	Mus musculus paired-like homeodomain transcription factor 3 (Pitx3), mRNA.	18742	NM_008852.3	NM_008852.3
A	A	P/M	P	ILMN_3102085	Plekha4	Mus musculus pleckstrin homology domain containing, family A (phosphoinositide binding specific) member 4 (Plekha4), mRNA	69217	NM_148927.1	NM_148927.1
A	A	P/M		ILMN_2587335	Sec15l2	PREDICTED: Mus musculus SEC15-like 2 (S. cerevisiae), transcript variant 4 (Sec15l2), mRNA.	75914	XM_898417.2	XM_898417.2
A	A	P/M	P	ILMN_1232875	Sgk3	Mus musculus serum/glucocorticoid regulated kinase 3 (Sgk3), transcript variant 2, mRNA.	170755	NM_177547.3	NM_177547.3
A	A	P/M		ILMN_1220865	Spink8	Mus musculus serine peptidase inhibitor, Kazal type 8 (Spink8), mRNA.	78709	NM_183136.2	NM_183136.2
A	A	P/M		ILMN_2927910	Tll2	Mus musculus tolloid-like 2 (Tll2), mRNA.	24087	NM_011904.1	NM_011904.1
A	A	P/M	P/M	ILMN_1229131	Wfdc3	Mus musculus WAP four-disulfide core domain 3 (Wfdc3), mRNA.	71856	NM_027961.1	NM_027961.1
A	A	P/M	P/M	ILMN_2829268	Zbtb32	Mus musculus zinc finger and BTB domain containing 32 (Zbtb32), mRNA.	58206	NM_021397.1	NM_021397.1
A	A	P	P	ILMN_1227592	2900097C17Rik	PREDICTED: Mus musculus RIKEN cDNA 2900097C17 gene (2900097C17Rik), mRNA.	347740	XM_001480665.1	XM_001480665.1
A	A	P	P	ILMN_2453058	3110099E03Rik	RIKEN cDNA 3110099E03 gene		XM_488954	
A	A	P	P	ILMN_2629897	4632404H12Rik	Mus musculus RIKEN cDNA 4632404H12 gene (4632404H12Rik), mRNA.	74034	NM_028726.1	NM_028726.1
A	A	P	P	ILMN_1237645	5031438A03Rik	RIKEN cDNA 5031438A03 gene		AK077267	
A	A	P	P	ILMN_2552605	5330429D05Rik			AK030541	
A	A	P	P	ILMN_1249943	6430530L21Rik			AK032382	
A	A	P	P	ILMN_2621448	Adh7	Mus musculus alcohol dehydrogenase 7 (class IV), mu or sigma polypeptide (Adh7), mRNA.	11529	NM_009626.3	NM_009626.3
A	A	P	P/M	ILMN_1230114	Agpt			AK030531	
A	A	P	P	ILMN_1217254	Atrnl1	Mus musculus attractin like 1 (Atrnl1), mRNA.	226255	NM_181415.3	NM_181415.3
A	A	P	P	ILMN_1238508	B930007E16Rik			AK046947	
A	A	P	P	ILMN_2555588	C030030A07Rik	Mus musculus RIKEN cDNA C030030A07 gene (C030030A07Rik), mRNA.	654818	NM_001039558.2	NM_001039558.2
A	A	P	P	ILMN_2638461	Csn1s1	Mus musculus casein alpha s1 (Csn1s1), mRNA.	12990	NM_007784.2	NM_007784.2
A	A	P	P	ILMN_1238486	Ctsc	Mus musculus cathepsin C (Ctsc), mRNA.	13032	NM_009982.3	NM_009982.3
A	A	P	P	ILMN_1215172	Folh1	folate hydrolase 1		AK090289	
A	A	P	P/M	ILMN_3160727	Gm694	Mus musculus gene model 694, (NCBI) (Gm694), mRNA.	277744	NM_001033374.2	NM_001033374.2
A	A	P	P/M	ILMN_1241785	Larp2	Mus musculus La ribonucleoprotein domain family, member 2 (Larp2), mRNA.	214048	NM_001040399.1	NM_001040399.1
A	A	P	P	ILMN_2718499	Lass4	Mus musculus longevity assurance homolog 4 (S. cerevisiae) (Lass4), mRNA.	67260	NM_026058.3	NM_026058.3
A	A	P	P	ILMN_1226622	LOC100041522	PREDICTED: Mus musculus similar to 4933409K07Rik protein (LOC100041522), mRNA.	100041522	XM_001473293.1	XM_001473293.1
A	A	P	P	ILMN_2728504	Lrrc18	Mus musculus leucine rich repeat containing 18 (Lrrc18), mRNA.	67580	NM_026253.3	NM_026253.3
A	A	P	P	ILMN_1249815	Rplp0	Mus musculus ribosomal protein, large, P0 (Rplp0), mRNA.	11837	NM_007475.4	NM_007475.4
A	A	P	P	ILMN_2706101	Snx5	Mus musculus sorting nexin 5 (Snx5), mRNA. XM_001004302	69178	NM_024225.4	NM_024225.4
A	A	P	P/M	ILMN_1229630	Trim30	Mus musculus tripartite motif-containing 30 (Trim30), mRNA.	20128	NM_009099.2	NM_009099.2
A	A	P	P	ILMN_2418855	Ugt1a6a	Mus musculus UDP glucuronosyltransferase 1 family, polypeptide A6A (Ugt1a6a), mRNA.	94284	NM_145079.2	NM_145079.2
A	A	P	P	ILMN_1226508	Usp25	Mus musculus ubiquitin specific peptidase 25 (Usp25), mRNA.	30940	NM_013918.2	NM_013918.2
A	A	P	P	ILMN_1258734	Wnt2	Mus musculus wingless-related MMTV integration site 2 (Wnt2), mRNA.	22413	NM_023653.4	NM_023653.4
A	A	P	P	ILMN_2466926	Zfp235	Mus musculus zinc finger protein 235 (Zfp235), mRNA.	56525	NM_019941.2	NM_019941.2
A	A	M	P/M/A	ILMN_3098894	BC061212	Mus musculus cDNA sequence BC061212 (BC061212), mRNA.	381724	NM_198667.1	NM_198667.1
A	A	M		ILMN_3064339	Ccdc27	Mus musculus coiled-coil domain containing 27 (Ccdc27), mRNA.	381580	NM_001033455.1	NM_001033455.1

Supplementary Table S6. Continued…

A	A	P/M	ILMN_1229178	5530402F09Rik			AK030703	
A	A	P/M	ILMN_1227905	C630015A19Rik			AK083111	
A	A	P	ILMN_2800813	Cabc1	Mus musculus chaperone, ABC1 activity of bc1 complex like (S. pombe) (Cabc1), nuclear gene encoding mitochondrial protein,	67426	NM_023341.2	NM_023341.2
A	A	P/M	ILMN_2702687	Hoxd10	Mus musculus homeo box D10 (Hoxd10), mRNA.	15430	NM_013554.3	NM_013554.3
A	A	P/M	ILMN_1224906	V1re5	Mus musculus vomeronasal 1 receptor, E5 (V1re5), mRNA.	171228	NM_134194.1	NM_134194.1
A	A	P/M	ILMN_2467320	Zfp277	Mus musculus zinc finger protein 277 (Zfp277), transcript variant 1, mRNA.	246196	NM_172575.2	NM_172575.2
A	A	P	ILMN_2806377	Zfp458	Mus musculus zinc finger protein 458 (Zfp458), mRNA.	238690	NM_001001152.2	NM_001001152.2

A: absent; M: marginal; P: present. No call is indicated if any of the samples in a sample group failed to pass QC.

Supplementary Table S7. Genes present in B6 and absent or marginal in 129.

Strain 129		Strain B6							
WT	nm1054	WT	nm1054	Probe ID	Gene Name	Gene Description	Entrez Gene I	Accession Number	RefSeq ID
A	A	P	P	ILMN_1227592	2900097C17Rik	PREDICTED: Mus musculus RIKEN cDNA 2900097C17 gene (2900097C17Rik), mRNA.	347740	XM_001480665.1	XM_001480665.1
A	A	P	P	ILMN_2453058	3110099E03Rik	RIKEN cDNA 3110099E03 gene		XM_488954	
A	A	P	P	ILMN_2629897	4632404H12Rik	Mus musculus RIKEN cDNA 4632404H12 gene (4632404H12Rik), mRNA.	74034	NM_028726.1	NM_028726.1
A	A	P	P	ILMN_1237645	5031438A03Rik	RIKEN cDNA 5031438A03 gene		AK077267	
A	A	P	P	ILMN_1249943	6430530L21Rik			AK032382	
A	A	P	P	ILMN_2621448	Adh7	Mus musculus alcohol dehydrogenase 7 (class IV), mu or sigma polypeptide (Adh7), mRNA.	11529	NM_009626.3	NM_009626.3
A	A	P	P	ILMN_1217254	Atrnl1	Mus musculus attractin like 1 (Atrnl1), mRNA.	226255	NM_181415.3	NM_181415.3
A	A	P	P	ILMN_1238508	B930007E16Rik			AK046947	
A	A	P	P	ILMN_2555588	C030030A07Rik	Mus musculus RIKEN cDNA C030030A07 gene (C030030A07Rik), mRNA.	654818	NM_001039558.2	NM_001039558.2
A	A	P	P	ILMN_1238486	Ctsc	Mus musculus cathepsin C (Ctsc), mRNA.	13032	NM_009982.3	NM_009982.3
A	A	P	P	ILMN_1215172	Folh1	folate hydrolase 1		AK090289	
A	A	P	P	ILMN_2718499	Lass4	Mus musculus longevity assurance homolog 4 (S. cerevisiae) (Lass4), mRNA.	67260	NM_026058.3	NM_026058.3
A	A	P	P	ILMN_1226622	LOC100041522	PREDICTED: Mus musculus similar to 4933409K07Rik protein (LOC100041522), mRNA.	100041522	XM_001473293.1	XM_001473293.1
A	A	P	P	ILMN_2728504	Lrrc18	Mus musculus leucine rich repeat containing 18 (Lrrc18), mRNA.	67580	NM_026253.3	NM_026253.3
A	A	P	P	ILMN_2706101	Snx5	Mus musculus sorting nexin 5 (Snx5), mRNA. XM_001004302	69178	NM_024225.4	NM_024225.4
A	A	P	P	ILMN_2418855	Ugt1a6a	Mus musculus UDP glucuronosyltransferase 1 family, polypeptide A6A (Ugt1a6a), mRNA.	94284	NM_145079.2	NM_145079.2
A	A	P	P	ILMN_1226508	Usp25	Mus musculus ubiquitin specific peptidase 25 (Usp25), mRNA.	30940	NM_013918.2	NM_013918.2
A	A	P	P	ILMN_1258734	Wnt2	Mus musculus wingless-related MMTV integration site 2 (Wnt2), mRNA.	22413	NM_023653.4	NM_023653.4
A	A	P	P	ILMN_2466926	Zfp235	Mus musculus zinc finger protein 235 (Zfp235), mRNA.	56525	NM_019941.2	NM_019941.2
A/M	A	P	P	ILMN_2436600	5430433J05Rik	PREDICTED: Mus musculus RIKEN cDNA 5430433J05 gene (5430433J05Rik), mRNA.	71363	XM_001480709.1	XM_001480709.1
A/M	A	P	P	ILMN_2463137	6330418B08Rik	RIKEN cDNA 6330418B08 gene			
A/M	A	P	P	ILMN_2586997	A630040K04Rik			AK080298	
A/M	A	P	P	ILMN_1227240	Cdkn2b	Mus musculus cyclin-dependent kinase inhibitor 2B (p15, inhibits CDK4) (Cdkn2b), mRNA.	12579	NM_007670.3	NM_007670.3
A/M	A	P	P	ILMN_1248257	Lpcat2	Mus musculus lysophosphatidylcholine acyltransferase 2 (Lpcat2), mRNA.	270084	NM_173014.1	NM_173014.1
A/M	A	P	P	ILMN_2455192	Zfp68	Mus musculus zinc finger protein 68 (Zfp68), transcript variant 1, mRNA.	24135	NM_013844.2	NM_013844.2
	A	P	P	ILMN_2585368	F730021A14Rik			AK089391	
A/M	A	P	P	ILMN_1219705	D130027K14Rik			AK083865	
A/M	A	P	P	ILMN_1226174	Entpd4	ectonucleoside triphosphate diphosphohydrolase 4		NM_026174	
A/M	A	P	P	ILMN_2772515	Hpcal1	Mus musculus hippocalcin-like 1 (Hpcal1), mRNA.	53602	NM_016677.3	NM_016677.3
A/M	A	P	P	ILMN_1218923	LOC100041932	PREDICTED: Mus musculus hypothetical protein LOC100041932 (LOC100041932), mRNA.	100041932	XM_001477842.1	XM_001477842.1
A/M	A	P	P	ILMN_2789294	Tmem25	Mus musculus transmembrane protein 25 (Tmem25), mRNA.	71687	NM_027865.1	NM_027865.1
A/M	A	P	P	ILMN_2544126	2010005E20Rik	Prmt3, protein arginine N-methyltransferase 3		AK008118	
A/M	A	P	P	ILMN_2836173	Arv1	Mus musculus ARV1 homolog (yeast) (Arv1), mRNA.	68865	NM_026855.1	NM_026855.1
A/M	A	P	P	ILMN_2994806	H2afj	Mus musculus H2A histone family, member J (H2afj), mRNA.	232440	NM_177688.2	NM_177688.2
	A	P	P	ILMN_2748875	Fcer1g	Fc receptor, IgE, high affinity I, gamma polypeptide		NM_010185.2	NM_010185.2
A	A/M	P	P	ILMN_1218282	5330414O08Rik	Coa4, cytochrome c oxidase assembly factor 4		NM_183270.1	NM_183270.1
A	A/M	P	P	ILMN_2512636	D130086K05Rik	Rfx7, regulatory factor X, 7			
A	A/M	P	P	ILMN_1239696	E130116C07Rik	Riken cDNA E130116C07 gene		AK053631	
A	A/M	P	P	ILMN_2898944	Lrrc57	Mus musculus leucine rich repeat containing 57 (Lrrc57), mRNA.	66606	NM_025657.2	NM_025657.2
A	A/M	P	P	ILMN_1246324	Tmem74	Mus musculus transmembrane protein 74 (Tmem74), mRNA.	239408	NM_175502.3	NM_175502.3
A/M	A/M	P	P	ILMN_1246871	6230412A12Rik			AK031763	
A/M	A/M	P	P	ILMN_2492961	6530401N04Rik	Dtd2, D-tyrosyl-tRNA deacylase 2		NM_029545	
A/M	A/M	P	P	ILMN_2612868	Etnk2	Mus musculus ethanolamine kinase 2 (Etnk2), mRNA.	214253	NM_175443.4	NM_175443.4
A/M	A/M	P	P	ILMN_1241311	LOC194492			XM_112649.1	XM_112649.1
A/M	A/M	P	P	ILMN_2589318	Pou6f1	Mus musculus POU domain, class 6, transcription factor 1 (Pou6f1), mRNA.	19009	NM_010127.3	NM_010127.3
A/M	A/M	P	P	ILMN_2546842	2700017A04Rik	Slf1, SMC5-SMC6 complex localization factor 1		AK012253	
A/M	A/M	P	P	ILMN_1246118	Trim9	Mus musculus tripartite motif protein 9 (Trim9), mRNA.	94090	NM_053167.2	NM_053167.2
A	A/M	P	P	ILMN_1214859	BC027344	Mus musculus cDNA sequence BC027344 (BC027344), mRNA.	233057	NM_173738.2	NM_173738.2
A	A/M	P	P	ILMN_1259577	Cftr	Mus musculus cystic fibrosis transmembrane conductance regulator homolog (Cftr), mRNA.	12638	NM_021050.2	NM_021050.2
A	A/M	P	P	ILMN_1258931	Exosc10	Mus musculus exosome component 10 (Exosc10), mRNA.	50912	NM_016699.1	NM_016699.1
A	A/M	P	P	ILMN_1250991	Kctd21	Mus musculus potassium channel tetramerisation domain containing 21 (Kctd21), mRNA.	622320	NM_001039039.3	NM_001039039.3
A	A/M	P	P	ILMN_2669416	Mgst1	Mus musculus microsomal glutathione S-transferase 1 (Mgst1), mRNA.	56615	NM_019946.4	NM_019946.4
A	A/M	P	P	ILMN_2972585	Pvr	Mus musculus poliovirus receptor (Pvr), mRNA.	52118	NM_027514.1	NM_027514.1
A/M	A/M	P	P	ILMN_1231805	Pik3r4	Mus musculus phosphatidylinositol 3 kinase, regulatory subunit, polypeptide 4, p150 (Pik3r4), mRNA.	75669	NM_001081309.1	NM_001081309.1
	A/M	P	P	ILMN_2560314	A230081P14Rik			AK038992	
A	A/M	P	P	ILMN_2749719	4930515G01Rik	PREDICTED: Mus musculus RIKEN cDNA 4930515G01 gene (4930515G01Rik), misc RNA.	67642	XR_035133.1	XR_035133.1
A	A/M	P	P	ILMN_2446620	Zfp114	Mus musculus zinc finger protein 114 (Zfp114), mRNA.	232966	NM_001029933.2	NM_001029933.2
A/M	A/M	P	P	ILMN_1225662	1700016J18Rik	PREDICTED: Mus musculus RIKEN cDNA 1700016J18 gene (1700016J18Rik), mRNA.	75561	XM_994504.1	XM_994504.1
A/M	A/M	P	P	ILMN_2861743	Cage1	Mus musculus cancer antigen 1 (Cage1), mRNA.	71213	NM_027724.1	NM_027724.1
A/M	A/M	P	P	ILMN_1234703	F730031O20Rik	Cass4, Cas scaffolding protein family member 4		XM_283813	
A/M		P	P	ILMN_1246714	Grik1	glutamate receptor, ionotropic, kainate 1		AK046122	
A/M		P	P	ILMN_2650578	1600002H07Rik	Mus musculus RIKEN cDNA 1600002H07 gene (1600002H07Rik), mRNA.	72016	NM_028056.1	NM_028056.1

Supplementary Table S7. Continued…

A/M		P	P	ILMN_3022911	Kctd14	Mus musculus potassium channel tetramerisation domain containing 14 (Kctd14), mRNA.	233529	NM_001012434.2	NM_001012434.2
A	A/M	P	P	ILMN_1240505	B230213E18Rik			AK045580	
A	A/M	P	P	ILMN_1254328	C030048H21Rik	PREDICTED: Mus musculus RIKEN cDNA C030048H21 gene (C030048H21Rik), mRNA.	77481	XM_975397.2	XM_975397.2
A	A/M	P	P	ILMN_1220620	Hnrpa3	Mus musculus heterogeneous nuclear ribonucleoprotein A3 (Hnrpa3), transcript variant a, mRNA.	229279	NM_198090.1	NM_198090.1
A	A/M	P	P	ILMN_1243014	Zfp706	Mus musculus zinc finger protein 706 (Zfp706), mRNA.	68036	NM_026521.3	NM_026521.3
A/M	A/M	P	P	ILMN_2581860	2410008A19Rik	Smyd3, SET and MYND domain containing 3		AK087728	
A/M	A/M	P	P	ILMN_1246004	2810458H16Rik	Pot1b, protection of telomeres 1B		AK013364	
A/M	A/M	P	P	ILMN_1216281	Map4k5	Mus musculus mitogen-activated protein kinase kinase kinase kinase 5 (Map4k5), transcript variant 2, mRNA.	399510	NM_024275.2	NM_024275.2
A/M	A/M	P	P	ILMN_1223492	Apc	adenomatosis polyposis coli		AK054433	
	A/M	P	P	ILMN_2579927	9130020G22Rik	Nipal3, NIPA-like domain containing 3		AK082376	
A	A/M	P	P	ILMN_1227023	Mdm1	Mus musculus transformed mouse 3T3 cell double minute 1 (Mdm1), transcript variant 2, mRNA.	17245	NM_148922.2	NM_148922.2
A/M	A/M	P	P	ILMN_1252022	C130071M06Rik			AK081721	
A/M	A/M	P	P	ILMN_2628204	Elmo1	Mus musculus engulfment and cell motility 1, ced-12 homolog (C. elegans) (Elmo1), transcript variant 1	140580	NM_080288.1	NM_080288.1
A/M	A/M	P	P	ILMN_1233643	0610007P08Rik	Ercc6l2, excision repair cross-complementing rodent repair deficiency, complementation group 6 like 2		NM_023507.2	NM_023507.2
	A/M	P	P	ILMN_2597332	1700123O20Rik	Mus musculus RIKEN cDNA 1700123O20 gene (1700123O20Rik), mRNA.	58248	NM_021437.1	NM_021437.1
	A/M	P	P	ILMN_2577286	D330012P07Rik			AK052245	
A		P	P	ILMN_2691469	Spata7	Mus musculus spermatogenesis associated 7 (Spata7), mRNA.	104871	NM_178914.3	NM_178914.3
A	A/M	P	P	ILMN_2417991	Ube2i	Mus musculus ubiquitin-conjugating enzyme E2I (Ube2i), mRNA.	22196	NM_011665.3	NM_011665.3
A/M	A/M	P	P	ILMN_2708949	LOC100048251	PREDICTED: Mus musculus hypothetical protein LOC100048251 (LOC100048251), misc RNA.	100048251	XR_034443.1	XR_034443.1
A/M	A/M	P	P	ILMN_2667894	1500035H01Rik	Mus musculus RIKEN cDNA 1500035H01 gene (1500035H01Rik), mRNA.	76568	NM_023831.3	NM_023831.3
A/M	A/M	P	P	ILMN_2491392	Tm7sf3	Mus musculus transmembrane 7 superfamily member 3 (Tm7sf3), mRNA.	67623	NM_026281.2	NM_026281.2
	A/M	P	P	ILMN_2721149	Arl11	Mus musculus ADP-ribosylation factor-like 11 (Arl11), mRNA.	219144	NM_177337.3	NM_177337.3
A/M	M	P	P	ILMN_1243183	2510003D18Rik	Asb17os, ankyrin repeat and SOCS box-containing 17, opposite strand			
A/M	M	P	P	ILMN_2792028	Gpx6	Mus musculus glutathione peroxidase 6 (Gpx6), mRNA.	75512	NM_145451.1	NM_145451.1
A/M	M	P	P	ILMN_1214468	Nkpd1	PREDICTED: Mus musculus NTPase, KAP family P-loop domain containing 1 (Nkpd1), mRNA.	69547	XM_133219.7	XM_133219.7
A/M		P	P	ILMN_1222969	4930404F20Rik			AK015077	
A/M		P	P	ILMN_2714209	A330021E22Rik	Mus musculus RIKEN cDNA A330021E22 gene (A330021E22Rik), mRNA.	207686	NM_172447.2	NM_172447.2
A/M		P	P	ILMN_2663403	LOC100043918	PREDICTED: Mus musculus similar to EBI-1 ligand chemokine (LOC100043918), mRNA.	100043918	XM_001481237.1	XM_001481237.1
A		P	P	ILMN_2605504	LOC100044363	PREDICTED: Mus musculus similar to N-cadherin (LOC100044363), mRNA.	100044363	XM_001471994.1	XM_001471994.1
A/M		P	P	ILMN_2844671	3110043J09Rik	Mus musculus RIKEN cDNA 3110043J09 gene (3110043J09Rik), mRNA.	73167	NM_028455.1	NM_028455.1
A/M		P	P	ILMN_1246293	9330168G06Rik			AK034247	
A/M		P	P	ILMN_1248267	E030030I06Rik	PREDICTED: Mus musculus RIKEN cDNA E030030I06 gene, transcript variant 1 (E030030I06Rik)	319887	XM_286230.5	XM_286230.5
A/M		P	P	ILMN_1233351	Ocm	Mus musculus oncomodulin (Ocm), mRNA.	18261	NM_033039.2	NM_033039.2
A/M		P	P	ILMN_1230426	LOC380670			XM_354575.1	XM_354575.1
A/M		P	P	ILMN_1225710	BC023179	Mus musculus cDNA sequence BC023179 (BC023179), mRNA.	232855	NM_145577.2	NM_145577.2
A/M		P	P	ILMN_1259318	Synpo2	PREDICTED: Mus musculus synaptopodin 2, transcript variant 3 (Synpo2), mRNA.	118449	XM_915986.2	XM_915986.2
A		P	P	ILMN_1217331	Mcm6	Mus musculus minichromosome maintenance deficient 6 (MIS5 homolog, S. pombe)	17219	NM_008567.1	NM_008567.1
A/M	A	P	P	ILMN_1249785	C030037D09Rik	PREDICTED: Mus musculus RIKEN cDNA C030037D09 gene (C030037D09Rik), mRNA.	193280	XM_972794.1	XM_972794.1
A/M	A	P	P	ILMN_1224021	Ccdc109b	Mus musculus coiled-coil domain containing 109B (Ccdc109b), mRNA.	66815	NM_025779.2	NM_025779.2
A/M	A	P	P	ILMN_2605503	Cdh2	cadherin 2		NM_007664.1	NM_007664.1
A/M	A	P	P	ILMN_2656189	Nkiras1	Mus musculus NFKB inhibitor interacting Ras-like protein 1 (Nkiras1), mRNA.	69721	NM_023526.3	NM_023526.3
A/M	A	P	P	ILMN_1242556	Rd3	Mus musculus retinal degeneration 3 (Rd3), mRNA.	74023	NM_023727.2	NM_023727.2
A/M	A	P	P	ILMN_1243843	Trnt1	tRNA nucleotidyl transferase, CCA-adding, 1		NM_027296	
A/M	A	P	P	ILMN_3161206	Zfp85-rs1	Mus musculus zinc finger protein 85, related sequence 1 (Zfp85-rs1), mRNA.	22746	NM_001001130.2	NM_001001130.2
M	A	P	P	ILMN_1248493	LOC100043609	PREDICTED: Mus musculus hypothetical protein LOC100043609 (LOC100043609), mRNA.	100043609	XM_001480340.1	XM_001480340.1
M	A	P	P	ILMN_2937261	Mod1	Mus musculus malic enzyme, supernatant (Mod1), mRNA.	17436	NM_008615.1	NM_008615.1
	A	P	P	ILMN_1219248	E130114F22Rik			AK053612	
A/M	A/M	P	P	ILMN_1253384	Pkig	Mus musculus protein kinase inhibitor, gamma (Pkig), transcript variant 2, mRNA.	18769	NM_001039390.1	NM_001039390.1
M	A/M	P	P	ILMN_2647204	Tatdn3	PREDICTED: Mus musculus TatD DNase domain containing 3 (Tatdn3), mRNA.	68972	XM_129661.10	XM_129661.10
M/P	A/M	P	P	ILMN_3162925	C130026I21Rik	Mus musculus RIKEN cDNA C130026I21 gene (C130026I21Rik), transcript variant 1, mRNA.	620078	NM_175219.3	NM_175219.3
A/M	A/M	P	P	ILMN_2586789	5730420E01Rik			AK077483	
A/M	A/M	P	P	ILMN_1243942	Adam17	Mus musculus a disintegrin and metallopeptidase domain 17 (Adam17), mRNA.	11491	NM_009615.5	NM_009615.5
A/M	A/M	P	P	ILMN_2993334	Plekhf1	Mus musculus pleckstrin homology domain containing, family F (with FYVE domain) member 1	72287	NM_024413.1	NM_024413.1
A/M	A/M	P	P	ILMN_1232697	Zfp93	Mus musculus zinc finger protein 93 (Zfp93), mRNA.	22755	NM_009567.4	NM_009567.4
	A/M	P	P	ILMN_2673507	1700060C20Rik	RIKEN cDNA 1700060C20 gene		XM_149230.1	XM_149230.1
A/M	A/M	P	P	ILMN_1247156	Apoa2	Mus musculus apolipoprotein A-II (Apoa2), mRNA.	11807	NM_013474.1	NM_013474.1
A/M	A/M	P	P	ILMN_1230499	Pmscl1	exosome component 9		AK083794	
A/M	A/M	P	P	ILMN_2638914	D330027H18Rik	PREDICTED: Mus musculus RIKEN cDNA D330027H18 gene (D330027H18Rik), mRNA.	101359	XM_149840.5	XM_149840.5
A/M	A/M	P	P	ILMN_2614351	Serhl	Mus musculus serine hydrolase-like (Serhl), mRNA.	68607	NM_023475.2	NM_023475.2
A/M		P	P	ILMN_2939277	Sncg	Mus musculus synuclein, gamma (Sncg), mRNA.	20618	NM_011430.1	NM_011430.1
A/M	A/M	P	P	ILMN_2692986	2810410P22Rik	Arl5a, ADP-ribosylation factor-like 5A		NM_182994.1	NM_182994.1
A/M	A/M	P	P	ILMN_2775957	Apoa2	Mus musculus apolipoprotein A-II (Apoa2), mRNA.	11807	NM_013474.1	NM_013474.1
A/M	A/M	P	P	ILMN_1257700	Kcnj6	Potassium inwardly-rectifying channel, subfamily J, member 6 (Kcnj6), transcript variant Girk2B	16522	NM_001025585.2	NM_001025585.2
A/M	A/M	P	P	ILMN_1246491	A230065C20Rik	RIKEN cDNA A230065C20 gene		AK038810	
A/M	A/M	P	P	ILMN_1238124	C330018M05Rik			AK049278	
A/M	A/M	P	P	ILMN_1216575	LOC329506			XM_283758.1	XM_283758.1
A/M	A/M	P	P	ILMN_2821247	Necab1	Mus musculus N-terminal EF-hand calcium binding protein 1 (Necab1), mRNA.	69352	NM_178617.3	NM_178617.3

Supplementary Table S7. Continued…

A/M	A/M	P	P	ILMN_2969845	Ccl21c	Mus musculus chemokine (C-C motif) ligand 21c (leucine) (Ccl21c), mRNA.	65956	NM_023052.1	NM_023052.1
A/M	A/M	P	P	ILMN_3008858	Ctsc	Mus musculus cathepsin C (Ctsc), mRNA.	13032	NM_009982.2	NM_009982.2
M	A/M	P	P	ILMN_2554110	Entpd4	Mus musculus ectonucleoside triphosphate diphosphohydrolase 4 (Entpd4), mRNA.	67464	NM_026174.2	NM_026174.2
M	A/M	P	P	ILMN_2544305	Nnmt	nicotinamide N-methyltransferase		AK006371	
A/M	A/M	P	P	ILMN_3008859	Ctsc	Mus musculus cathepsin C (Ctsc), mRNA.	13032	NM_009982.2	NM_009982.2
A/M	A/M	P	P	ILMN_2684093	Rec8	Mus musculus REC8 homolog (yeast) (Rec8), mRNA.	56739	NM_020002.2	NM_020002.2
M	A/M	P	P	ILMN_1229141	4933406L09Rik	RIKEN cDNA 4930452B06 gene		AK016701	
M	A/M	P	P	ILMN_1227377	Synj2	synaptojanin 2		AK014584	
	A/M	P	P	ILMN_2844848	2410018C20Rik	Mus musculus RIKEN cDNA 2410018C20 gene (2410018C20Rik), mRNA.	67873	NM_026423.2	NM_026423.2
	A/M	P	P	ILMN_2569511	A630004L17Rik			AK041354	
	A/M	P	P	ILMN_2623315	LOC100040601	PREDICTED: Mus musculus hypothetical protein LOC100040601 (LOC100040601), mRNA.	100040601	XM_001475251.1	XM_001475251.1
	A/M	P	P	ILMN_2846821	EG328280	Mus musculus predicted gene, EG328280 (EG328280), mRNA.	328280	NM_001015506.1	NM_001015506.1
A/M	A/M	P	P	ILMN_1217896	C230084O18Rik			AK048948	
A/M	A/M	P	P	ILMN_2675125	Epha10	Mus musculus Eph receptor A10 (Epha10), mRNA.	230735	NM_177671.4	NM_177671.4
M	A/M	P	P	ILMN_2769490	5430435G22Rik	Mus musculus RIKEN cDNA 5430435G22 gene (5430435G22Rik), mRNA.	226421	NM_145509.2	NM_145509.2
M	A/M	P	P	ILMN_1215394	Adpgk	Mus musculus ADP-dependent glucokinase (Adpgk), mRNA.	72141	NM_028121.2	NM_028121.2
M	A/M	P	P	ILMN_2466121	Twistnb	Mus musculus TWIST neighbor (Twistnb), mRNA.	28071	NM_172253.2	NM_172253.2
	A/M	P	P	ILMN_1246768	A830026B15Rik			AK043729	
A/M		P	P	ILMN_1221176	4833419P04Rik			AK029383	
A/M		P	P	ILMN_3090731	Pkig	Mus musculus protein kinase inhibitor, gamma (Pkig), transcript variant 3, mRNA.	18769	NM_001039391.1	NM_001039391.1
A/M		P	P	ILMN_2501455	D430041D05Rik	Mus musculus RIKEN cDNA D430041D05 gene (D430041D05Rik), mRNA.	241589	NM_001033347.2	NM_001033347.2
A/M		P	P	ILMN_2725091	A930033H14Rik	PREDICTED: Mus musculus RIKEN cDNA A930033H14 gene (A930033H14Rik), misc RNA.	320700	XR_005131.2	XR_005131.2
A/M		P	P	ILMN_2858477	4930502E18Rik	Mus musculus RIKEN cDNA 4930502E18 gene (4930502E18Rik), mRNA.	75013	NM_029142.1	NM_029142.1
A/M		P	P	ILMN_2483650	LOC545835	PREDICTED: Mus musculus similar to T-cell receptor beta chain V region CTL-L17 precursor (LOC545835), mRNA.	545835	XM_620301.3	XM_620301.3
A/M		P	P	ILMN_1216631	Acat3	acetyl-Coenzyme A acetyltransferase 3		NM_153151.1	NM_153151.1
A/M		P	P	ILMN_3008399	Glp2r	Mus musculus glucagon-like peptide 2 receptor (Glp2r), mRNA.	93896	NM_175681.2	NM_175681.2
A/M		P	P	ILMN_2821118	Ccr7	Mus musculus chemokine (C-C motif) receptor 7 (Ccr7), mRNA.	12775	NM_007719.2	NM_007719.2
A/M		P	P	ILMN_1243388	Slc5a7	Mus musculus solute carrier family 5 (choline transporter), member 7 (Slc5a7), mRNA.	63993	NM_022025.3	NM_022025.3
A/M		P	P	ILMN_2625831	5830435K17Rik	SPT2, Suppressor of Ty, domain containing 1 (S. cerevisiae)		NM_175318.2	NM_175318.2
A/M		P	P	ILMN_1223179	H2-T23	Mus musculus histocompatibility 2, T region locus 23 (H2-T23), mRNA.	15040	NM_010398.3	NM_010398.3
A/M		P	P	ILMN_1215814	LOC215996	PREDICTED: Mus musculus hypothetical LOC215996 (LOC215996), mRNA.	215996	XR_002594.1	XR_002594.1
	A	P	P	ILMN_1240895	Mtap2	Map2, microtubule-associated protein 2		AK086484	
	A/M	P	P	ILMN_2628236	A330021E22Rik	Mus musculus RIKEN cDNA A330021E22 gene (A330021E22Rik), mRNA.	207686	NM_172447.2	NM_172447.2
	A/M	P	P	ILMN_1221182	D130067N22Rik			AK051727	
	A/M	P	P	ILMN_1257803	A130095K04Rik			AK038322	
	A/M	P	P	ILMN_1258275	3830421F03Rik	Pvr, poliovirus receptor		AK014446	
	A/M	P	P	ILMN_1215845	LOC381925	Plpp4, phospholipid phosphatase 4		XM_355946.1	XM_355946.1
	A/M	P	P	ILMN_1256701	2900016B01Rik	PREDICTED: Mus musculus RIKEN cDNA 2900016B01 gene (2900016B01Rik), mRNA.	67262	XM_001474596.1	XM_001474596.1
	A/M	P	P	ILMN_2945607	Zfp26	Mus musculus zinc finger protein 26 (Zfp26), mRNA. XM_974728 XM_977451 XM_977486	22688	NM_011753.1	NM_011753.1
	A/M	P	P	ILMN_1240123	Dclre1c	Mus musculus DNA cross-link repair 1C, PSO2 homolog (S. cerevisiae) (Dclre1c), transcript variant 1, mRNA.	227525	NM_146114.2	NM_146114.2
	A/M	P	P	ILMN_1214767	Tulp3	tubby-like protein 3		AK035363	
	A/M	P	P	ILMN_2640542	Colec11	Mus musculus collectin sub-family member 11 (Colec11), mRNA.	71693	NM_027866.1	NM_027866.1
	A/M	P	P	ILMN_3004302	Rnf41	Mus musculus ring finger protein 41 (Rnf41), mRNA.	67588	NM_026259.2	NM_026259.2
	A/M	P	P	ILMN_2602139	LOC100041504	PREDICTED: Mus musculus similar to beta chemokine Exodus-2 (LOC100041504), mRNA.	100041504	XM_001473258.1	XM_001473258.1
	A/M	P	P	ILMN_1244671	EG668643	PREDICTED: Mus musculus predicted gene, EG668643 (EG668643), mRNA.	668643	XM_001480825.1	XM_001480825.1
	A/M	P	P	ILMN_2542048	LOC382555			XM_356566.1	XM_356566.1
	A/M	P	P	ILMN_1251776	B430114K07Rik			AK046591	
	A/M	P	P	ILMN_1236165	E330021D16Rik	Mus musculus RIKEN cDNA E330021D16 gene (E330021D16Rik), mRNA.	243676	NM_175527.2	NM_175527.2

A: absent; M: marginal; P: present. No call is indicated if any of the samples in a sample group failed to pass QC.

Supplementary Table S8. Genes present in 129 and absent or marginal in B6.

Strain 129		Strain B6							
WT	nm1054	WT	nm1054	Probe ID	Gene Name	Gene Description	Entrez Gene II	Accession Number	RefSeq ID
P	P	A	A	ILMN_2564493	9630038C08Rik			AK036131	
P	P	A	A	ILMN_2844820	Angptl7	Mus musculus angiopoietin-like 7 (Angptl7), mRNA.	654812	NM_001039554.1	NM_001039554.1
P	P	A	A	ILMN_1225440	C430046P22Rik	Whrn, whirlin			
P	P	A	A	ILMN_1241260	D330011G23Rik			AK052232	
P	P	A	A	ILMN_1233293	Gbp1	Mus musculus guanylate binding protein 1 (Gbp1), mRNA.	14468	NM_010259.2	NM_010259.2
P	P	A	A	ILMN_2898886	OTTMUSG00000010673	Mus musculus predicted gene, OTTMUSG00000010673 (OTTMUSG00000010673), mRNA.	433804	NM_001014397.1	NM_001014397.1
P	P	A/M	A	ILMN_2584173	4933414I19Rik			AK077156	
P	P	A/M	A	ILMN_1251337	6230403H02Rik	Sox11, SRY (sex determining region Y)-box 11		XM_356578	
P	P	A/M	A	ILMN_1220879	Slc25a41	Mus musculus solute carrier family 25, member 41 (Slc25a41), mRNA.	103775	NM_175333.3	NM_175333.3
P	P		A/M	ILMN_2960325	Ctse	Mus musculus cathepsin E (Ctse), mRNA.	13034	NM_007799.2	NM_007799.2
P	P		A/M	ILMN_2538772	LOC381716	RIKEN cDNA 1700015F17 gene		XM_358648.1	XM_358648.1
P	P	A/M		ILMN_1260242	Masp2	Mus musculus mannan-binding lectin serine peptidase 2 (Masp2), transcript variant 1, mRNA.	17175	NM_001003893.2	NM_001003893.2
P	P	A/M	A	ILMN_1220253	EG384179	PREDICTED: Mus musculus predicted gene, EG384179 (EG384179), mRNA.	384179	XM_912278.3	XM_912278.3
P	P	A/M	A	ILMN_1225130	LOC385909			XM_358995.1	XM_358995.1
P	P		A/M	ILMN_1251114	BC002216	Cptp, ceramide-1-phosphate transfer protein		AK089229	
P	P	A/M	A	ILMN_1237586	Prss32	Mus musculus protease, serine, 32 (Prss32), mRNA.	69814	NM_027220.1	NM_027220.1
P	P	A/M	A/M	ILMN_2748021	Pcdhgb8	Mus musculus protocadherin gamma subfamily B, 8 (Pcdhgb8), mRNA.	93705	NM_033580.1	NM_033580.1
P	P		A/M	ILMN_1241279	9130024O20Rik			AK078927	
P	P		A	ILMN_2921185	Gzmk	Mus musculus granzyme K (Gzmk), mRNA.	14945	NM_008196.1	NM_008196.1
P	P		A/M	ILMN_2757824	Sv2c	PREDICTED: Mus musculus synaptic vesicle glycoprotein 2c, transcript variant 2 (Sv2c), mRNA.	75209	XM_914033.2	XM_914033.2
P	P	A	A/M	ILMN_2712084	LOC100044150	PREDICTED: Mus musculus hypothetical protein LOC100044150 (LOC100044150), mRNA.	100044150	XM_001471789.1	XM_001471789.1
P	P	A	A/M	ILMN_3156419	Nusap1	Mus musculus nucleolar and spindle associated protein 1 (Nusap1), transcript variant 2, mRNA.	108907	NM_001042652.1	NM_001042652.1
P	P	A	A/M	ILMN_1257368	Gm566	PREDICTED: Mus musculus gene model 566, (NCBI), transcript variant 2 (Gm566), misc RNA.	229672	XR_005162.1	XR_005162.1
P	P	A/M	A/M	ILMN_2524436	2410078J06Rik	Sult6b1, sulfotransferase family, cytosolic, 6B, member 1		XM_356960	
P	P		A/M	ILMN_1245262	LOC100047135	PREDICTED: Mus musculus hypothetical protein LOC100047135 (LOC100047135), mRNA.	100047135	XM_001478025.1	XM_001478025.1
P	P		A/M	ILMN_2614067	LOC100047958	PREDICTED: Mus musculus similar to WD repeat domain 63 (LOC100047958), mRNA.	100047958	XM_001479234.1	XM_001479234.1
P	P	A/M	A/M	ILMN_1226504	Ppm1d	protein phosphatase 1D magnesium-dependent, delta isoform		AK053727	
P	P	A/M	A/M	ILMN_2758440	Gab2	Mus musculus growth factor receptor bound protein 2-associated protein 2 (Gab2), mRNA.	14389	NM_010248.1	NM_010248.1
P	P	M	A/M	ILMN_1238778	4921528H16Rik	Lrguk, leucine-rich repeats and guanylate kinase domain containing		XM_133060.2	XM_133060.2
P	P		A/M	ILMN_2708107	Camk4	Mus musculus calcium/calmodulin-dependent protein kinase IV (Camk4), mRNA.	12326	NM_009793.2	NM_009793.2
P	P		A/M	ILMN_2427324	Gcnt2	Mus musculus glucosaminyl (N-acetyl) transferase 2, I-branching enzyme (Gcnt2), transcript variant 3, mRNA.	14538	NM_133219.1	NM_133219.1
P	P		A/M	ILMN_2530607	LOC214900			XM_148779.2	XM_148779.2
P	P		A/M	ILMN_2541080	LOC384786			XM_357866.1	XM_357866.1
P	P		A/M	ILMN_1243645	LOC386537			XM_359381.1	XM_359381.1
P	P		A/M	ILMN_1248718	Cma2	Mus musculus chymase 2, mast cell (Cma2), mRNA.	545055	NM_001024714.2	NM_001024714.2
P	P		A/M	ILMN_3071356	Dbi	Mus musculus diazepam binding inhibitor (Dbi), transcript variant 2, mRNA.	13167	NM_007830.3	NM_007830.3
P	P	A	A/M	ILMN_2643394	Sox11	Mus musculus SRY-box containing gene 11 (Sox11), mRNA.	20666	NM_009234.5	NM_009234.5
P	P	A	A/M	ILMN_1238867	9430038G21Rik			AK034787	
P	P	A	A/M	ILMN_2749529	Itga9	Mus musculus integrin alpha 9 (Itga9), mRNA.	104099	NM_133721.1	NM_133721.1
P	P	A	A/M	ILMN_2946653	Klk1b22	Mus musculus kallikrein 1-related peptidase b22 (Klk1b22), mRNA.	13646	NM_010114.1	NM_010114.1
P	P	A/M	A/M	ILMN_2650180	4931440B09Rik	Mus musculus RIKEN cDNA 4931440B09 gene (4931440B09Rik), mRNA.	71003	NM_027644.1	NM_027644.1
P	P	A/M	A/M	ILMN_2577664	Fcgr2b	Fc receptor, IgG, low affinity IIb		AK080885	
P	P	A/M	A/M	ILMN_2537948	LOC624610	PREDICTED: Mus musculus hypothetical protein LOC624610 (LOC624610), mRNA.	624610	XM_992064.1	XM_992064.1
P	P		A/M	ILMN_2701460	Defb10	Mus musculus defensin beta 10 (Defb10), mRNA.	246085	NM_139225.1	NM_139225.1
P	P	A/M	A/M	ILMN_2487104	scl0002690.1_1			AK004440.1	AK004440.1
P	P		A/M	ILMN_2556365	E130112J05Rik			AK053589	
P	P	A/M	A/M	ILMN_1220063	EG666668	PREDICTED: Mus musculus predicted gene, EG666668 (EG666668), mRNA.	666668	XM_985281.1	XM_985281.1
P	P	M	A/M	ILMN_2705860	Tpsab1	Mus musculus tryptase alpha/beta 1 (Tpsab1), mRNA.	17230	NM_031187.2	NM_031187.2
P	P		A/M	ILMN_2689973	9430024E24Rik	PREDICTED: Mus musculus RIKEN cDNA 9430024E24 gene (9430024E24Rik), mRNA.	77257	XM_001479724.1	XM_001479724.1
P	P		A/M	ILMN_1252848	LOC100040318	PREDICTED: Mus musculus similar to spermiogenesis specific transcript on the Y 1 (LOC100040318), mRNA.	100040318	XM_001473928.1	XM_001473928.1
P	P		A/M	ILMN_2477136	2010003K15Rik	Smlr1, small leucine-rich protein 1			
P	P		A/M	ILMN_2526703	LOC329305			XM_287066.1	XM_287066.1
P	P		A/M	ILMN_1212960	LOC381186			XM_355114.1	XM_355114.1
P	P		A/M	ILMN_2521893	scl0015365.1_6			BC064076.1	BC064076.1
P	P		A/M	ILMN_1236537	0610008F07Rik	Hnf4aos, hepatic nuclear factor 4 alpha, opposite strand		NM_197983.1	NM_197983.1
P	P	A	A/M	ILMN_1213906	LOC384565			XM_357712.1	XM_357712.1
P	P	A/M	A/M	ILMN_2533815	LOC327767	Gm5080, predicted gene 5080		XM_284874.2	XM_284874.2
P	P	A/M	M	ILMN_2527156	LOC381345			XM_355308.1	XM_355308.1
P	P		A/M	ILMN_1250780	6030436K07Rik			AK031463	
P	P	A/M	A/M	ILMN_1245410	LOC382809			XM_356688.1	XM_356688.1
P	P	A/M	A/M	ILMN_2438221	Nphp3	nephronophthisis 3 (adolescent)		NM_028721	
P	P	A/M	A/M	ILMN_1225981	D830015G02Rik	Mhrt, myosin heavy chain associated RNA transcript		AK052874	

Supplementary Table S8. Continued…

P	P	A/M	M	ILMN_1220581	Fip1l1	Mus musculus FIP1 like 1 (S. cerevisiae) (Fip1l1), mRNA.	66899	NM_024183.4	NM_024183.4
P	P	A/M	M	ILMN_3160874	Pla2g4c	Mus musculus phospholipase A2, group IVC (cytosolic, calcium-independent) (Pla2g4c), mRNA.	232889	NM_001004762.2	NM_001004762.2
P	P	A/M	M	ILMN_1225604	C430020H24Rik			AK049536	
P	P	A/M	M	ILMN_1226083	Ifltd1	Mus musculus intermediate filament tail domain containing 1 (Ifltd1), mRNA.	74071	NM_028742.2	NM_028742.2
P	P	A/M		ILMN_2910258	Bnc1	Mus musculus basonuclin 1 (Bnc1), mRNA.	12173	NM_007562.1	NM_007562.1
P	P		A/M	ILMN_2568421	A530019I06Rik			AK040715	
P	P	A/M	A/M	ILMN_2737130	Klf12	Mus musculus Kruppel-like factor 12 (Klf12), mRNA.	16597	NM_010636.3	NM_010636.3
P	P	A/M	M	ILMN_1230160	1700020O03Rik	Gpatch2l, G patch domain containing 2 like			
P	P	A/M	M	ILMN_2573376	E330026G11Rik			AK054445	
P	P	M	A/M	ILMN_2731035	Dhrs7c	Mus musculus dehydrogenase/reductase (SDR family) member 7C (Dhrs7c), mRNA.	68460	NM_001013013.1	NM_001013013.1
P	P		A/M	ILMN_2483771	C920027I18Rik	Cald1, caldesmon 1			
P	P		A/M	ILMN_1219538	Fut11	Mus musculus fucosyltransferase 11 (Fut11), mRNA.	73068	NM_028428.2	NM_028428.2
P	P	A/M		ILMN_1237812	scl0001916.1_1504			AK039260.1	AK039260.1
P	P	A/M		ILMN_1258507	Cep110	PREDICTED: Mus musculus centrosomal protein 110 (Cep110), mRNA.	26920	XM_981952.1	XM_981952.1
P	P	A/M		ILMN_2781784	Osr2	Mus musculus odd-skipped related 2 (Drosophila) (Osr2), mRNA.	107587	NM_054049.1	NM_054049.1
P	P	A/M		ILMN_2766727	Chrm2	Mus musculus cholinergic receptor, muscarinic 2, cardiac (Chrm2), mRNA.	243764	NM_203491.1	NM_203491.1
P	P	A/M		ILMN_2548754	4930515K21Rik	Zfp451, zinc finger protein 451		AK015797	
P	P	A		ILMN_2995263	Gm1082	Mus musculus gene model 1082, (NCBI) (Gm1082), mRNA.	381868	NM_001004161.1	NM_001004161.1
P	P	A/M		ILMN_1216682	A730041H09Rik			AK042935	
P	P	A/M		ILMN_2620114	Ren1	Mus musculus renin 1 structural (Ren1), mRNA.	19701	NM_031192.2	NM_031192.2
P	P	A/M		ILMN_2463969	3110052M02Rik	Zfp983, zinc finger protein 983		XM_484601	
P	P	A/M		ILMN_2548703	4930438B07Rik	Lrriq3, leucine-rich repeats and IQ motif containing 3		AK015333	
P	P	A/M		ILMN_1233642	D930048J18Rik			AK086738	
P	P	A/M		ILMN_2533481	LOC272633	Gm5066, predicted gene 5066		XM_195759.3	XM_195759.3
P	P	A/M		ILMN_2540775	LOC382297			XM_356416.1	XM_356416.1
P	P	A/M		ILMN_2537640	LOC384215			XM_357494.1	XM_357494.1
P	P	A/M		ILMN_2636041	1810010D01Rik	RIKEN cDNA 1810010D01 gene		XM_358679.1	XM_358679.1
P	P	A/M		ILMN_1233274	B930014J03Rik	Manba, mannosidase, beta A, lysosomal		AK047057	
P	P	A/M		ILMN_1215909	LOC381305	Rc3h1, RING CCCH (C3H) domains 1		XM_355250.1	XM_355250.1
P	P	A/M		ILMN_1228406	Ugcgl2	Mus musculus UDP-glucose ceramide glucosyltransferase-like 2 (Ugcgl2), mRNA.	66435	NM_001081252.1	NM_001081252.1
P	P	A/M		ILMN_2701207	1810029B16Rik	Mus musculus RIKEN cDNA 1810029B16 gene (1810029B16Rik), mRNA.	66282	NM_025465.2	NM_025465.2
P	P	A/M		ILMN_1226949	Adora3	adenosine A3 receptor		NM_009631	
P	P	A/M		ILMN_1238170	A930039A15Rik	RIKEN cDNA A930039A15 gene		AK080751	
P	P	A/M		ILMN_2587650	C330046L10Rik	Usp49, ubiquitin specific peptidase 49		AK082872	
P	P	A/M		ILMN_1242415	Kcnj9	Mus musculus potassium inwardly-rectifying channel, subfamily J, member 9 (Kcnj9), mRNA.	16524	NM_008429.2	NM_008429.2
P	P	A/M		ILMN_1241493	Prdm2	Mus musculus PR domain containing 2, with ZNF domain (Prdm2), mRNA.	110593	NM_001081355.1	NM_001081355.1

A: absent; M: marginal; P: present. No call is indicated if any of the samples in a sample group failed to pass QC.
